# Therapeutic Black Phosphorus Nanosheets Elicit Neutrophil Response for Enhanced Tumor Suppression

**DOI:** 10.1002/advs.202414779

**Published:** 2025-01-22

**Authors:** Jing Wang, Weiqiang Yu, Hui Shen, Yanxiang Sang, Hongjie Zhang, Benyan Zheng, Xue Peng, Yuan Hu, Xiaopeng Ma, Zhenye Yang, Fazhi Yu

**Affiliations:** ^1^ Department of General Surgery The First Affiliated Hospital of University of Science and Technology of China Division of Life Sciences and Medicine University of Science and Technology of China Hefei Anhui 230036 P. R. China; ^2^ Department of Digestive Disease The First Affiliated Hospital of University of Science and Technology of China Division of Life Sciences and Medicine University of Science and Technology of China Hefei Anhui 230036 P. R. China; ^3^ Key Laboratory of Immune Response and Immunotherapy School of Basic Medical Sciences Division of Life Sciences and Medicine University of Science and Technology of China Hefei P. R. China; ^4^ State Key Laboratory of Fire Science University of Science and Technology of China Hefei Anhui 230036 P. R. China; ^5^ HIM‐BGI Omics Center Hangzhou Institute of Medicine (HIM) Chinese Academy of Sciences (CAS) Hangzhou P. R. China; ^6^ School of Life Science and Technology China Pharmaceutical University; ^7^ Department of General Surgery The Chinese People's Armed Police Forces Anhui Provincial Corps Hospital Hefei Anhui Province 234000 P. R. China

**Keywords:** black phosphorus, cancer therapy, immunomodulatory, neutrophil

## Abstract

Black phosphorus (BP) has demonstrated potential as a drug carrier and photothermal agent in cancer therapy; however, its intrinsic functions in cancer treatment remain underexplored. This study investigates the immunomodulatory effects of polyethylene glycol‐functionalized BP (BP‐PEG) nanosheets in breast cancer models. Using immunocompetent mouse models‐including 4T1 orthotopic BALB/c mice and MMTV‐PyMT transgenic mice, it is found that BP‐PEG significantly inhibits tumor growth and metastasis without directly inducing cytotoxicity in tumor cells. Mass cytometry analysis reveals that BP‐PEG reshapes the tumor immune microenvironment by recruiting neutrophils. Neutrophil depletion experiments further demonstrate that the antitumor effects of BP‐PEG are dependent on neutrophils. Moreover, bulk and single‐cell RNA sequencing indicate that BP‐PEG is mainly taken up by macrophages, leading to the release of inflammatory factors such as IL1A and CXCL2, which enhance neutrophil recruitment and activation, thereby amplifying the antitumor immune response. Finally, co‐culture assays confirm that BP‐PEG indeed enhances the antitumor activity of neutrophils and natural killer (NK) cells. These findings position BP‐PEG as an immunomodulatory agent capable of reprogramming the tumor microenvironment to promote innate immunity against breast cancer. By stimulating neutrophil‐mediated antitumor activity, BP‐PEG offers a unique therapeutic approach that can potentially enhance the efficacy of existing cancer immunotherapies.

## Graphical abstract

This study explores black phosphorus (BP) nanosheets in breast cancer models, showing their ability to inhibit tumor growth. BP‐PEG enhances immune responses by promoting neutrophil recruitment and reprogramming tumor‐associated neutrophils to an antitumor phenotype, boosting both innate and adaptive antitumor immune activities. Combining BP therapy with immunotherapies or chemotherapies potentially counteract neutrophil‐induced immunosuppression, restoring or enhancing their therapeutic efficacy.

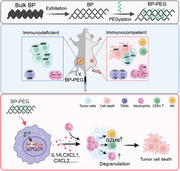

## 1 Introduction

Cancer remains a leading cause of mortality worldwide, necessitating innovative therapeutic strategies.^[^ ^1^ ^]^ 2D nanomaterials have gained significant attention in oncology due to their unique properties and multifunctional applications.^[^ ^2^, ^3^ ^]^ Among them, black phosphorus (BP) has emerged as a promising candidate for cancer therapy owing to its layered structure, tunable electronic properties, and biodegradability.^[^ ^4^, ^5^, ^6^ ^]^ BP consists of corrugated planes of phosphorus atoms connected by strong intralayer P–P bonds and weak interlayer van der Waals forces, allowing exfoliation into few‐layer or monolayer nanosheets.^[^ ^7^ ^]^ This exfoliation results in a layer‐dependent bandgap ranging from 0.3 eV (bulk BP) to ≈2 eV (monolayer BP), endowing the material with remarkable optical and electronic properties suitable for biomedical applications. Notably, BP degrades into nontoxic phosphate ions under physiological conditions.^[^ ^8^ ^]^ Its responsiveness to pondus hydrogenii（pH） and near‐infrared (NIR) radiation enables controlled drug release and reactive oxygen species (ROS) generation, making it attractive for photothermal and photodynamic therapies.^[^ ^9^, ^10^ ^]^

Despite these attributes, most studies have focused on BP's role as a drug carrier or photothermal agent,^[^ ^9^ ^]^ with limited exploration of its direct impact on the tumor microenvironment (TME) and the immune system. The TME is a complex milieu of tumor cells, stromal cells, immune cells, and extracellular matrix components that contribute to tumor progression and therapy resistance.^[^ ^11^, ^12^ ^]^ Immune cells within the TME can suppress or promote tumor growth depending on their activation states.^[^ ^13^, ^14^, ^15^ ^]^ Advancements in cancer immunotherapy have highlighted the potential of modulating the immune system to achieve durable anti‐tumor responses.^[^ ^16^ ^]^ Nanomaterials have been investigated as immunomodulatory agents capable of reprogramming the TME to overcome immunosuppression.^[^ ^17^, ^18^, ^19^ ^]^ Certain nanomaterials act as adjuvants, enhancing antigen presentation and stimulating immune cell activation.^[^ ^20^, ^21^ ^]^ However, BP's immunomodulatory effects, particularly on innate immune cells like neutrophils and macrophages, remain underexplored.

Neutrophils, the most abundant leukocytes in human blood, play critical roles in innate immunity and have been implicated in cancer progression.^[^ ^22^ ^]^ While traditionally considered tumor‐promoting, emerging evidence suggests that neutrophils can exhibit antitumor functions under specific conditions.^[^ ^23^ ^]^ Polarizing neutrophils toward an antitumor phenotype represents a novel therapeutic avenue.^[^ ^24^, ^25^ ^]^ Given BP's potential interactions with immune cells upon administration,^[^ ^26^ ^]^ understanding its effects on the TME's immune components is crucial. BP's degradation products and surface properties may influence immune cell behavior, leading to immune stimulation. Functionalization with biocompatible polymers like polyethylene glycol (PEG) can enhance BP's stability and modulate its biological interactions.^[^ ^27^ ^]^

In this study, we investigated BP's direct immunomodulatory effects in a breast cancer model. We synthesized stable BP‐PEG nanosheets and evaluated their impact on tumor growth and metastasis in immunocompetent mouse models. We focused on how BP‐PEG influences the TME, particularly neutrophil recruitment and activation, and whether these effects contribute to its antitumor activity. Using in vivo experiments, including neutrophil depletion and immunodeficient mouse models, as well as advanced analytical techniques like mass cytometry and single‐cell RNA sequencing, we provide insights into BP‐PEG's immunomodulatory functions. Our findings suggest that BP‐PEG can reshape the tumor's immune landscape, promoting innate immune responses that inhibit tumor progression.

This research enhances our understanding of BP's interactions with the immune system and highlights BP‐PEG's potential as a novel immunotherapeutic agent. By harnessing innate immune responses, specifically neutrophil‐mediated actions, BP‐PEG offers a promising strategy that complements existing therapies and may overcome limitations of conventional chemotherapies, which often promote neutrophil infiltration and induce chemoresistance.^[^ ^28^ ^]^

## 2 Results

### 2.1 BP‐PEG Exhibits Antitumor Activity in Immunocompetent Breast Cancer Mice Model

**Figure 1:**
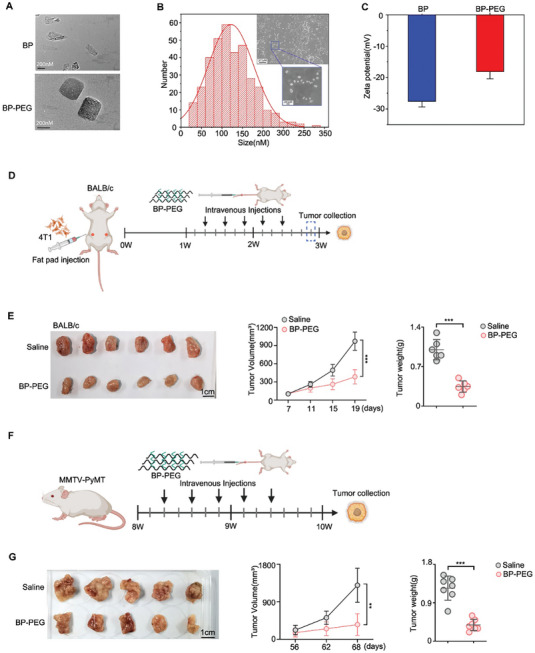
Characterization of BP‐PEG nanosheets and their antitumor activity in an immunocompetent breast cancer mouse model. A) Transmission electron microscopy (TEM) images of black phosphorus (BP) and polyethylene glycol‐modified black phosphorus (BP‐PEG) nanosheets, Scale bar: 200 nm. B) SEM image and particle size distribution of BP‐PEG, Scale bar, 200 µm and 200 nm, respectively. C) Surface charge of BP and BP‐PEG. D) Schematic diagram of the experimental design. 4T1 breast cancer cells were injected into the mammary fat pad of immunocompetent BALB/c mice to establish a syngeneic tumor model. E) BP‐PEG treatment significantly inhibits tumor growth in 4T1 tumor‐bearing BALB/c mice. Left: Tumor growth curves showing tumor volume over time for BP‐PEG‐treated and control groups (*n* = 6 per group). Right: Final tumor weights at the end of the study. F) Schematic diagram of the experimental design. BP‐PEG treatment in MMTV‐PyMT transgenic mice, a model that closely mimics human breast cancer progression. G) Tumor growth and weights of MMTV‐PyMT transgenic mice after BP‐PEG treatment (*n* = 5 per group). Data are presented as mean ± SD; *p*‐values were determined by two‐tailed unpaired *t*‐test; **p < 0.01, ***p < 0.001.

To investigate the antitumor mechanism of black phosphorus (BP), we first synthesized BP and modified it with polyethylene glycol (PEG) to enhance its stability, given BP's inherent instability. Subsequently, we characterized BP‐PEG. Transmission electron microscopy (TEM) images revealed typical 2D sheet‐like structures for both BP and BP‐PEG (**Figure** **1**A). Most BP‐PEG nanosheets were less than 200 nm in size, predominantly around 100 nm, which could ensure the stability and specific tissue targeting of BP‐PEG (Figure 1B). The zeta potential decreased from −27.6 to −18.1 mV after PEG modification (Figure 1C), indicating successful PEG conjugation and enhanced stability of BP. Energy‐dispersive X‐ray spectroscopy (EDS) analysis confirmed the presence of C, N, O, and P elements in BP‐PEG (Figure S1A, Supporting Information). Fourier‐transform infrared (FTIR) spectra showed characteristic peaks corresponding to P═O at 1637 cm⁻¹ and enhanced peaks at 2924 cm⁻¹ due to C─H vibrations from PEG chains (Figure S1B, Supporting Information). X‐ray diffraction (XRD) patterns and Raman spectra confirmed the successful exfoliation of BP into multilayer nanosheets (Figure S1C,D, Supporting Information). UV–vis spectroscopy showed a positive correlation between BP concentration and absorbance (Figure S1E, Supporting Information). Additionally, atomic force microscopy (AFM) images showed that the average size and thickness of PEGylated BP nanosheets (BPNSs) slightly increased due to the PEG‐NH2 coating on the surface (Figure S1F, Supporting Information). Sectional plots revealed that the thickness of BPNSs ranged from 5 to 10 nm, while BPNS‐PEG thickness ranged from 10 to 20 nm (Figure S1F, Supporting Information). Together, these findings confirm the successful synthesis of BP‐PEG nanosheets with improved physiological stability.

We then aimed to explore whether BP‐PEG possesses antitumor activity in breast cancer, as several studies have demonstrated the promise of BP‐based approaches in clinical breast cancer therapy. To this end, we injected 4T1 cells into the fat pad of immunocompetent BALB/C mice to establish a syngeneic model, which is widely used in preclinical cancer research, particularly for studying triple‐negative breast cancer (TNBC), as it replicates many aspects of human TNBC (Figure 1D). Once the average tumor volume reached ≈100 mm^3^, BP‐PEG was administered intravenously, as outlined in the experimental design (Figure 1D). BP‐PEG treatment significantly inhibited the growth of 4T1 breast tumors compared to the control group (Figure 1E), and tumor weights were also reduced (Figure 1E). Histological analysis of major organs showed no obvious toxicity (Figure S1G, Supporting Information).

To determine whether the observed reduction in tumor growth was due to BP alone and not PEG, we treated 4T1 tumor‐bearing BALB/c mice with saline, 200 µL PEG, 10 mg kg^−1^ BP, or 10 mg kg^−1^ BP‐PEG. The results indicated that PEG administration alone had no significant effect on tumor growth when compared to the saline‐treated control group (Figure S1H, Supporting Information). In contrast, BP alone was able to reduce tumor growth, with an effect comparable to that seen with BP‐PEG treatment at the same concentration (Figure S1H, Supporting Information). Additionally, we tested whether reducing the BP‐PEG dose from 10 to 5 mg kg^−1^ would still impact tumor growth. While 5 mg kg^−1^ BP‐PEG also reduced tumor growth, its efficacy was lower than that of the higher dose (Figure S1H, Supporting Information). Based on these findings, we conclude that 10 mg kg^−1^ BP‐PEG is more suitable for evaluating antitumor effects in this model. Additionally, BP‐PEG treatment markedly suppressed lung metastasis of 4T1 cells (Figure S1I, Supporting Information).

To further confirm the inhibitory effect of BP‐PEG on tumor growth in an immunocompetent breast cancer model, we treated MMTV‐PyMT transgenic mice, a model that closely mimics human breast cancer progression, with BP‐PEG. Similar antitumor effects were observed in MMTV‐PyMT transgenic mice (Figure 1F,G). Altogether, these results indicate that BP‐PEG effectively inhibits breast cancer development and metastasis in models that closely mimic human breast cancer.

### 2.2 BP‐PEG Antitumor Growth Dependent on the Immune Microenvironment

To investigate the antitumor mechanism of BP‐PEG, we first examined whether BP‐PEG exerts its function by directly killing tumor cells. We constructed FITC‐labeled BP‐PEG and confirmed that BP‐PEG primarily accumulated in 4T1‐derived tumor tissues in vivo using an animal imaging system (Figure S2A, Supporting Information). Electron microscopy also confirmed that BP‐PEG could be taken up by 4T1 tumor cells in vitro (Figure S2B, Supporting Information).

**Figure 2:**
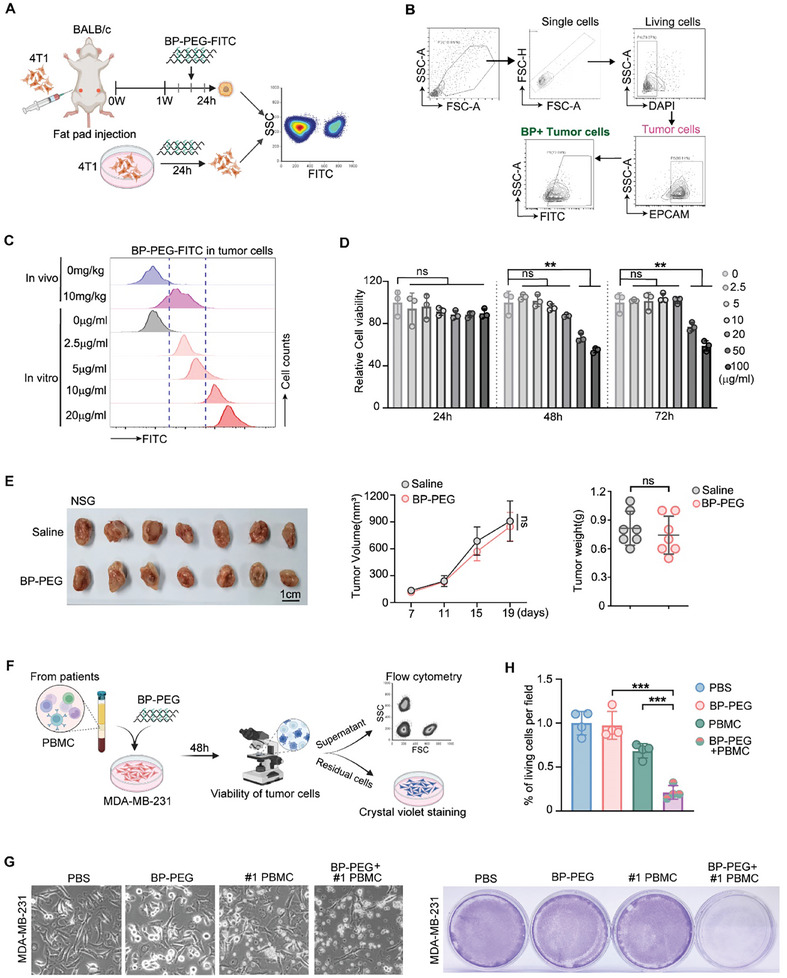
BP‐PEG does not directly kill tumor cells but requires an intact immune system for antitumor activity. A) Schematic diagram illustrating the comparison of BP‐PEG internalization by tumor cells in vivo and in vitro using flow cytometry. B) Flow cytometry gating strategy to identify EPCAM⁺ tumor cells from dissociated tumor tissues and measure FITC‐BP‐PEG internalization 24 h post‐injection. C) Comparison of FITC fluorescence intensity in tumor cells treated in vivo with BP‐PEG (10 mg kg^−1^) and 4T1 cells treated in vitro with varying concentrations of FITC‐BP‐PEG (0, 2.5, 5, 10 µg mL^−1^). The in vivo internalization corresponds to ≈5 µg mL^−1^ in vitro treatment. D) Cell viability assay of 4T1 cells treated with BP‐PEG at concentrations up to 100 µg mL^−1^ for 72 h, showing no significant cytotoxicity. Data are presented as mean ± SD, *p*‐values were determined by two‐tailed Student's *t*‐test (ns, not significant; **p < 0.01). E) Tumor growth in immunodeficient NSG mice bearing 4T1 tumors. Left: Tumor growth curves over time (*n* = 7 per group). Right: Final tumor weights. Data are presented as mean ± SD, ns, not significant. F) Schematic of the co‐culture assay where human MDA‐MB‐231 breast cancer cells were co‐cultured with human PBMCs in the presence or absence of BP‐PEG. G) Representative images of tumor cells after co‐culture and stained with crystal violet. H) Quantification of tumor cell viability after co‐culture. Data are presented as mean ± SD; *p*‐values were determined by two‐tailed unpaired *t*‐test; ***p < 0.001.

Next, we used flow cytometry to quantify the amount of BP‐PEG internalized by tumor cells in vivo and compared it to the uptake observed in vitro (**Figure** **2**A). By dissociating tumor tissue to obtain single cells and labeling tumor cells with the tumor marker EPCAM, we measured the amount of BP‐PEG internalized by tumor cells in vivo 24 h after BP‐PEG injection via tail vein (Figure 2B). We then compared the intensity of FITC internalized by tumor cells in vivo with that of 4T1 cells treated with varying concentrations of FITC‐BP‐PEG in vitro (Figure 2C). The results showed that the majority of tumor cells in vivo had similar FITC intensity as 4T1 cells treated with 5 µg mL^−1^ FITC‐BP‐PEG in vitro (Figure 2C). These data demonstrated that, when treated with 10 mg kg^−1^ FITC‐BP‐PEG, tumor cells in vivo internalized an amount of BP‐PEG comparable to 4T1 cells treated with 5 µg mL^−1^ FITC‐BP‐PEG in vitro. We found that, at this concentration, BP‐PEG did not significantly affect tumor cell proliferation or survival over 72 h (Figure 2D), suggesting that BP‐PEG does not exert direct cytotoxic effects on tumor cells at physiologically relevant concentrations.

Since BP‐PEG does not directly kill tumor cells, we propose that it may inhibit tumor growth in vivo by modulating the tumor microenvironment, particularly the immune environment. To explore whether BP‐PEG exerts its function through the immune system, we utilized immunodeficient NOD scid gamma (NSG) mice bearing 4T1 tumors to evaluate the effects of BP‐PEG (Figure 2E). The experimental procedures were consistent with those performed in BALB/c mice (Figure 1D). We found that BP‐PEG treatment failed to inhibit tumor growth and metastasis in immunodeficient NSG mice bearing 4T1 tumors (Figures 2E and S2C, Supporting Information), indicating that an intact immune system is required for BP‐PEG's antitumor activity.

To further demonstrate that BP‐PEG exerts its antitumor effects primarily through the immune system in the tumor microenvironment, we performed a co‐culture assay. We collected blood from breast cancer patients, isolated peripheral blood mononuclear cells (PBMCs), and co‐cultured MDA‐MB‐231 cells with PBMCs in the presence of BP‐PEG or saline. Flow cytometry was used to analyze the PBMC cell components, revealing a composition similar to that of immune cells found within the tumor microenvironment (Figure S2D, Supporting Information). We then quantified the number of remaining tumor cells after co‐culture using microscopy and violet staining (Figure 2F). The data showed that only minimal tumor cell loss occurred in the BP‐PEG or PBMC treatment alone groups, while >90% of tumor cells were eliminated when BP‐PEG and PBMCs were combined (Figure 2G,H). These findings further suggest that BP‐PEG modulates the immune microenvironment to exert its antitumor effects.

### 2.3 BP‐PEG Specifically Activates Tumor Immune Microenvironments Reshaping Pathway in Immunocompetent Mice

**Figure 3:**
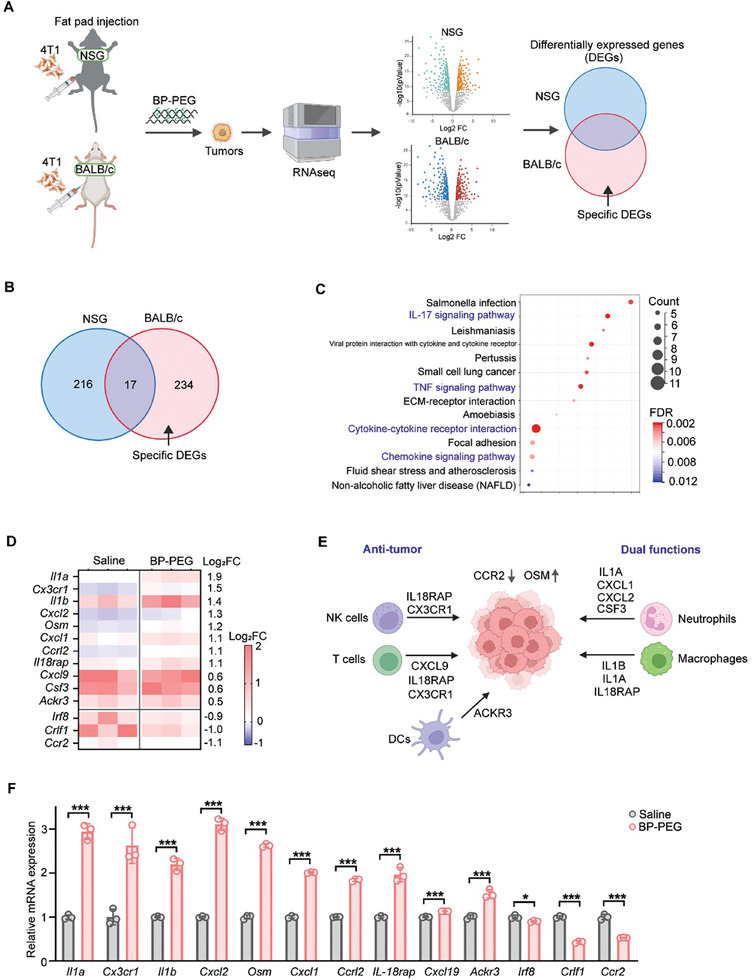
BP‐PEG specifically activates immune regulatory pathways in immunocompetent mice. A) Schematic of bulk RNA sequencing performed on tumor tissues from BALB/c and NSG mice treated with BP‐PEG or saline. B) Venn diagram showing the number of differentially expressed genes (DEGs) in tumors from BALB/c and NSG mice. C) Pathway enrichment analysis of the 234 DEGs uniquely regulated in BALB/c mice tumors (B), highlighting immune‐related pathways such as cytokine–cytokine receptor interaction. D) Heatmap of the DEGs in BALB/c mice tumors involved in immune microenvironment remodeling after BP‐PEG treatment. E) Schematic diagram illustrating the DEGs’ main functions in tumor microenvironment. F) Quantitative PCR validation of the DEGs showed in (D). Data are presented as mean ± SD; *p*‐values were determined by two‐tailed unpaired *t*‐test; *p < 0.05, ***p < 0.001.

Then, we wanted to understand how BP‐PEG exerts antitumor effects in the presence of an intact immune system. To identify the pathways or genes specifically regulated under BP‐PEG treatment, we collected tumor tissues from both BALB/c and NSG mice and performed bulk tissue RNA sequencing for differentially expressed genes (DEGs) analysis (**Figure** **3**A). Our results demonstrated that BP‐PEG altered the gene expression profiles of tumors in both mouse models (Figure S3A,B, Supporting Information). Interestingly, we found that the DEGs in NSG mice tumors overlapped with those in BALB/c mice tumors by only 17 genes, despite the total number of DEGs exceeding 200. This finding suggests that the unique transcription profile in BALB/c mice may be responsible for the antitumor effect of BP‐PEG. Specifically, we identified 234 genes that were differentially expressed only in BALB/c mice (Figure 3B). To understand which pathways are primarily involved in the regulation of BP‐PEG's effects, we conducted pathway enrichment analysis (Figure 3C). The results indicated a significant upregulation of immune‐related pathways, particularly cytokine–cytokine receptor interactions (Figure 3C), which aligns with the notion that BP‐PEG's antitumor function depends on an intact immune system.

Next, we sought to determine which genes were involved in these pathways and their levels of change. Therefore, we conducted a heatmap analysis of the DEGs involved in immune microenvironment remodeling (Figure 3D,E). The analysis revealed that most of these genes were upregulated in the BP‐PEG treatment group and contributed to reshape the tumor immune microenvironment, including genes such as IL1A and CXCL2 (Figure 3D,E). We further confirmed the upregulation or downregulation of these genes by BP‐PEG using qPCR (Figure 3F). In conclusion, our data suggest that BP‐PEG exerts its antitumor effects by specifically activating immune regulatory pathways, thereby reshaping the tumor immune microenvironment.

### 2.4 BP‐PEG Enhances Neutrophil Recruitment to the Tumor Microenvironment

**Figure 4:**
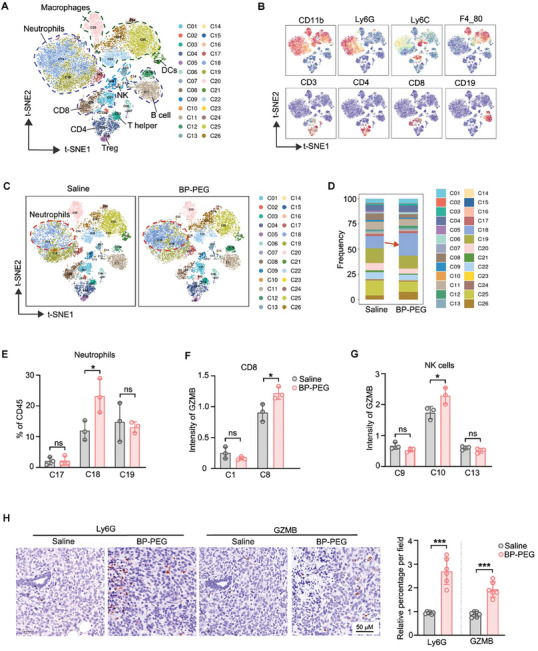
BP‐PEG enhances neutrophil recruitment and activates antitumor immune cells. A) Mass cytometry (CyTOF) analysis of tumor‐infiltrating immune cells from BALB/c mice treated with BP‐PEG or saline. t‐SNE plots display 26 distinct cell clusters. B) t‐SNE visualization of key immune cell defined markers. C) Comparison of immune cell population distributions between saline and BP‐PEG‐treated groups in t‐SNE plots. D) Quantification of immune cell clusters, showing a significant increase in cluster 18 (neutrophils) after BP‐PEG treatment. E) Bar graph illustrating the percentage of different neutrophils clusters among CD45⁺ cells. F,G) The expression intensity of granzyme B (GZMB) in CD8⁺ T cells (F) and NK cells (G). H) Immunohistochemical staining of tumor sections for Ly6G and GZMB. Left: Representative images showing Ly6G and GZMB‐positive cells; Right: Quantification of the relative percentage of Ly6G⁺ and GZMB⁺ cells per field. Data are presented as mean ± SD; *p*‐values were determined by two‐tailed unpaired *t*‐test; ns, not significant; *p < 0.05, ***p < 0.001.

We next sought to determine whether BP‐PEG treatment alters the tumor immune microenvironment. Mass cytometry, an advanced technique capable of simultaneously detecting over 40 markers, was employed to provide a comprehensive analysis of the immune cell populations within the tumor microenvironment. Tumor samples were collected from BALB/C mice, dissociated into single cells, and subjected to mass cytometry (**Figure** **4**A). This analysis identified 26 distinct cell clusters using t‐distributed stochastic neighbor embedding (t‐SNE) (Figure 4A). The clusters were characterized based on marker expression (Figures 4A and S4A, Supporting Information), and key markers were visualized in t‐SNE plots (Figure 4B). We identified neutrophils, macrophages, dendritic cells, B cells, CD8^+^ T cells, CD4^+^ T cells, helper T cells, regulatory T cells (Tregs), and NK cell clusters (Figures 4A and S4A, Supporting Information).

We next compared the composition of immune cell populations between saline and BP‐PEG‐treated groups (Figure 4C). The t‐SNE analysis revealed that cell cluster 18, a subpopulation of neutrophils, exhibited the most significant increase in both number and percentage following BP‐PEG treatment (Figure 4C–E). Posttreatment, this neutrophil subpopulation accounted for over 20% of CD45^+^ cells, emerging as the dominant cell population (Figure 4D,E). Additionally, we analyzed the proportions of other immune cell types and observed a reduction in immunosuppressive cells, specifically regulatory T cells (Tregs) and type M2 macrophages, following BP‐PEG treatment, while other cell populations showed no significant change (Figure S4B–F, Supporting Information). Further, we assessed the antitumor activity of two key immune cell types‐cytotoxic T lymphocytes (CTLs) and natural killer (NK) cells‐by examining the expression of the cytotoxic marker granzyme B (GZMB). The data revealed a substantial upregulation of GZMB intensity in the predominant subpopulations of CD8^+^ T cells and NK cells (Figure 4F,G), indicating that BP‐PEG treatment enhances the antitumor activity of these immune cells.

To further confirm the increase in neutrophil numbers, we performed immunohistochemical staining of tumor tissues using the neutrophil marker Ly6G (Figure 4H). Quantification of neutrophil ratios in the tissue confirmed a significant increase in both the number and percentage of neutrophils following BP‐PEG treatment (Figure 4H). In addition, we assessed the antitumor activity of immune cells in continuous tissue sections, which had been stained for Ly6G. The results showed a significant increase in GZMB‐positive cells, indicating enhanced antitumor functionality of immune cell after BP‐PEG treatment (Figure 4H). In summary, our findings suggest that BP‐PEG promotes neutrophil recruitment, contributing to the enhanced antitumor activity of effector cells, such as CD8^+^ T cells and NK cells.

### 2.5 Neutrophils Are Essential for BP‐PEG's Antitumor Activity

**Figure 5:**
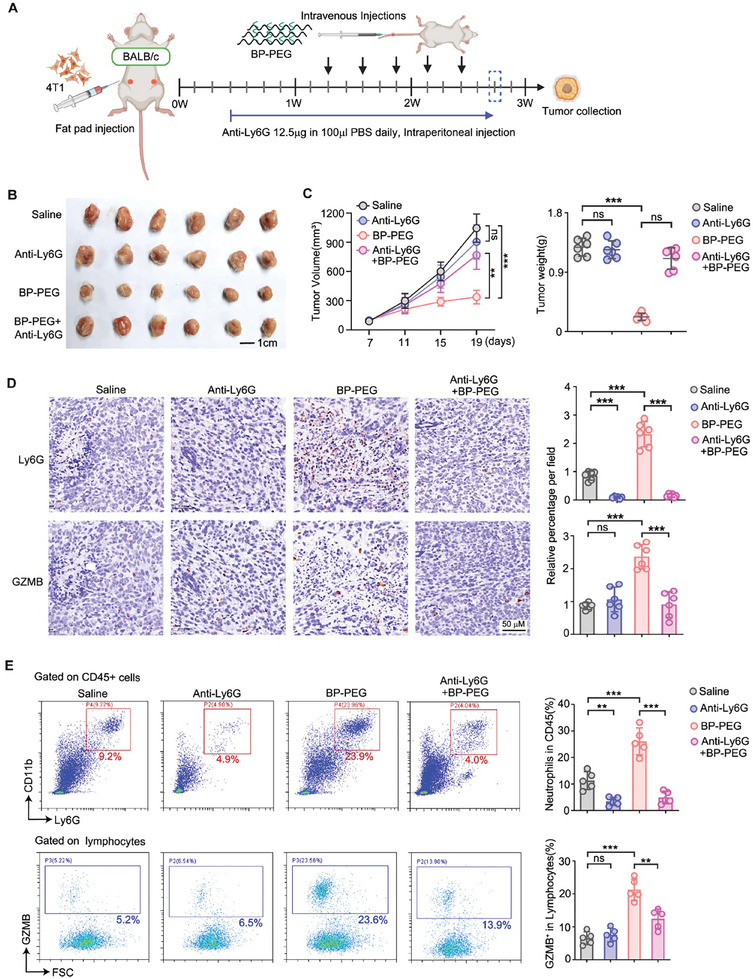
Neutrophils are essential for BP‐PEG's antitumor effects. A) Schematic of the experimental design for neutrophil depletion using anti‐Ly6G antibody in 4T1 tumor‐bearing BALB/c mice. B) Representative tumors from 4T1 cells‐bearing BALB/c mice treated with saline, BP‐PEG, anti‐Ly6G, or BP‐PEG plus anti‐Ly6G. C) Tumor growth curves and the tumor weights of the 4T1 tumors. D) Immunohistochemical staining for Ly6G and GZMB in tumor sections. Left: Representative images showing successful neutrophil depletion and GZMB staining, right: Quantification of the relative percentage of Ly6G⁺ and GZMB⁺ cells per fields. E) Flow cytometry was employed to analyze the percentage of neutrophils within the CD45^+^ cell population and GZMB^+^ cells among lymphocytes isolated from 4T1‐derived tumors. The results were subsequently quantified. Data are presented as mean ± SD; *p*‐values were determined by two‐tailed unpaired *t*‐test; ns, not significant; **p < 0.01, ***p < 0.001.

Given the significant infiltration of neutrophils into tumor tissues following BP‐PEG treatment, we sought to determine whether neutrophils are required for BP‐PEG's antitumor effects. To test this hypothesis, we conducted neutrophil depletion experiments using an anti‐Ly6G antibody in 4T1 tumor‐bearing mice, with a schematic diagram outlining the experimental design (**Figure** **5**A). Starting three days after 4T1 tumor cell inoculation, mice were injected daily with the anti‐Ly6G antibody. Once tumors reached a volume of approximately 100 mm^3^, the mice were randomized into four treatment groups: 1) Saline, 2) BP‐PEG, 3) anti‐Ly6G, or 4) a combination of BP‐PEG and anti‐Ly6G (Figure 5A,B). Consistent with previous studies,^[^ ^29^ ^]^ anti‐Ly6G treatment alone had minimal impact on tumor growth and weight (Figure 5B,C). While BP‐PEG effectively inhibited tumor growth and reduced tumor weight, this effect was largely reversed in the mice treated with the combination of BP‐PEG and anti‐Ly6G (Figure 5B,C). Additionally, while BP‐PEG treatment markedly suppressed lung metastasis of 4T1 cells, neutrophil depletion upregulated lung metastasis (Figure S5A, Supporting Information).

We confirmed the efficiency of neutrophil depletion using immunohistochemistry (IHC) staining and flow cytometry, which showed a near‐complete absence of Ly6G‐positive cells in the anti‐Ly6G‐treated group, confirming successful neutrophil depletion (Figure 5D,E). Furthermore, immunohistochemical staining and flow cytometry for granzyme B showed no substantial difference in the percentage of GZMB‐positive cells between the control and neutrophil‐depleted groups. However, the percentage of GZMB‐positive cells increased with BP‐PEG treatment but decreased following neutrophil depletion (Figure 5D,E), indicating a reduction in antitumor immune responses associated with neutrophil depletion. Altogether, these findings underscore the critical role of neutrophils in mediating BP‐PEG‐induced antitumor immunity.

### 2.6 BP‐PEG Enhances Neutrophil Recruitment Through Macrophage‐Mediated Inflammatory Signaling Pathways

**Figure 6:**
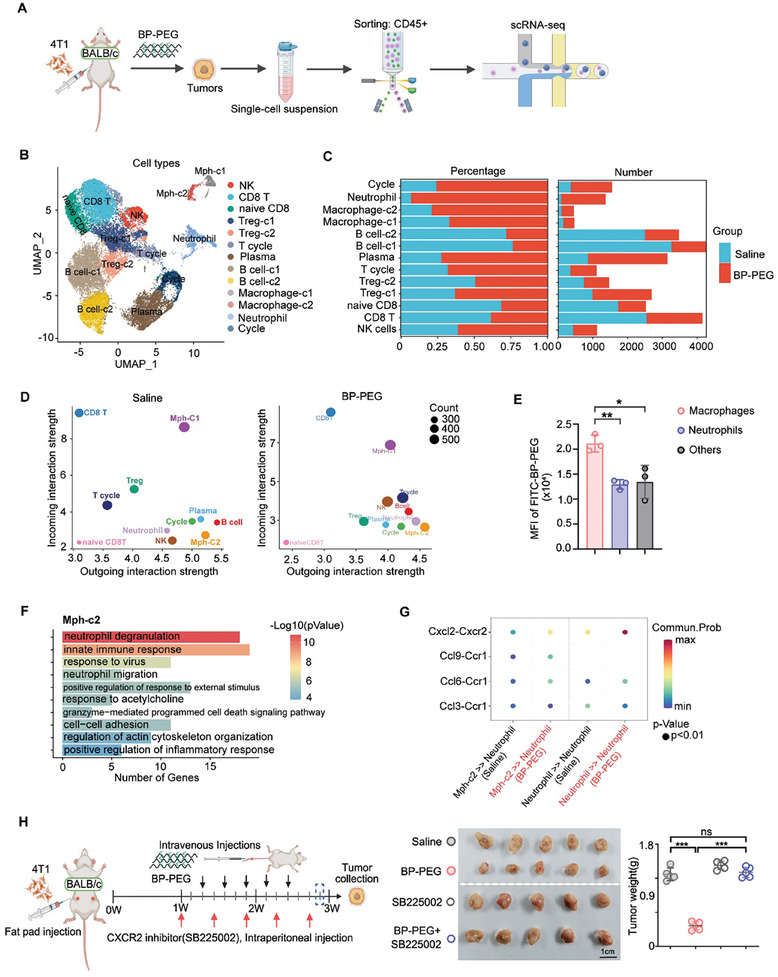
BP‐PEG promotes neutrophil recruitment via macrophage‐mediated inflammatory signaling. A) Workflow for single‐cell RNA sequencing (scRNA‐seq) of CD45⁺ immune cells isolated from tumors of BALB/c mice, comparing those treated with BP‐PEG and saline. B) UMAP plots showing the clustering of immune cells based on treatment conditions. C) Bar graph quantifying the proportions of each immune cell type in tumors treated with saline versus BP‐PEG. D) Analysis of cell–cell interactions, highlighting the strengths of incoming and outgoing interactions among immune cells under saline and BP‐PEG treatments. E) Quantification of mean fluorescence intensity of BP‐PEG‐FITC in myeloid cells within tumor tissues, Data are presented as mean ± SD; *p < 0.05, **p < 0.01. F) Gene Ontology (GO) pathway enrichment analysis of macrophages, based on upregulated genes observed in BP‐PEG‐treated samples compared to saline‐treated samples. G) Cell–cell communication networks through the CXCL–CCR signaling pathway between macrophages and neutrophils, using upregulated genes from BP‐PEG treatment relative to saline treatment. H) A schematic diagram of the experimental design is provided. Saline, SB225002 (10 mg kg^−1^), and BP‐PEG (10 mg kg^−1^) were administered to 4T1 tumor‐bearing BALB/c mice in separate treatment groups (*n* = 5 per group). Tumor images were captured, and tumor weights were analyzed at the end of the study. Data are presented as mean ± SD; *p*‐values were determined by two‐tailed unpaired *t*‐test; ns, not significant; ***p < 0.001.

To further understand how BP‐PEG treatment regulates neutrophil infiltration in tumor tissues, we collected tumors from BALB/c mice treated with either saline or BP‐PEG. CD45‐positive cells were isolated for single‐cell RNA sequencing to identify immune cell populations and analyze their gene expression profiles (**Figure** **6**A). UMAP clustering of all sequenced cells revealed a notable enrichment of several immune cell subsets‐including neutrophils, macrophages, and NK cells‐following BP‐PEG treatment (Figures 6B,C and S6A, Supporting Information). Then using the scRNA‐seq data, we explored the immune cell interactions that may contribute to enhanced neutrophil infiltration and a productive antitumor immune response. Cell–cell interaction analysis provides insights into interactions enriched in BP‐PEG‐treated mice, helping to elucidate the mechanism by which BP‐PEG promotes neutrophil infiltration and a productive antitumor immune response. Cells with high incoming interaction strength might be key responders, while those with high outgoing interaction strength could be major communicators in the system. The results showed that under saline treatment, CD8⁺ T cells and macrophage‐like cells (Mph‐C1) exhibited high incoming interaction strength (Figure 6D), suggesting they are primary targets or responders in the interaction network. B cells and macrophage‐like cells (Mph‐C2) had high outgoing interaction strength, indicating they could be major communicators in the system (Figure 6D). Under BP‐PEG treatment, although CD8⁺ T cells and Mph‐C1 still displayed high incoming interaction strength, Mph‐C2 and neutrophils became the cells with major outgoing interaction strength (Figure 6D), indicating they emerged as significant communicators in the system, especially macrophages.

Given that macrophages have high phagocytic function, we propose that when BP‐PEG is injected into mice, it is first engulfed by macrophages, which increases the inflammatory response in these cells. This response leads to the production of various cytokines that promote neutrophil infiltration and reshape the antitumor environment. To test this hypothesis, we detected the distribution of BP‐PEG‐FITC in tumors using flow cytometry (Figure S6B, Supporting Information). The results showed that macrophages indeed contained the highest level of BP‐PEG‐FITC among myeloid cells (Figure 6E), which is consistent with their primary role in phagocytosis. Altogether, we identified macrophages as likely initiators of interactions among immune cells in the tumor microenvironment (TME), based on the high predicted outgoing interaction strength under BP‐PEG treatment.

We then analyzed functional changes in immune cells using pathway enrichment analysis. In macrophages, genes involved in neutrophil migration were upregulated under BP‐PEG treatment, along with genes related to the regulation of neutrophil degranulation, innate immune response, and positive regulation of the inflammatory response (Figure 6F). These results demonstrate that BP‐PEG uptake by macrophages indeed promotes inflammatory signaling pathways, which in turn promote neutrophil migration and function. In bulk tissue RNA sequencing, we found that cytokines related to neutrophils‐such as IL1A, CXCL1, CXCL2, and CSF3‐were upregulated in BP‐PEG‐treated tumors. According to scRNA‐seq data, the inflammatory related pathway was also upregulated, and CXCL2 was also found to be upregulated in mixed CD45 positive cells and was predominantly expressed by macrophages and neutrophils, indicating that CXCL2 may contribute to neutrophil infiltration under BP‐PEG treatment (Figure S6C, Supporting Information). Additionally, using ELISA to detect CXCL2 expression in THP‐1 cells, we confirmed that CXCL2 is produced by macrophages and upregulated by BP‐PEG (Figure S6D, Supporting Information).

We next analyzed the CXCL‐CCR signaling pathways between macrophages and neutrophils. The results showed that CXCL2‐CXCR2, CCL9‐CCR1, CCL6‐CCR1, and CCL3‐CCR1 signaling pathways were enhanced under BP‐PEG treatment (Figure 6G). Notably, the communication probability within the CXCL2–CXCR2 signaling pathway was significantly upregulated, with CXCL2 expression elevated under BP‐PEG treatment. To further investigate the role of this pathway, we analyzed the expression of CXCL2 receptors, CXCR1 and CXCR2, in the tumor microenvironment using scRNA‐seq data. Our analysis revealed that CXCR2 was highly expressed, predominantly on neutrophils (Figure S6E, Supporting Information), suggesting that CXCL2 primarily exerts its function through interaction with CXCR2 on neutrophils. To further investigate whether the CXCL2–CXCR2 signaling pathway regulates the antitumor effects of BP‐PEG, we treated mice with the CXCR2 inhibitor SB225002 (Figure 6H). The results showed that SB225002 alone had no impact on tumor growth; however, when mice were treated with both BP‐PEG and SB225002, the antitumor effect of BP‐PEG was almost completely abolished (Figure 6H). These findings demonstrate that the antitumor effects of BP‐PEG are primarily dependent on the CXCL2–CXCR2 signaling pathway. Altogether, our data suggest that macrophages may promote neutrophil infiltration under BP‐PEG treatment through the CXCL–CCR signaling pathway, with a particularly critical role for CXCL2–CXCR2.

### 2.7 BP‐PEG Enhances Tumor‐Killing Ability of Neutrophils and NK Cells

**Figure 7:**
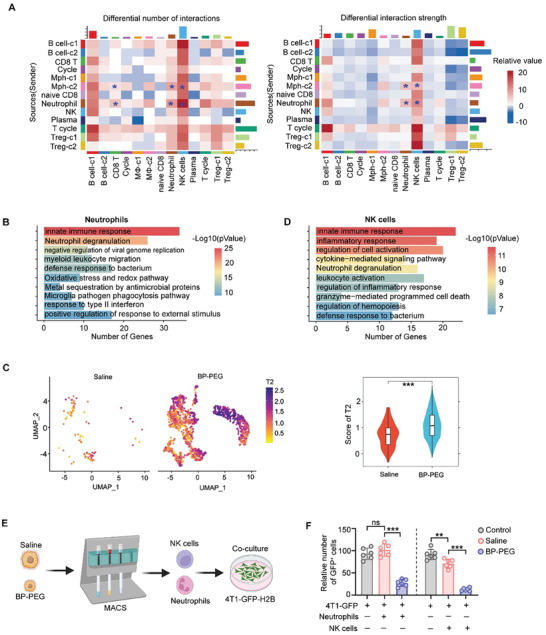
BP‐PEG enhances the tumor‐killing ability of neutrophils and NK Cells. A) Differential number of interactions or interaction strength, visualized as a heatmap, where red increased and blue represents decreased signaling in BP‐PEG compared to saline treated tumors. The top‐colored bar plot represents the sum of a column of values displayed (incoming signaling). The right‐ colored bar plot represents the sum of a row of values (outgoing signaling). B) Gene Ontology (GO) pathway enrichment analysis of neutrophils, based on upregulated genes observed in BP‐PEG‐treated samples compared to saline‐treated samples. C) Analysis of the T2 gene signature expression score in neutrophils. D) Gene Ontology (GO) pathway enrichment analysis of NK cells, based on upregulated genes observed in BP‐PEG‐treated samples compared to saline‐treated samples. E) Experimental setup for assessing the tumor‐killing ability of neutrophils and NK cells isolated from tumors. Immune cells were co‐cultured with GFP‐labeled 4T1 cells. F) Quantification of GFP‐positive tumor cells after co‐culture. Data are presented as mean ± SD; *p*‐values were determined by two‐tailed unpaired *t*‐test; ns, not significant; **p < 0.01, ***p < 0.001.

We then sought to understand how neutrophils regulate antitumor activity. Therefore, we analyzed the differential number of interactions and interaction strengths between neutrophils and key antitumor immune cells, specifically CD8⁺ T cells and NK cells (**Figure** **7**A). The data revealed that the number of interactions between neutrophils and NK cells was higher than that between neutrophils and CD8⁺ T cells or other cells (Figure 7A). Moreover, the interaction strength between neutrophils and NK cells was also notably stronger (Figure 7A). Although interactions between neutrophils and CD8⁺ T cells were also upregulated under BP‐PEG treatment (Figure 7A), the potential ligand–receptor interactions between these cell types were fewer and weaker compared to those between neutrophils and NK cells (Figure S7A, Supporting Information). Immunofluorescence analysis further demonstrated close spatial localization of neutrophils and NK cells under BP‐PEG treatment (Figure S7B, Supporting Information). These findings suggest that neutrophils primarily regulate the antitumor functions of NK cells.

We then analyzed functional changes in neutrophils and NK cells using pathway enrichment analysis. In neutrophils, pathway enrichment analysis revealed upregulation of pathways involved in the innate immune response, neutrophil degranulation, myeloid leukocyte migration, and oxidative stress and redox pathways under BP‐PEG treatment (Figure 7B). These results indicate that neutrophils are involved in regulating cell migration—including their own—and that their antitumor function was enhanced under BP‐PEG treatment, as evidenced by the upregulation of genes involved in neutrophil degranulation and oxidative stress and redox pathways. Additionally, recent studies^[^ ^30^ ^]^ have identified three types of neutrophils in the tumor microenvironment—T1, T2, and T3—where T1 and T2 exert antitumor functions and T3 exhibits protumor activity. To further explore this in our model, we analyzed the neutrophil subtypes and found that the gene signature profiles defining these neutrophil types were also expressed in the neutrophils observed in our study (Figure S7C, Supporting Information). Notably, T1 and T2 neutrophils were upregulated under BP‐PEG treatment, while T3 neutrophils showed no significant change (Figures 7C and S7C, Supporting Information). Altogether, these findings demonstrate that BP‐PEG treatment can reprogram neutrophils to enhance their antitumor function.

Finally, we analyzed pathway enrichment in NK cells. Functions related to granzyme‐mediated programmed cell death were enhanced under BP‐PEG treatment, indicating that antitumor functions were indeed increased under BP‐PEG treatment (Figure 7D). To confirm that BP‐PEG could enhance the tumor‐killing ability of neutrophils and NK cells, we isolated these cells from tumors treated with saline and BP‐PEG using MACS and then co‐cultured them with GFP‐labeled 4T1 cells (Figure 7E). After 24 h, we counted the number of GFP‐positive cells under a microscope. The number of cells per field was analyzed, and the data showed that BP‐PEG indeed enhanced the tumor‐killing ability of neutrophils and NK cells (Figure 7F).

To further demonstrate that BP‐PEG's antitumor effects are dependent on NK cells in vivo, we performed an NK cell depletion assay (Figure S7D,E, Supporting Information). The results showed that depletion of NK cells partially reversed the antitumor effects of BP‐PEG, as tumor weights increased in BP‐PEG‐treated mice lacking NK cells. However, the tumor weights in these mice remained lower than those in the saline‐treated control group (Figure S7D,E, Supporting Information). Altogether, these findings demonstrate that BP‐PEG enhances the tumor‐killing abilities of NK cells and contributes to its antitumor efficacy.

## 3 Discussion

This study demonstrates that black phosphorus‐polyethylene glycol (BP‐PEG) nanosheets exert significant antitumor effects in breast cancer models by modulating the tumor immune microenvironment (TME), specifically by promoting neutrophil recruitment and activation. Unlike conventional chemotherapeutics that directly target tumor cells, BP‐PEG enhances innate immune responses, leading to inhibition of tumor growth and suppression of metastasis. Importantly, BP‐PEG does not directly induce cytotoxicity in tumor cells at physiologically relevant concentrations; instead, its antitumor efficacy is mediated through immune modulation. This is evidenced by the lack of therapeutic effect in immunodeficient NSG mice and the reversal of antitumor activity upon neutrophil depletion in immunocompetent mice, underscoring the pivotal role of neutrophils in BP‐PEG‐mediated tumor suppression. Transcriptomic analyses revealed upregulation of cytokines and chemokines such as IL‐1α and CXCL2, which are crucial for neutrophil recruitment and activation. Furthermore, mass cytometry and single‐cell RNA sequencing confirmed enhanced neutrophil infiltration and functional activation. Thus, BP‐PEG reprograms the TME from immunosuppressive to immunostimulatory, promoting antitumor immunity. Overall, our study demonstrates that neutrophils exhibit remarkable plasticity and can acquire an antitumor phenotype in response to BP‐PEG therapy.

Neutrophils have a dual function in tumor immunity that depends on both context and specific subsets. Historically, they have been considered protumorigenic because they promote angiogenesis, facilitate metastasis, and suppress adaptive immunity.^[^ ^31^ ^]^ Specifically, type 2 tumor‐associated neutrophils have been linked to oncogenesis, tumor progression, metastasis, and the suppression of adaptive immune responses.^[^ ^23^ ^]^ An increased neutrophil‐to‐lymphocyte ratio is viewed as a strong predictor of poor prognosis in various cancer types and is associated with reduced effectiveness of immunotherapies.^[^ ^32^ ^]^

However, neutrophils are a heterogeneous population, and under certain conditions, they can exert antitumor effects.^[^ ^33^, ^34^ ^]^ Under specific conditions of acute inflammation or in response to targeted therapeutic interventions, neutrophils can adopt antitumorigenic phenotypes. For instance, they can enhance antigen presentation and co‐stimulation of T cells, thereby initiating adaptive immune responses.^[^ ^35^ ^]^ Studies have demonstrated that alleviating hypoxia can increase neutrophil infiltration into tumors, leading to tumor cell killing through mechanisms involving NADPH oxidase, reactive oxygen species (ROS), and matrix metalloproteinase‐9 (MMP‐9).^[^ ^30^ ^]^ And neutrophil elastase, which induces apoptosis in tumor cells by cleaving the death domain of CD95.^[^ ^36^ ^]^ Therefore, the role of neutrophils in cancer is complex and highly dependent on the context.

**Figure 8:**
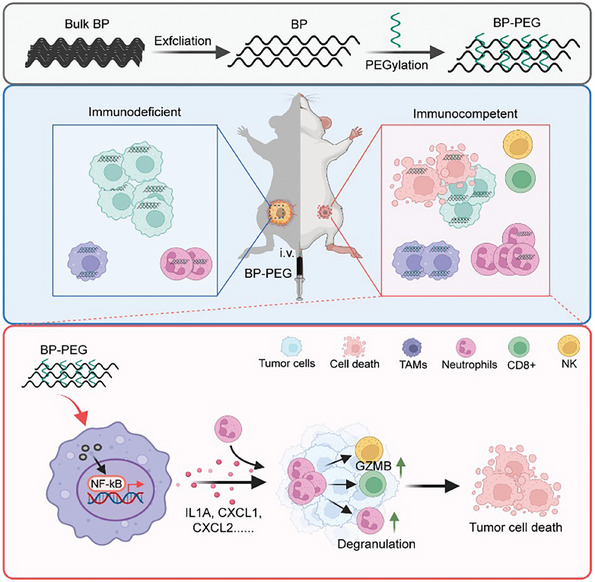
Proposed working model (Created in BioRender. Yu, F. (2024), Agreement number: *TE27PIVUES*).

Our study demonstrated that BP‐PEG nanosheets appear to polarize neutrophils toward an antitumor phenotype, characterized by increased degranulation, production of ROS, and expression of cytotoxic mediators. Upon UMAP and clustering of scRNA‐seq data of tumors from treated mice, we found the neutrophils showed increased in the innate immune response, neutrophil degranulation, myeloid leukocyte migration, and oxidative stress and redox pathways, suggesting that highly differentiated neutrophils adopt an antitumor phenotype. And the interaction between macrophages and neutrophils is critical for the immunomodulatory effects of BP‐PEG (**Figure** **8**). Uptake of BP‐PEG by macrophages activates inflammatory signaling pathways, leading to the secretion of chemokines that recruit neutrophils to the tumor site (Figure 8). This creates a positive feedback loop that amplifies the antitumor immune response. Enhanced interactions among macrophages, neutrophils, natural killer (NK) cells, and CD8⁺ T cells emphasize the importance of immune cell cross‐talk in BP‐PEG‐mediated tumor suppression (Figure 8).

Much of our current understanding of neutrophils' impact on tumor growth and therapy response derives from animal studies where neutrophils are broadly depleted during treatment.^[^ ^37^ ^]^ However, given the emerging evidence of diverse neutrophil subsets with varying functions within tumors, indiscriminate depletion strategies may eliminate both pro‐tumorigenic and antitumorigenic neutrophil populations.^[^ ^25^ ^]^ From a therapeutic standpoint, reprogramming tumor‐associated neutrophils toward an antitumor phenotype may be more effective for tumor control than complete neutrophil depletion.^[^ ^25^ ^]^ Therefore, gaining a deeper understanding of how BP‐PEG influences the acquisition of distinct neutrophil states would allow for selective manipulation of neutrophil subpopulations. This, in turn, would enable us to develop a more nuanced understanding of the role of neutrophils in immunotherapy.

The immunomodulatory properties of BP‐PEG nanosheets offer potential for integration into existing cancer immunotherapies. For example, combining BP‐PEG with immune checkpoint inhibitors may potentiate T‐cell‐mediated antitumor responses by alleviating immunosuppression within the TME.^[^ ^38^, ^39^ ^]^ Furthermore, BP‐PEG's ability to harness innate immune responses, particularly through neutrophil‐mediated mechanisms, offers a complementary strategy to current therapies. This approach may address limitations of conventional chemotherapies, which often exacerbate neutrophil infiltration and contribute to chemoresistance.^[^ ^28^ ^]^ To facilitate clinical translation, comprehensive investigations into the long‐term biodistribution, biodegradation, and clearance of BP‐PEG are essential to establish its safety profile. Additionally, elucidating the molecular mechanisms underlying its immune modulation will be crucial for therapeutic optimization.^[^ ^40^ ^]^ Additionally, exploring the effects of BP‐PEG on other immune cell subsets, such as regulatory T cells and myeloid‐derived suppressor cells, could provide deeper insights into its immunomodulatory capacity.^[^ ^40^ ^]^ Future research should evaluate BP‐PEG in other tumor models and metastatic contexts will help validate its broad applicability. This research enhances our understanding of BP's interactions with the immune system and highlights BP‐PEG's potential as a novel immunotherapeutic agent.

Additionally, BP has emerged as promising nanomaterials for enhancing wound healing processes due to their unique structural, chemical, and functional properties^[^ ^41^, ^42^, ^43^ ^]^ The 2D structure of BP‐PEG offers a high surface area‐to‐volume ratio, facilitating extensive interactions with biological molecules and cells at the wound site.^[^ ^44^ ^]^ This high surface area enhances the adsorption of proteins, cytokines, and growth factors essential for wound healing.^[^ ^41^, ^44^ ^]^ Additionally, BP‐PEG's ability to generate reactive oxygen species (ROS) provides antimicrobial protection while promoting angiogenesis and tissue regeneration, thereby accelerating the wound healing process.^[^ ^41^, ^45^ ^]^ Our study on BP's modulation of neutrophils and macrophages provides key insights into its potential for enhancing immune responses and promoting tissue regeneration. We believe these findings could be leveraged for a range of biomedical applications, especially for treating infected wounds, where BP's dual actions can significantly improve healing outcomes

There are still some limitations of our studies, First, the concentration range of BP‐PEG used in vivo requires further detailed investigation. It is crucial to identify an optimal concentration window where BP‐PEG can effectively elicit an antitumor immune response without causing toxicity to normal tissues. Determining this balance will help ensure the therapeutic efficacy of BP‐PEG while minimizing potential adverse effects on healthy cells. Second, the precise mechanisms by which BP‐PEG is internalized by macrophages and neutrophils, induces the expression of inflammatory factors within the tumor microenvironment, and reprograms neutrophils toward antitumor functions need to be elucidated. Specifically, understanding how BP‐PEG influences neutrophil reprogramming will provide deeper insights into its immunomodulatory effects and enhance the development of targeted cancer therapies. Addressing these limitations in future studies will strengthen the understanding and application of BP‐PEG in cancer immunotherapy.

## 4 Conclusion

In conclusion, BP‐PEG nanosheets effectively modulate the tumor immune microenvironment to inhibit breast cancer growth and metastasis by promoting neutrophil‐dependent antitumor immunity. These findings advance our understanding of BP‐PEG's mechanisms of action and highlight the potential of nanomaterial‐based strategies in reprogramming tumor‐associated neutrophils toward an antitumor phenotype to favor antitumor responses.

## 5 Experimental Section

### Synthesis of BP‐PEG

BP crystals were exfoliated in N‐methyl‐2‐pyrrolidone (NMP) through ultrasonic treatment for 6 h. Following this, 1 mg of BPNS precipitate and 10 mg of PEG‐NH2 were dispersed in deionized water. The resulting mixture underwent sonication for 15 min and was then stirred for 4 h. To remove any unreacted PEG‐NH2, the solution was centrifuged at 12 000 rpm for 10 min. The obtained BP‐PEG dispersion was subsequently washed with deionized water to eliminate residual PEG and solvents, and it was stored in the dark at 4 °C. The characteristics of the BP‐PEG nanosheets were then analyzed using several techniques, including transmission electron microscopy (TEM), zeta potential measurement, energy‐dispersive X‐ray spectroscopy (EDS), Fourier‐transform infrared spectroscopy (FTIR), X‐ray diffraction (XRD), Raman spectroscopy, and UV–vis spectroscopy.

### Cell Line and Cell Culture

4T1 cells were cultured in RPMI 1640 medium (Gibco) supplemented with 10% fetal bovine serum (FBS) and 1% penicillin–streptomycin. And MDA‐MB‐231 cells were maintained in DMEM (Gibco) with the same supplements of 10% FBS and 1% penicillin–streptomycin. All cell lines were incubated in a humidified environment at 37 °C with 5% CO₂.

### Mouse Models

6 to 8‐week‐old female BALB/c and NSG mice were purchased from Shanghai SLAC Laboratory Animal and maintained under specific pathogen‐free conditions with ad libitum access to food and water. All animal experiments were conducted in accordance with the experimental animal guidelines of the University of Science and Technology of China (USTCACUC27120124035). For the orthotopic transplantation experiment, 5 × 10^5^ 4T1‐luciferase cells were resuspended in 15 µL PBS and mixed with an equal volume (15 µL) of Matrigel (Corning, 354 234). The mixture was then injected into the fourth and ninth mammary glands of the mice. When the tumors reached a volume of 100–150 mm^3^, mice were randomly divided into two or four groups. BP‐PEG (10 mg kg^−1^) was administered intravenously every other day. Anti‐Ly6G antibody (Bio X Cell, # BE0075‐1) was administered intraperitoneally at 12.5 µg in 100 µL PBS per mouse daily, starting three days post‐tumor inoculation. Ultra‐LEAF Purified anti‐Asialo‐GM1 Antibody (Biolegend, #146 002) was administered intraperitoneally at 25 µL per mouse every 3 days. SB225002 (MedChemExpress, #HY‐16711) (10 mg kg^−1^) was administered intraperitoneally every 3 days. Tumor growth was monitored using calipers every 3 days, and tumor volume was calculated using the formula: 0.5 × length × width × width.

FVB‐MMTV‐PyMT(no.T004993) transgenic mice were purchased from GemPharmatech. When the tumor volume reached 100–150 mm^3^, mice were randomly assigned to two groups and received either BP‐PEG (10 mg kg^−1^) or vehicle via tail vein injection. Tumor growth was monitored as described above. All mice were euthanized using CO₂ inhalation.

For in vivo imaging, mice were intravenously injected with fluorescein isothiocyanate (FITC)‐labeled BP‐PEG at a dose of 10 mg kg^−1^ 24 h before sacrifice. Organs were collected, and imaging was performed using an Animal Imaging System (IVIS Spectrum).

### Bulk RNA‐seq

Fresh mammary tumors were collected 2 days after treatment with BP‐PEG or vehicle. RNA was extracted from the tumors using TRIzol Reagent (Invitrogen, Carlsbad, CA, USA) following the manufacturer's protocol. Library preparation was conducted using the VAHTS Universal V6 RNA‐seq Library Prep Kit for Illumina, according to Illumina's standard instructions. An Agilent 4200 Bioanalyzer was employed to assess the concentration and size distribution of the cDNA library before sequencing on an Illumina NovaSeq 6000. High‐throughput sequencing was performed according to the manufacturer's instructions (Illumina). Raw reads were filtered using seqtk before being mapped to the genome with HISAT2 (version 2.0.4). Gene fragments were counted using StringTie (version 1.3.3b), followed by TMM (trimmed mean of M‐values) normalization. Differential expression analysis was conducted using the edgeR package. Significantly differentially expressed genes (DEGs) were defined as those with a false discovery rate (FDR) *q*‐value less than 0.05 and a fold‐change greater than 2. KEGG (Kyoto Encyclopedia of Genes and Genomes) pathway enrichment analysis was performed using the appropriate R package.

### Quantitative Reverse Transcription PCR (qRT‐PCR)

qRT‐PCR was performed as previously described. Briefly, total RNA was extracted from cells using Trizol Reagent (Invitrogen, Carlsbad, CA, USA) according to the manufacturer's protocol. The RNA concentration was determined using a NanoDrop spectrophotometer (Life Technologies, Carlsbad, CA, USA). cDNA was synthesized from 1 µg of RNA using Oligo(dT) primers and Superscript III reverse transcriptase, following the manufacturer's protocol (Vazyme, China). Quantitative PCR was carried out using SYBR Premix (Vazyme, China). Data were collected using a PIKOREAL 96 real‐time PCR system (Thermo Scientific, MA, USA). All reactions were performed in triplicate. The primers used in this study are listed in the key resources table (Table S1, Supporting Information). Primers were designed using PrimerBank or OriGene (OriGene Technologies, Inc.) and verified for specificity using the NCBI Primer‐BLAST tool. The primers were synthesized by SANGON (Shanghai, China).

### Flow Cytometry Analysis (FACS)

Tumors were collected immediately after euthanizing the mice. Tumor tissues were dissociated into single‐cell suspensions using collagenase treatment. The resulting single‐cell suspension was incubated with Red Blood Cell Lysis Buffer (Beyotime Biotechnology) for 10–15 min at 4 °C to lyse red blood cells. Cells were then washed, counted using an automated cell counter (Countstar), and 1 × 10⁶ cells were blocked with FcR Blocking Reagent (BioLegend) or mouse serum. The cells were subsequently stained for cell surface markers using antibodies purchased from BioLegend. Flow cytometry analysis was performed using a CytoFLEX flow cytometer (BD Biosciences), and the data were analyzed with CytExpert software (version 2.4.0.28) and FlowJo software (Tree Star, Inc., Ashland, OR).

### Mass Cytometry (CyTOF)

Tumor tissues harvested from the mice were processed into single‐cell suspensions using the same methods as those used for flow cytometry. A total of 40 metal‐conjugated antibodies were pre‐prepared using the Maxpar Antibody Labeling Kit, following the manufacturer's instructions (Table S1, Supporting Information). The qualified cell samples were then incubated with these antibodies for surface marker staining. After washing, the cells were analyzed using the Helios3 CyTOF system (Fluidigm, USA) for signaling detection. Data acquisition and analysis were performed by PLTTech Inc. (Hangzhou, China). Briefly, after normalization with the EQ Four Element Calibration Beads, CyTOF data were analyzed on the Cytobank platform (https://www.cytobank.org/). Nonlinear t‐distributed stochastic neighbor embedding (t‐SNE) dimensionality reduction and unsupervised PhenoGraph clustering were conducted using the cytofkit package in R to identify distinct immune cell populations.

### Single‐Cell RNA Sequencing

Tumor tissues harvested from mice were processed into single‐cell suspensions using the same methods employed for flow cytometry. The single‐cell suspensions were stained with a fluorochrome‐labeled CD45 antibody for sorting and labeled with DAPI to identify live cells. CD45⁺ cells were isolated using a Beckman cell sorter.

Cell suspensions were prepared using the Shbio Tissue Dissociation Kit (21517‐10) for tissue samples. The prepared cell suspensions, barcoded gel beads, and oil were loaded into separate wells of the Chromium Chip K. Using the 10x Genomics Chromium system, Gel Beads‐in‐Emulsion (GEMs) were generated. The GEMs were then transferred to a PCR instrument for reverse transcription. The barcoded gel beads contained 30‐nucleotide oligo‐dT reverse transcription primers, enabling poly‐A RNA from the cells to be reverse‐transcribed into first‐strand cDNA tagged with barcodes and Unique Molecular Identifiers (UMIs). Following reverse transcription, magnetic beads were used to purify the cDNA. The purified cDNA was then amplified by PCR. The concentration of cDNA was measured using a Qubit fluorometer, and fragment size was assessed using an Agilent 2100 Bioanalyzer.

After cDNA amplification, the products were enzymatically fragmented and size‐selected using magnetic beads to obtain optimal fragment sizes. End repair, A‐tailing, and adapter ligation were performed to introduce the Read 2 sequencing primer. PCR was then used to construct cDNA libraries containing P5 and P7 adapters. The libraries were purified using magnetic beads. Library concentration was measured using a Qubit fluorometer, and fragment size was assessed using an Agilent 4200 Bioanalyzer. Cluster generation and hybridization of the first read sequencing primer were performed according to the Illumina User Guide. The flow cell containing the clusters was loaded onto the sequencing machine. Paired‐end sequencing was performed, controlled by the data collection software provided by Illumina, with real‐time data analysis during the sequencing process.

### Single‐Cell RNA Sequencing Data Analysis

Data Processing and Initial Analysis: Raw single‐cell RNA sequencing (scRNA‐seq) data were processed using CellRanger software (10× Genomics)^[^ ^46^ ^]^ to perform alignment, gene quantification, and cell identification, converting FASTQ files into cell expression matrices. Based on the cell types and quantification results identified by CellRanger, the Seurat package^[^ ^47^ ^]^ was used for secondary filtering and analysis of the data, including subcluster analysis, Gene Ontology (GO) and Kyoto Encyclopedia of Genes and Genomes (KEGG) enrichment analysis, and cell–cell communication analysis.

Quality Control and Filtering: For each sample, a tailored scRNA‐seq data analysis pipeline was used to cluster detected cells and identify cell types, enabling comparisons of cell composition and abundance across different samples. Data preprocessing and normalization can affect subsequent cell classification results; therefore, different datasets require specific filtering methods to avoid inaccuracies due to excessive abnormal cells or genes. Considering batch differences caused by experimental or sequencing factors (such as sequencing depth, UMI counts, gene expression levels, mitochondrial, and ribosomal content), applying the same filtering thresholds across all samples is unreasonable. The distributions of UMI counts, gene counts, mitochondrial gene percentages, and ribosomal gene percentages were statistically analyzed for each sample to calculate appropriate filtering thresholds. Cells with high mitochondrial gene content indicate potential cell death; thus, an upper mitochondrial gene percentage threshold was set.^[^ ^48^ ^]^ Cells with erythrocyte gene percentages exceeding 1% were filtered out. If the mitochondrial gene percentage filtering threshold for an individual sample exceeded 25%, it was re‐filtered using a threshold of 25%. As scRNA‐seq typically encapsulates cells into droplets, with each droplet ideally containing a single cell associated with a barcode, increasing cell loading can lead to a higher probability of droplets containing two or more cells (doublets or multiplets). These doublets shared the same barcode and were treated as single cells, potentially introducing erroneous information into subsequent analyses. Quality control based on total reads per cell, genes per cell, and mitochondrial gene percentage could filter out most doublets; however, some might remain. Therefore, the Scrublet algorithm^[^ ^49^ ^]^ was used to further filter doublets.

Doublet Detection Using Scrublet: The Scrublet algorithm simulated doublets by randomly pairing barcodes and adding them to the original expression matrix. All cells (including simulated doublets) were then clustered, and each cell was assigned a doublet score based on clustering results. The doublet score was proportional to the number of associated simulated doublets; higher scores indicated a higher likelihood of being a true doublet.^[^ ^49^ ^]^

Cell Cycle Analysis: Cell cycle scores were calculated for each cell based on the expression of cell cycle genes,^[^ ^50^ ^]^ determining the cell cycle stage of each cell. Principal component analysis (PCA) was used to visualize and evaluate cell cycle effects. If the effect was significant, linear regression was employed to remove cell cycle effects. In this experiment, cell cycle effects were minimal and no adjustment was made.

Highly Variable Gene Identification and Clustering: The top 2000 variable genes were identified for each sample based on the mean expression and dispersion (variance/mean) for subsequent integration analysis.^[^ ^51^ ^]^ Specifically, genes were grouped into 20 bins based on average expression. The normalized dispersion for each gene was calculated as the absolute difference between the variance and the median variance of genes within the same bin. Integrated data were subjected to PCA for dimensionality reduction. The Louvain algorithm, also known as graph‐based clustering, was applied to the normalized data for clustering analysis. This algorithm is widely used in scRNA‐seq data analysis published in high‐impact journals and is effective in identifying hierarchical community structures by optimizing the modularity of the community network.

Dimensionality Reduction and Visualization: Uniform Manifold Approximation and Projection (UMAP), a manifold learning technique for dimensionality reduction, was used for data visualization. UMAP offered visualization quality comparable to t‐SNE, retains more global structure, and had excellent runtime performance, making it suitable as a general‐purpose dimensionality reduction technique for machine learning.

Differential Expression and Functional Enrichment Analysis: Clustering analysis divided the cells into distinct clusters. Based on sample grouping information, inter‐group differential analysis was performed for each cluster to identify differentially expressed genes (DEGs). DEGs were screened based on fold change (FC > 1.5 or FC < 0.66) and *p*‐value < 0.05. GO and KEGG enrichment analyses were conducted on the DEGs to identify significantly enriched functional pathways, aiming to explore functional heterogeneity among sample groups.

Marker Gene Identification and Cluster Characterization: Marker genes were analyzed for all clusters using the Wilcoxon algorithm, employing a group one vs rest approach to score marker genes. Genes that were specifically highly expressed (logFC > 0.25) in each cluster and expressed in at least 20% of cells were selected as significant marker genes. While marker genes represent the characteristic genes expressed by each cluster, individual genes may not fully reflect the cluster's properties. Therefore, GO and KEGG enrichment analyses were performed on the marker genes of each cluster to further infer the cellular functional characteristics through the pathways and GO terms associated with the marker genes.

Cell Type Annotation: Cell types were annotated using SingleR^[^ ^52^ ^]^ and marker genes. SingleR selects RNA‐seq data as a reference, identifies genes with high variability between different cell types in the reference database, and calculates the correlation between the predicted cells and the reference database. By iteratively removing the cell type with the lowest correlation, SingleR ultimately annotates the predicted cell types.

Additionally, based on literature, marker gene lists related to all expected cell types were collected. SCINA (Semi‐supervised Category Identification and Assignment)^[^ ^53^ ^]^ assigned cell type labels to individual cells based on the expression levels of marker genes. SCINA is an automated algorithm for cell type detection and assignment in scRNA‐seq data, capable of assigning cell identities to all cells based on prior knowledge of features (marker genes) highly (or lowly) expressed in each cell group.

Based on the marker genes provided in SCINA and the semi‐supervised annotation results, various visualization methods (heatmaps, bubble plots, UMAP/t‐SNE plots, violin plots) were used to display the expression characteristics of marker genes in various cell groups.

Cell–Cell Communication Analysis: Cell communication occurs throughout organisms, forming complex regulatory networks. Signal interactions mainly include adjacency‐type and diffusion‐type transmission methods. Adjacency‐type transmission includes nerve signal transmission and antigen presentation, characterized by signal transmission between two closely associated cells, such as direct binding of membrane‐bound ligand‐receptor pairs. Diffusion‐type transmission primarily involves secreted proteins diffusing through the extracellular matrix or body fluids to other cells, with endocrine, paracrine, and autocrine being typical examples.

The CellChat^[^ ^54^ ^]^ analysis method was used to infer, visualize, and analyze cell–cell communication networks. CellChat models communication probabilities and identifies important communication networks by subdividing detected ligand–receptor pairs into signaling pathways. CellChat constructed a cell communication reference database‐CellChatDB‐by selecting 2021 validated cell communication relationships (applicable to humans and mice), considering multi‐subunit receptors and other important signaling auxiliary factors, including soluble agonists, antagonists, co‐stimulatory, and co‐inhibitory membrane‐bound receptors.

In Vitro Cytotoxicity Assay: Approximately 3000 cells per well were seeded in 96‐well plates 24 h before the addition of BP‐PEG. The cells were then treated with varying concentrations of BP‐PEG (0–50 µg mL^−1^) for 24–72 h. Cell viability was assessed using the Cell Counting Kit‐8 (CCK‐8) assay, following the manufacturer's instructions. All samples were analyzed in triplicate, and data are presented as the mean ± standard deviation from two to three independent experiments.

### Co‐Culture Assay

Peripheral blood samples were collected from breast cancer patients prior to surgery, in accordance with the experimental guidelines of the University of Science and Technology of China (2022KY256). To establish an in vitro co‐culture system, PBMCs were isolated from the blood samples using Ficoll–Paque density gradient centrifugation. The isolated PBMCs were then co‐cultured with MDA‐MB‐231 cells at a 10:1 ratio for 48 h, with or without BP‐PEG. Viable MDA‐MB‐231 cells were captured and counted before processing for crystal violet staining. The PBMC‐containing suspension was also sent for flow cytometry analysis.

For the co‐culture of neutrophils with 4T1 cells, single cells from tumors were isolated 48 h after BP‐PEG treatment, as described for flow cytometry. Neutrophils were isolated from the resulting cell suspension using magnetic selection with anti‐Ly‐6G microbeads (Miltenyi Biotec). Isolated neutrophils were co‐cultured with 4T1‐H2B‐GFP tumor cells at a 40:1 neutrophil‐to‐cancer cell ratio. GFP^+^ tumor cells were quantified 24 h later by microscopy.

### Histology and Immunohistochemistry (IHC)

Tissue specimens from mice were fixed in 10% buffered formalin for 24 h and then stored in 70% ethanol until paraffin embedding. Sections of 5 µm were cut and stained with hematoxylin and eosin (H&E) or used for immunohistochemical analysis. Immunohistochemistry was performed on formalin‐fixed, paraffin‐embedded tumor tissue sections using the biotin‐avidin method, as previously described. Sections were stained with antibodies against Ly6G (neutrophil marker) and granzyme B (GZMB). The immunoreactivity was developed using DAB, followed by counterstaining with hematoxylin. Images were captured using the 3DHISTECH Pannoramic MiDi slide scanner equipped with a 20× objective lens.

### Enzyme‐Linked Immunosorbent Assay (ELISA)

Cells were treated according to their respective groups. Subsequently, the culture medium from each group was collected. After centrifugation, the supernatant was analyzed for CXCL2 concentrations using an ELISA assay (Lianke Biotechnology, Hangzhou, China). Following the addition of the stop solution, absorbance was measured at 450 and 630 nm using a microplate reader.

### Drawing Schematic Diagram

All schematic diagrams in this work were created in BioRender. Yu, F. (2024), Agreement number: *KP27PKUH9C*). Including images in Figures 1, 2, 3, 5, 6, 7, and 8, and S1H and S7D (Supporting Information).

### Statistical Analysis

The quantification of data analyses was mainly performed with GraphPad Prism statistical software, except bulk RNAseq and scRNAseq. The detailed information on the processing of bulk RNA‐seq and scRNA‐seq is described in the relevant sections. For in vivo assay, at least five mice were used for each group, and at least three samples or independent experiments were performed for other experiments. *p*‐Value was determined by unpaired *t*‐tests. A value of *p* < 0.05 was considered statistically significant.

## Conflict of Interest

The authors declare no conflict of interest.

## Author Contributions

J.W. conducted the experiments and supported data acquisition. W.Q.Y. performed the analysis of all sequencing data. Y.X.S., H.J.Z., and B.Y.Z. prepared and characterized the various forms of Black Phosphorus used in this study. H.S. assisted to data analysis and interpretation. X.P. assisted to ELISA assay. Z.Y.Y., M.X.P., and Y.H. supervised manuscript revisions. F.Z.Y. conceptualized the project, conducted experiments, analyzed and interpreted the data, and authored the manuscript. Z.Y.Y., F.Z.Y., provided project oversight and supervised manuscript revisions. All authors reviewed and approved the final version of the manuscript for submission.

## Supporting information



Supporting Information

## Data Availability

The data supporting the findings of this study are available from the main corresponding author Fazhi Yu upon reasonable request.
