# Supplementary material for: Therapeutic Black Phosphorus Nanosheets Elicit Neutrophil Response for Enhanced Tumor Suppression
Source: Adv Sci (Weinh). 2025 Jan 22;12(14):2414779. doi: 10.1002/advs.202414779 (PMC11984869; doi:10.1002/advs.202414779)
Supplement: Supplementary file 1 — Supporting Information [file ADVS-12-2414779-s001.docx]

**Therapeutic** **Black Phosphorus Nanosheets Elicit Neutrophil Response for Enhanced Tumor Suppression**

*Jing Wang, Weiqiang Yu, Hui Shen, Yanxiang Sang, Hongjie* *Zhang, Benyan Zheng, Xue Peng, Yuan Hu*, Xiaopeng Ma*, Zhenye Yang*,* *Fazhi Yu****

*J. Wang*, *X. Ma, F. Yu*

Department of General Surgery, The First Affiliated Hospital of University of Science and Technology of China, Division of Life Sciences and Medicine, University of Science and Technology of China, Hefei, Anhui, 230036, P. R. China

E-mail: [XiaopengMa@fsyy.ustc.edu.cn](mailto:XiaopengMa@fsyy.ustc.edu.cn); [fzy1988@ustc.edu.cn](mailto:fzy1988@ustc.edu.cn);

*Z. Yang*

Department of Digestive Disease, The First Affiliated Hospital of University of Science and Technology of China, Division of Life Sciences and Medicine, University of Science and Technology of China, Hefei, Anhui, 230036, P. R. China

E-mail: [zhenye@ustc.edu.cn](mailto:zhenye@ustc.edu.cn);

*Z. Yang, F. Yu*

Key Laboratory of Immune Response and Immunotherapy, School of Basic Medical Sciences, Division of Life Sciences and Medicine, University of Science and Technology of China, Hefei, China

*Y. San, H. Zhang, B. Zheng, Y. Hu*

State Key Laboratory of Fire Science, University of Science and Technology of China, Hefei, Anhui, 230036, P. R. China

E-mail: [yuanhu@ustc.edu.cn](mailto:yuanhu@ustc.edu.cn)

*W. Yu*

HIM-BGI Omics Center, Hangzhou Institute of Medicine (HIM), Chinese Academy of Sciences (CAS), Hangzhou, China

*H. Shen*

School of Life Science and Technology, China Pharmaceutical University

*X. Peng*

Department of General Surgery, The Chinese People’s Armed Police Forces Anhui Provincial Corps Hospital, Hefei 234000, Anhui Province, China

Keywords: black phosphorus, cancer therapy, immunomodulatory, neutrophil

**Abstract**

Black phosphorus (BP) has demonstrated potential as a drug carrier and photothermal agent in cancer therapy; however, its intrinsic functions in cancer treatment remain underexplored. In this study, we investigate the immunomodulatory effects of polyethylene glycol-functionalized BP (BP-PEG) nanosheets in breast cancer models. Using immunocompetent mouse models-including 4T1 orthotopic BALB/c mice and MMTV-PyMT transgenic mice-we find that BP-PEG significantly inhibits tumor growth and metastasis without directly inducing cytotoxicity in tumor cells. Mass cytometry analysis reveal that BP-PEG reshapes the tumor immune microenvironment by recruiting neutrophils. Neutrophil depletion experiments further demonstrate that the anti-tumor effects of BP-PEG are dependent on neutrophils. Moreover, bulk and single-cell RNA sequencing indicate that BP-PEG is mainly taken up by macrophages, leading to the release of inflammatory factors such as IL1A and CXCL2, which enhance neutrophil recruitment and activation, thereby amplifying the anti-tumor immune response. Finally, co-culture assays confirm that BP-PEG indeed enhances the anti-tumor activity of neutrophils and natural killer (NK) cells. These findings position BP-PEG as an immunomodulatory agent capable of reprogramming the tumor microenvironment to promote innate immunity against breast cancer. By stimulating neutrophil-mediated anti-tumor activity, BP-PEG offers a unique therapeutic approach that could potentially enhance the efficacy of existing cancer immunotherapies.

1. Introduction

Cancer remains a leading cause of mortality worldwide, necessitating innovative therapeutic strategies[1]. Two-dimensional (2D) nanomaterials have gained significant attention in oncology due to their unique properties and multifunctional applications[2, 3]. Among them, black phosphorus (BP) has emerged as a promising candidate for cancer therapy owing to its layered structure, tunable electronic properties, and biodegradability[4-6]. BP consists of corrugated planes of phosphorus atoms connected by strong intralayer P–P bonds and weak interlayer van der Waals forces, allowing exfoliation into few-layer or monolayer nanosheets[7]. This exfoliation results in a layer-dependent bandgap ranging from 0.3 eV (bulk BP) to approximately 2 eV (monolayer BP), endowing the material with remarkable optical and electronic properties suitable for biomedical applications. Notably, BP degrades into non-toxic phosphate ions under physiological conditions[8]. Its responsiveness to pH and near-infrared (NIR) radiation enables controlled drug release and reactive oxygen species (ROS) generation, making it attractive for photothermal and photodynamic therapies[9, 10].

Despite these attributes, most studies have focused on BP's role as a drug carrier or photothermal agent[9], with limited exploration of its direct impact on the tumor microenvironment (TME) and the immune system. The TME is a complex milieu of tumor cells, stromal cells, immune cells, and extracellular matrix components that contribute to tumor progression and therapy resistance[11, 12]. Immune cells within the TME can suppress or promote tumor growth depending on their activation states[13-15]. Advancements in cancer immunotherapy have highlighted the potential of modulating the immune system to achieve durable anti-tumor responses[16]. Nanomaterials have been investigated as immunomodulatory agents capable of reprogramming the TME to overcome immunosuppression[17-19]. Certain nanomaterials act as adjuvants, enhancing antigen presentation and stimulating immune cell activation[20, 21]. However, BP's immunomodulatory effects, particularly on innate immune cells like neutrophils and macrophages, remain underexplored.

Neutrophils, the most abundant leukocytes in human blood, play critical roles in innate immunity and have been implicated in cancer progression[22] . While traditionally considered tumor-promoting, emerging evidence suggests that neutrophils can exhibit anti-tumor functions under specific conditions[23]. Polarizing neutrophils toward an anti-tumor phenotype represents a novel therapeutic avenue[24, 25]. Given BP's potential interactions with immune cells upon administration[26], understanding its effects on the TME's immune components is crucial. BP's degradation products and surface properties may influence immune cell behavior, leading to immune stimulation. Functionalization with biocompatible polymers like polyethylene glycol (PEG) can enhance BP's stability and modulate its biological interactions[27].

In this study, we investigated BP's direct immunomodulatory effects in a breast cancer model. We synthesized stable BP-PEG nanosheets and evaluated their impact on tumor growth and metastasis in immunocompetent mouse models. We focused on how BP-PEG influences the TME, particularly neutrophil recruitment and activation, and whether these effects contribute to its anti-tumor activity. Using in vivo experiments, including neutrophil depletion and immunodeficient mouse models, as well as advanced analytical techniques like mass cytometry and single-cell RNA sequencing, we provide insights into BP-PEG's immunomodulatory functions. Our findings suggest that BP-PEG can reshape the tumor's immune landscape, promoting innate immune responses that inhibit tumor progression.

This research enhances our understanding of BP's interactions with the immune system and highlights BP-PEG's potential as a novel immunotherapeutic agent. By harnessing innate immune responses, specifically neutrophil-mediated actions, BP-PEG offers a promising strategy that complements existing therapies and may overcome limitations of conventional chemotherapies, which often promote neutrophil infiltration and induce chemoresistance[28].

**2. Results**

**2.1. BP-PEG Exhibits Anti-Tumor Activity in immunocompetent Breast cancer mice model**

To investigate the anti-tumor mechanism of black phosphorus (BP), we first synthesized BP and modified it with polyethylene glycol (PEG) to enhance its stability, given BP's inherent instability. Subsequently, we characterized BP-PEG. Transmission electron microscopy (TEM) images revealed typical two-dimensional sheet-like structures for both BP and BP-PEG (Figure 1A). Most BP-PEG nanosheets were less than 200 nm in size, predominantly around 100 nm, which could ensure the stability and specific tissue targeting of BP-PEG (Figure 1B). The zeta potential decreased from -27.6 mV to -18.1 mV after PEG modification (Figure 1C), indicating successful PEG conjugation and enhanced stability of BP. Energy-dispersive X-ray spectroscopy (EDS) analysis confirmed the presence of C, N, O, and P elements in BP-PEG (Supplementary Figure S1A). Fourier-transform infrared (FTIR) spectra showed characteristic peaks corresponding to P=O at 1637 cm⁻¹ and enhanced peaks at 2924 cm⁻¹ due to C–H vibrations from PEG chains (Supplementary Figure S1B). X-ray diffraction (XRD) patterns and Raman spectra confirmed the successful exfoliation of BP into multilayer nanosheets (Supplementary Figures S1C and S1D). UV-visible spectroscopy showed a positive correlation between BP concentration and absorbance (Supplementary Figure S1E). Additionally, atomic force microscopy (AFM) images showed that the average size and thickness of PEGylated BP nanosheets (BPNSs) slightly increased due to the PEG-NH2 coating on the surface (Supplementary Figure S1F). Sectional plots revealed that the thickness of BPNSs ranged from 5 to 10 nm, while BPNS-PEG thickness ranged from 10 to 20 nm (Supplementary Figure S1F). Together, these findings confirm the successful synthesis of BP-PEG nanosheets with improved physiological stability.

We then aimed to explore whether BP-PEG possesses anti-tumor activity in breast cancer, as several studies have demonstrated the promise of BP-based approaches in clinical breast cancer therapy. To this end, we injected 4T1 cells into the fat pad of immunocompetent BALB/C mice to establish a syngeneic model, which is widely used in preclinical cancer research, particularly for studying triple-negative breast cancer (TNBC), as it replicates many aspects of human TNBC (Figure 1D). Once the average tumor volume reached approximately 100 mm³, BP-PEG was administered intravenously, as outlined in the experimental design (Figure 1D). BP-PEG treatment significantly inhibited the growth of 4T1 breast tumors compared to the control group (Figure 1E), and tumor weights were also reduced (Figure 1E). Histological analysis of major organs showed no obvious toxicity (Supplementary Figure S1G).

To determine whether the observed reduction in tumor growth was due to BP alone and not PEG, we treated 4T1 tumor-bearing BALB/c mice with saline, 200 µL PEG, 10 mg/kg BP, or 10 mg/kg BP-PEG. The results indicated that PEG administration alone had no significant effect on tumor growth when compared to the saline-treated control group (Supplementary Figure S1H). In contrast, BP alone was able to reduce tumor growth, with an effect comparable to that seen with BP-PEG treatment at the same concentration (Supplementary Figure S1H). Additionally, we tested whether reducing the BP-PEG dose from 10 mg/kg to 5 mg/kg would still impact tumor growth. While 5 mg/kg BP-PEG also reduced tumor growth, its efficacy was lower than that of the higher dose (Supplementary Figure S1H). Based on these findings, we conclude that 10 mg/kg BP-PEG is more suitable for evaluating anti-tumor effects in this model. Additionally, BP-PEG treatment markedly suppressed lung metastasis of 4T1 cells (Supplementary Figure S1I).

To further confirm the inhibitory effect of BP-PEG on tumor growth in an immunocompetent breast cancer model, we treated MMTV-PyMT transgenic mice, a model that closely mimics human breast cancer progression, with BP-PEG. Similar anti-tumor effects were observed in MMTV-PyMT transgenic mice (Figures 1F and 1G). Altogether, these results indicate that BP-PEG effectively inhibits breast cancer development and metastasis in models that closely mimic human breast cancer.


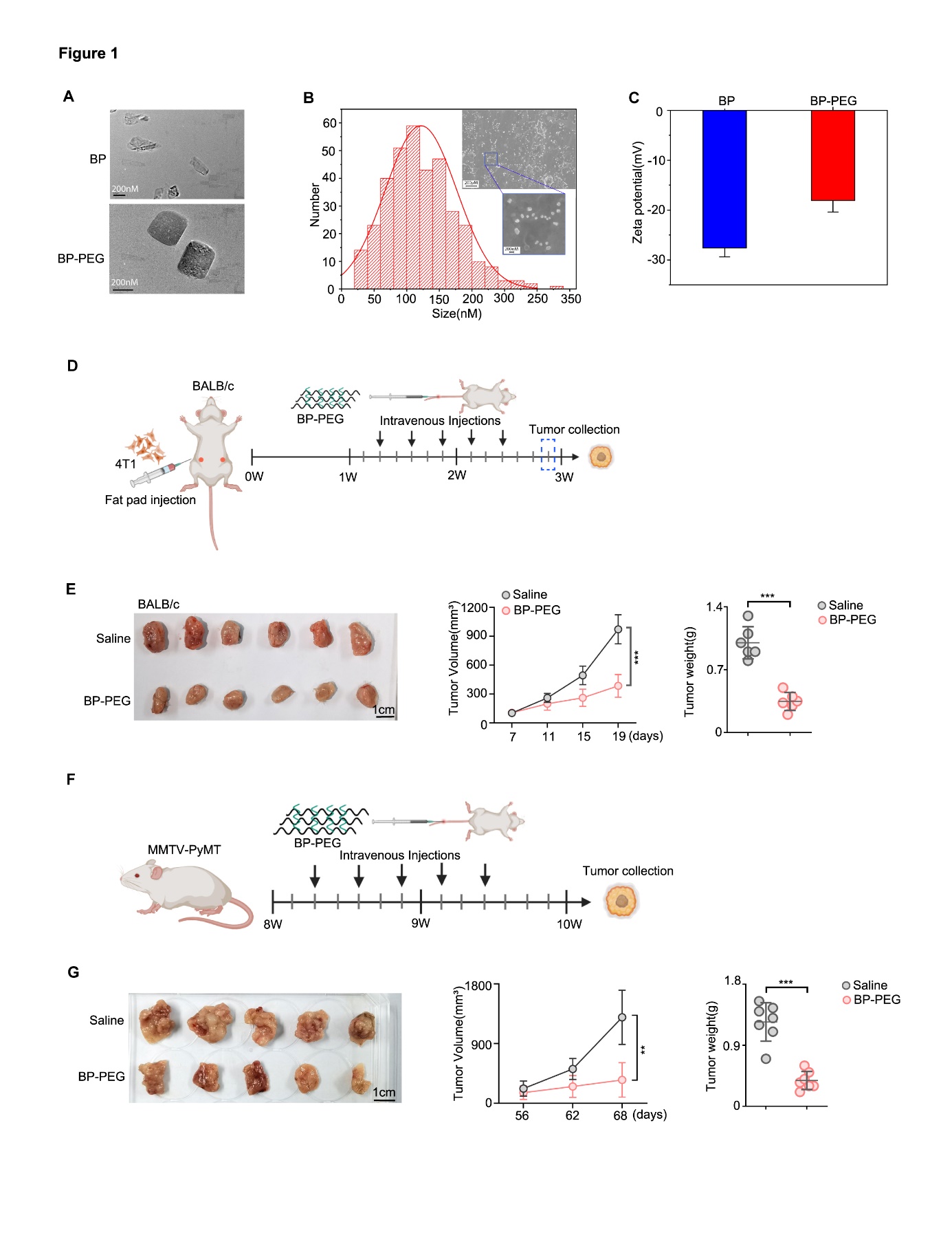


**Figure 1.** Characterization of BP-PEG nanosheets and their anti-tumor activity in an immunocompetent breast cancer mouse model. A) Transmission electron microscopy (TEM) images of black phosphorus (BP) and polyethylene glycol-modified black phosphorus (BP-PEG) nanosheets, Scale bar: 200nm. B) SEM image and particle size distribution of BP-PEG, Scale bar, 200μm and 200nm respectively. C) Surface charge of BP and BP-PEG. D) Schematic diagram of the experimental design. 4T1 breast cancer cells were injected into the mammary fat pad of immunocompetent BALB/c mice to establish a syngeneic tumor model. E) BP-PEG treatment significantly inhibits tumor growth in 4T1 tumor-bearing BALB/c mice. Left: Tumor growth curves showing tumor volume over time for BP-PEG-treated and control groups (n=6 per group). Right: Final tumor weights at the end of the study. F) Schematic diagram of the experimental design. BP-PEG treatment in MMTV-PyMT transgenic mice, a model that closely mimics human breast cancer progression. G) Tumor growth and weights of MMTV-PyMT transgenic mice after BP-PEG treatment (n=5 per group). Data are presented as mean ± SD; p values were determined by two-tailed unpaired t test; **p < 0.01, ***p < 0.001.

**2.2. BP-PEG anti-tumor growth dependent on the Immune Microenvironment**

To investigate the anti-tumor mechanism of BP-PEG, we first examined whether BP-PEG exerts its function by directly killing tumor cells. We constructed FITC-labeled BP-PEG and confirmed that BP-PEG primarily accumulated in 4T1-derived tumor tissues in vivo using an animal imaging system (Supplementary Figure S2A). Electron microscopy also confirmed that BP-PEG could be taken up by 4T1 tumor cells in vitro (Supplementary Figure S2B).

Next, we used flow cytometry to quantify the amount of BP-PEG internalized by tumor cells in vivo and compared it to the uptake observed in vitro (Figure 2A). By dissociating tumor tissue to obtain single cells and labeling tumor cells with the tumor marker EPCAM, we measured the amount of BP-PEG internalized by tumor cells in vivo 24 hours after BP-PEG injection via tail vein (Figure 2B). We then compared the intensity of FITC internalized by tumor cells in vivo with that of 4T1 cells treated with varying concentrations of FITC-BP-PEG in vitro (Figure 2C). The results showed that the majority of tumor cells in vivo had similar FITC intensity as 4T1 cells treated with 5 µg/ml FITC-BP-PEG in vitro (Figure 2C). This data demonstrated that, when treated with 10 mg/kg FITC-BP-PEG, tumor cells in vivo internalized an amount of BP-PEG comparable to 4T1 cells treated with 5 µg/ml FITC-BP-PEG in vitro. We found that, at this concentration, BP-PEG did not significantly affect tumor cell proliferation or survival over 72 hours (Figure 2D), suggesting that BP-PEG does not exert direct cytotoxic effects on tumor cells at physiologically relevant concentrations.

Since BP-PEG does not directly kill tumor cells, we propose that it may inhibit tumor growth in vivo by modulating the tumor microenvironment, particularly the immune environment. To explore whether BP-PEG exerts its function through the immune system, we utilized immunodeficient NOD scid gamma (NSG) mice bearing 4T1 tumors to evaluate the effects of BP-PEG (Figure 2E). The experimental procedures were consistent with those performed in BALB/c mice (Figure 1D). We found that BP-PEG treatment failed to inhibit tumor growth and metastasis in immunodeficient NSG mice bearing 4T1 tumors (Figure 2E and Supplementary Figure S2C), indicating that an intact immune system is required for BP-PEG's anti-tumor activity.

To further demonstrate that BP-PEG exerts its anti-tumor effects primarily through the immune system in the tumor microenvironment, we performed a co-culture assay. We collected blood from breast cancer patients, isolated peripheral blood mononuclear cells (PBMCs), and co-cultured MDA-MB-231 cells with PBMCs in the presence of BP-PEG or saline. Flow cytometry was used to analyze the PBMC cell components, revealing a composition similar to that of immune cells found within the tumor microenvironment (Supplementary Figure S2D). We then quantified the number of remaining tumor cells after co-culture using microscopy and violet staining (Figure 2F). The data showed that only minimal tumor cell loss occurred in the BP-PEG or PBMC treatment alone groups, while more than 90% of tumor cells were eliminated when BP-PEG and PBMCs were combined (Figure 2G-H). These findings further suggest that BP-PEG modulates the immune microenvironment to exert its anti-tumor effects.


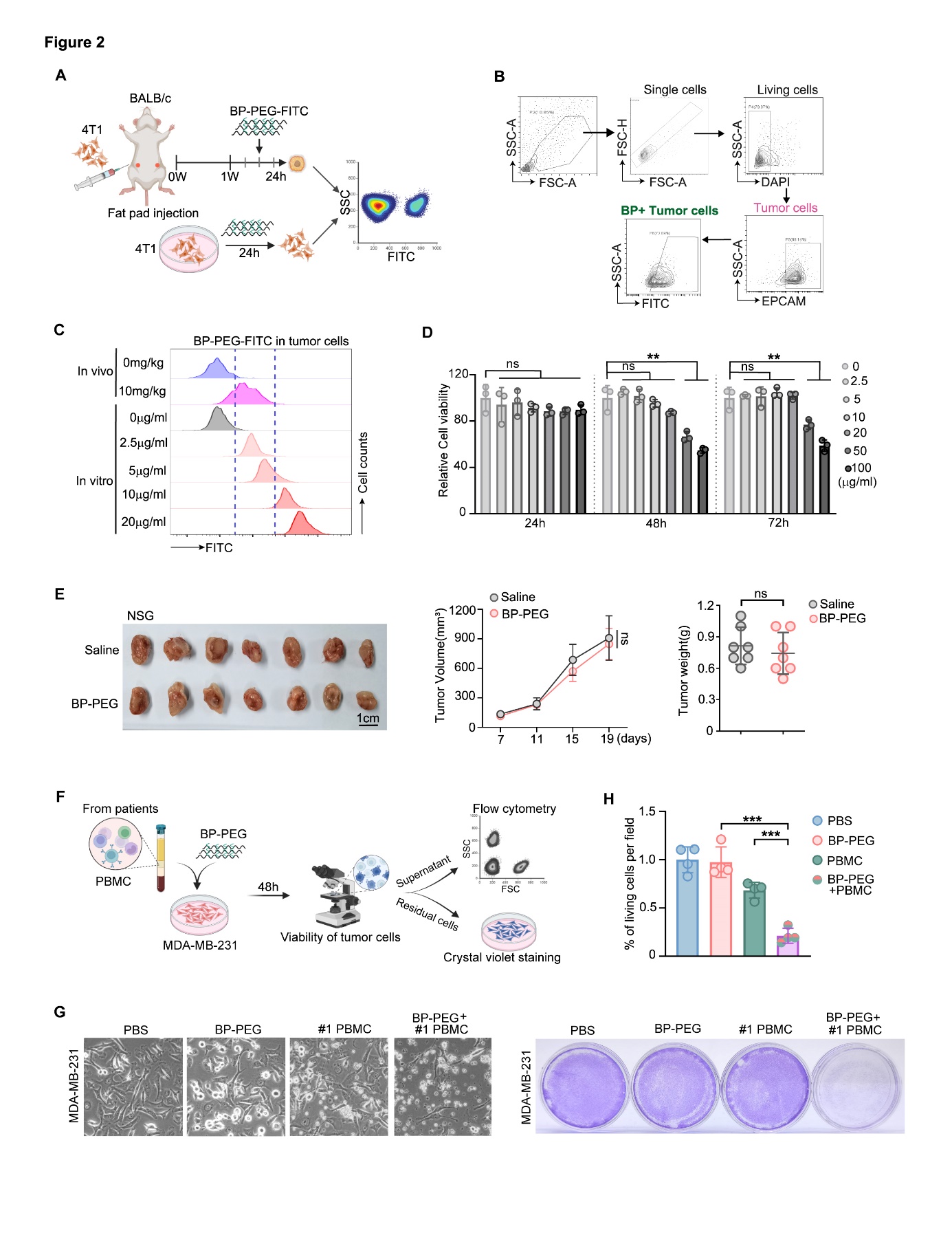


**Figure 2.** BP-PEG does not directly kill tumor cells but requires an intact immune system for anti-tumor activity. A) Schematic diagram illustrating the comparison of BP-PEG internalization by tumor cells in vivo and in vitro using flow cytometry. B) Flow cytometry gating strategy to identify EPCAM⁺ tumor cells from dissociated tumor tissues and measure FITC-BP-PEG internalization 24 hours post-injection. C) Comparison of FITC fluorescence intensity in tumor cells treated in vivo with BP-PEG (10 mg/kg) and 4T1 cells treated in vitro with varying concentrations of FITC-BP-PEG (0, 2.5, 5, 10 µg/mL). The in vivo internalization corresponds to approximately 5 µg/mL in vitro treatment. D) Cell viability assay of 4T1 cells treated with BP-PEG at concentrations up to 100 µg/mL for 72 hours, showing no significant cytotoxicity. Data are presented as mean ± SD, p values were determined by two-tailed student’s t test (ns, not significant; **p < 0.01). E) Tumor growth in immunodeficient NSG mice bearing 4T1 tumors. Left: Tumor growth curves over time (n=7 per group). Right: Final tumor weights. Data are presented as mean ± SD, ns, not significant. F) Schematic of the co-culture assay where human MDA-MB-231 breast cancer cells were co-cultured with human PBMCs in the presence or absence of BP-PEG. G) Representative images of tumor cells after co-culture and stained with crystal violet. H) Quantification of tumor cell viability after co-culture. Data are presented as mean ± SD; p values were determined by two-tailed unpaired t test; ***p < 0.001.

**2.3. BP-PEG specifically activates tumor immune microenvironments reshaping pathway in immunocompetent mice**

Then, we wanted to understand how BP-PEG exerts anti-tumor effects in the presence of an intact immune system. To identify the pathways or genes specifically regulated under BP-PEG treatment, we collected tumor tissues from both BALB/c and NSG mice and performed bulk tissue RNA sequencing for differentially expressed genes (DEGs) analysis (Figure 3A). Our results demonstrated that BP-PEG altered the gene expression profiles of tumors in both mouse models (Supplementary Figure S3A and S3B). Interestingly, we found that the DEGs in NSG mice tumors overlapped with those in BALB/c mice tumors by only 17 genes, despite the total number of DEGs exceeding 200. This finding suggests that the unique transcription profile in BALB/c mice may be responsible for the anti-tumor effect of BP-PEG. Specifically, we identified 234 genes that were differentially expressed only in BALB/c mice (Figure 3B). To understand which pathways are primarily involved in the regulation of BP-PEG's effects, we conducted pathway enrichment analysis (Figure 3C). The results indicated a significant upregulation of immune-related pathways, particularly cytokine-cytokine receptor interactions (Figure 3C), which aligns with the notion that BP-PEG's anti-tumor function depends on an intact immune system.

Next, we sought to determine which genes were involved in these pathways and their levels of change. Therefore, we conducted a heatmap analysis of the DEGs involved in immune microenvironment remodeling (Figures 3D and 3E). The analysis revealed that most of these genes were upregulated in the BP-PEG treatment group and contributed to reshape the tumor immune microenvironment, including genes such as IL1A and CXCL2 (Figures 3D and 3E). We further confirmed the upregulation or downregulation of these genes by BP-PEG using qPCR (Figure 3F). In conclusion, our data suggest that BP-PEG exerts its anti-tumor effects by specifically activating immune regulatory pathways, thereby reshaping the tumor immune microenvironment.


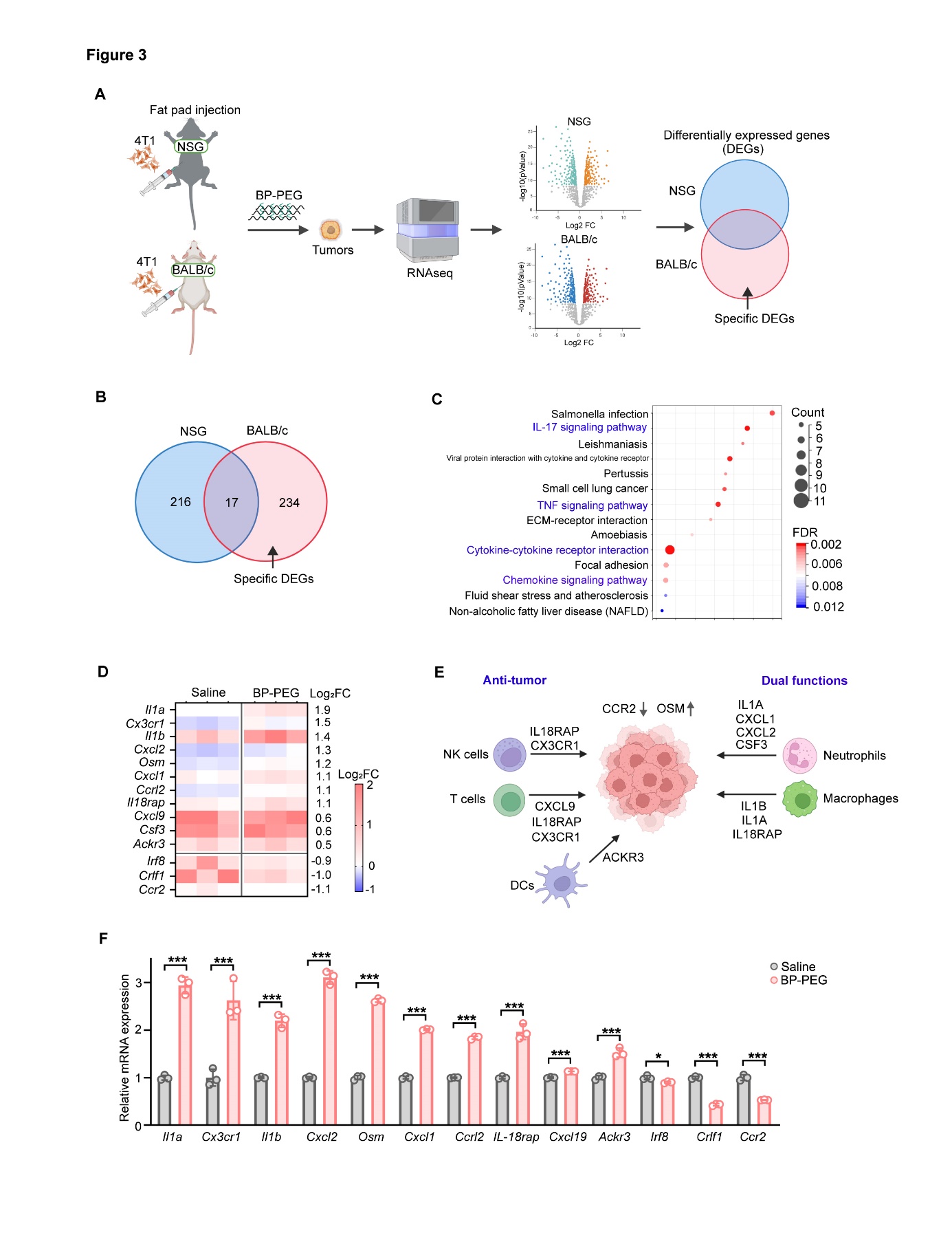


**Figure 3.** BP-PEG specifically activates immune regulatory pathways in immunocompetent mice. A) Schematic of bulk RNA sequencing performed on tumor tissues from BALB/c and NSG mice treated with BP-PEG or saline. B) Venn diagram showing the number of differentially expressed genes (DEGs) in tumors from BALB/c and NSG mice. C) Pathway enrichment analysis of the 234 DEGs uniquely regulated in BALB/c mice tumors(B), highlighting immune-related pathways such as cytokine–cytokine receptor interaction. D) Heatmap of the DEGs in BALB/c mice tumors involved in immune microenvironment remodeling after BP-PEG treatment. E) Schematic diagram illustrating the DEGs’ main functions in tumor microenvironment. F) Quantitative PCR validation of the DEGs showed in (D). Data are presented as mean ± SD; p values were determined by two-tailed unpaired t test; *p < 0.05, ***p < 0.001.

**2.4. BP-PEG Enhances Neutrophil Recruitment to the Tumor Microenvironment**

We next sought to determine whether BP-PEG treatment alters the tumor immune microenvironment. Mass cytometry, an advanced technique capable of simultaneously detecting over 40 markers, was employed to provide a comprehensive analysis of the immune cell populations within the tumor microenvironment. Tumor samples were collected from BALB/C mice, dissociated into single cells, and subjected to mass cytometry (Figure 4A). This analysis identified 26 distinct cell clusters using t-distributed stochastic neighbor embedding (t-SNE) (Figure 4A). The clusters were characterized based on marker expression (Figure 4A, Supplementary Figure S4A), and key markers were visualized in t-SNE plots (Figure 4B). We identified neutrophils, macrophages, dendritic cells, B cells, CD8^+^ T cells, CD4^+^ T cells, helper T cells, regulatory T cells (Tregs), and NK cell clusters (Figure 4A, Supplementary Figure S4A).

We next compared the composition of immune cell populations between saline and BP-PEG-treated groups (Figure 4C). The t-SNE analysis revealed that cell cluster 18, a subpopulation of neutrophils, exhibited the most significant increase in both number and percentage following BP-PEG treatment (Figures 4C-E). Post-treatment, this neutrophil subpopulation accounted for over 20% of CD45^+^ cells, emerging as the dominant cell population (Figures 4D, E). Additionally, we analyzed the proportions of other immune cell types and observed a reduction in immunosuppressive cells, specifically regulatory T cells (Tregs) and type M2 macrophages, following BP-PEG treatment, while other cell populations showed no significant change (Supplementary Figure S4B-F). Further, we assessed the antitumor activity of two key immune cell types-cytotoxic T lymphocytes (CTLs) and natural killer (NK) cells-by examining the expression of the cytotoxic marker granzyme B (GZMB). The data revealed a substantial upregulation of GZMB intensity in the predominant subpopulations of CD8^+^ T cells and NK cells (Figures 4F, G), indicating that BP-PEG treatment enhances the antitumor activity of these immune cells.

To further confirm the increase in neutrophil numbers, we performed immunohistochemical staining of tumor tissues using the neutrophil marker Ly6G (Figure 4H). Quantification of neutrophil ratios in the tissue confirmed a significant increase in both the number and percentage of neutrophils following BP-PEG treatment (Figure 4H). In addition, we assessed the anti-tumor activity of immune cells in continuous tissue sections, which had been stained for Ly6G. The results showed a significant increase in GZMB-positive cells, indicating enhanced anti-tumor functionality of immune cell after BP-PEG treatment (Figure 4H). In summary, our findings suggest that BP-PEG promotes neutrophil recruitment, contributing to the enhanced anti-tumor activity of effector cells, such as CD8^+^ T cells and NK cells.


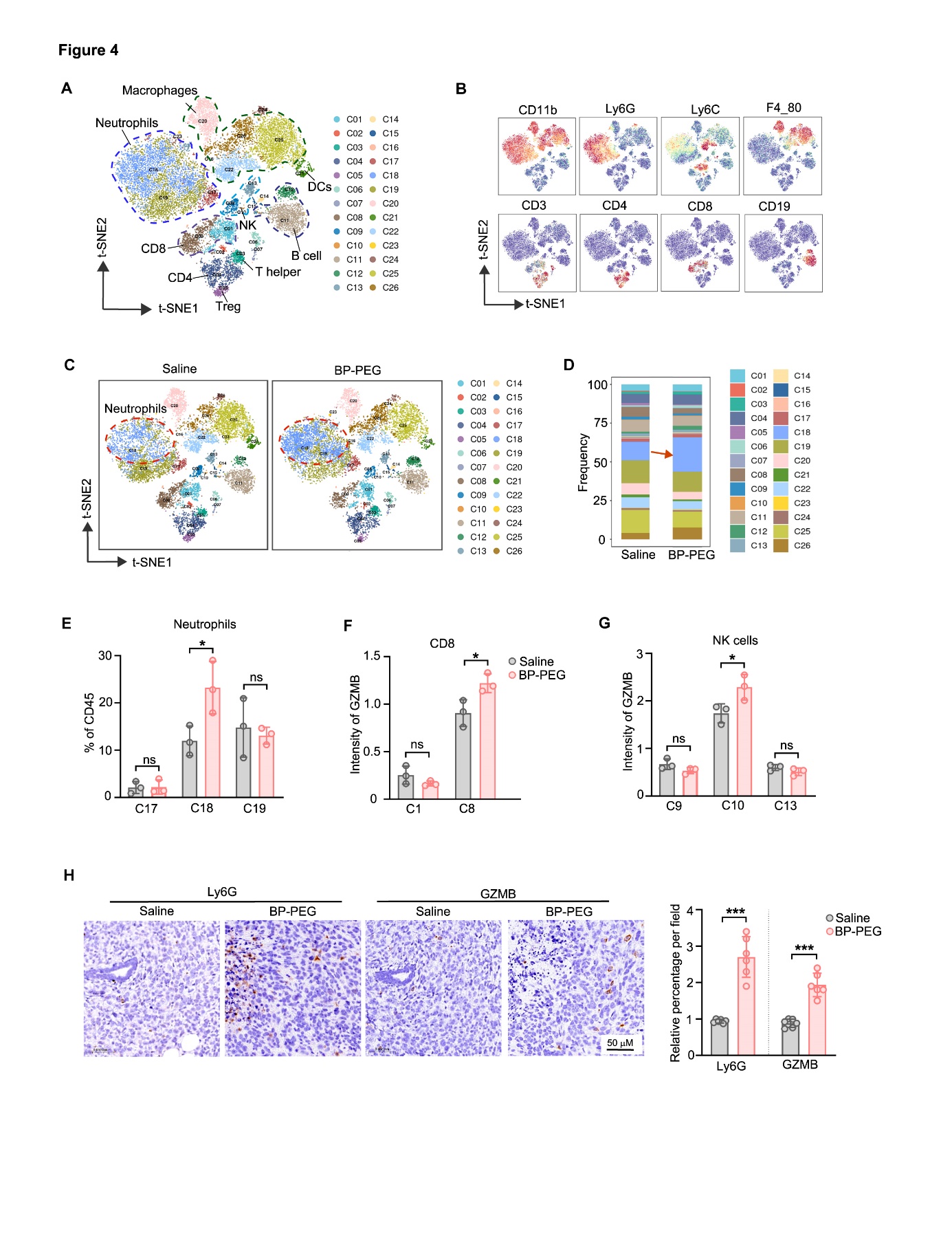


**Figure 4.** BP-PEG enhances neutrophil recruitment and activates anti-tumor immune cells. A) Mass cytometry (CyTOF) analysis of tumor-infiltrating immune cells from BALB/c mice treated with BP-PEG or saline. t-SNE plots display 26 distinct cell clusters. B) t-SNE visualization of key immune cell defined markers. C) Comparison of immune cell population distributions between saline and BP-PEG-treated groups in t-SNE plots. D) Quantification of immune cell clusters, showing a significant increase in cluster 18 (neutrophils) after BP-PEG treatment. E) Bar graph illustrating the percentage of different neutrophils clusters among CD45⁺ cells. F-G) The expression intensity of granzyme B (GZMB) in CD8⁺ T cells (F) and NK cells(G). H) Immunohistochemical staining of tumor sections for Ly6G and GZMB. Left: Representative images showing Ly6G and GZMB-positive cells; Right: Quantification of the relative percentage of Ly6G⁺ and GZMB⁺ cells per field. Data are presented as mean ± SD; p values were determined by two-tailed unpaired t test; ns, not significant; *p < 0.05, ***p < 0.001.

**2.5. Neutrophils Are Essential for BP-PEG's Anti-Tumor Activity**

Given the significant infiltration of neutrophils into tumor tissues following BP-PEG treatment, we sought to determine whether neutrophils are required for BP-PEG's anti-tumor effects. To test this hypothesis, we conducted neutrophil depletion experiments using an anti-Ly6G antibody in 4T1 tumor-bearing mice, with a schematic diagram outlining the experimental design (Figure 5A). Starting three days after 4T1 tumor cell inoculation, mice were injected daily with the anti-Ly6G antibody. Once tumors reached a volume of approximately 100 mm³, the mice were randomized into four treatment groups: (1) Saline, (2) BP-PEG, (3) anti-Ly6G, or (4) a combination of BP-PEG and anti-Ly6G (Figures 5A-B). Consistent with previous studies[29], anti-Ly6G treatment alone had minimal impact on tumor growth and weight (Figure 5B-C). While BP-PEG effectively inhibited tumor growth and reduced tumor weight, this effect was largely reversed in the mice treated with the combination of BP-PEG and anti-Ly6G (Figure 5B-C). Additionally, while BP-PEG treatment markedly suppressed lung metastasis of 4T1 cells, neutrophil depletion upregulated lung metastasis (Supplementary Figure S5A).

We confirmed the efficiency of neutrophil depletion using immunohistochemistry (IHC) staining and flow cytometry, which showed a near-complete absence of Ly6G-positive cells in the anti-Ly6G-treated group, confirming successful neutrophil depletion (Figure 5D and 5E). Furthermore, immunohistochemical staining and flow cytometry for granzyme B showed no substantial difference in the percentage of GZMB-positive cells between the control and neutrophil-depleted groups. However, the percentage of GZMB-positive cells increased with BP-PEG treatment but decreased following neutrophil depletion (Figure 5D and 5E), indicating a reduction in anti-tumor immune responses associated with neutrophil depletion. Altogether, these findings underscore the critical role of neutrophils in mediating BP-PEG-induced anti-tumor immunity.


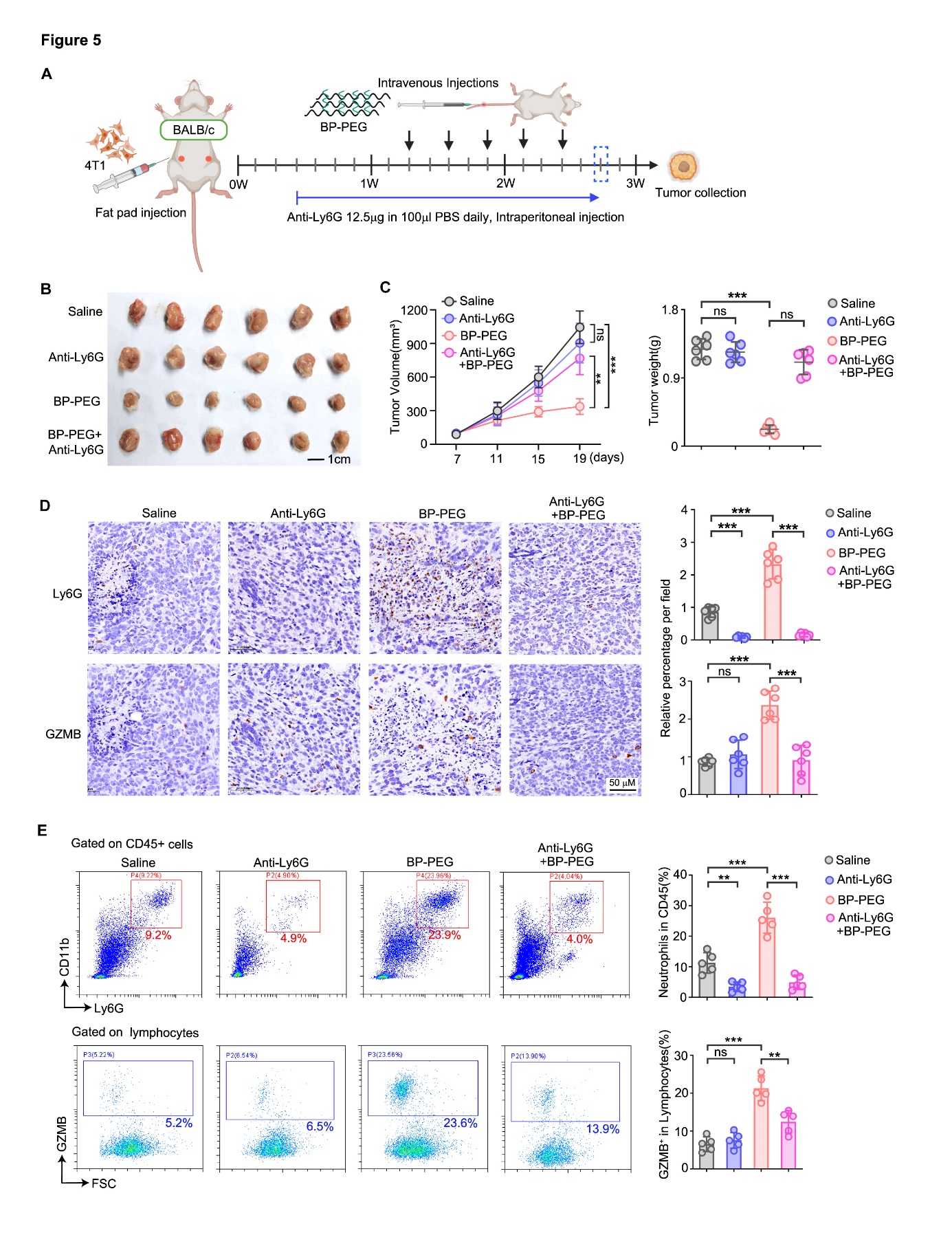


**Figure 5.** Neutrophils are essential for BP-PEG's anti-tumor effects. A) Schematic of the experimental design for neutrophil depletion using anti-Ly6G antibody in 4T1 tumor-bearing BALB/c mice. B) Representative tumors from 4T1 cells-bearing BALB/c mice treated with saline, BP-PEG, anti-Ly6G, or BP-PEG plus anti-Ly6G. C) Tumor growth curves and the tumor weights of the 4T1 tumors. D) Immunohistochemical staining for Ly6G and GZMB in tumor sections. Left: Representative images showing successful neutrophil depletion and GZMB staining, right: Quantification of the relative percentage of Ly6G⁺ and GZMB⁺ cells per fields. E) Flow cytometry was employed to analyze the percentage of neutrophils within the CD45+ cell population and GZMB+ cells among lymphocytes isolated from 4T1-derived tumors. The results were subsequently quantified. Data are presented as mean ± SD; p values were determined by two-tailed unpaired t test; ns, not significant; **p < 0.01, ***p < 0.001.

**2.6. BP-PEG Enhances Neutrophil Recruitment Through Macrophage-Mediated Inflammatory Signaling Pathways**

To further understand how BP-PEG treatment regulates neutrophil infiltration in tumor tissues, we collected tumors from BALB/c mice treated with either saline or BP-PEG. CD45-positive cells were isolated for single-cell RNA sequencing to identify immune cell populations and analyze their gene expression profiles (Figure 6A). UMAP clustering of all sequenced cells revealed a notable enrichment of several immune cell subsets-including neutrophils, macrophages, and NK cells-following BP-PEG treatment (Figure 6B, 6C, Supplementary Figure S6A). Then using the scRNA-seq data, we explored the immune cell interactions that may contribute to enhanced neutrophil infiltration and a productive anti-tumor immune response. Cell-cell interaction analysis provides insights into interactions enriched in BP-PEG-treated mice, helping to elucidate the mechanism by which BP-PEG promotes neutrophil infiltration and a productive anti-tumor immune response. Cells with high incoming interaction strength might be key responders, while those with high outgoing interaction strength could be major communicators in the system. The results showed that under saline treatment, CD8⁺ T cells and macrophage-like cells (Mph-C1) exhibited high incoming interaction strength (Figure 6D), suggesting they are primary targets or responders in the interaction network. B cells and macrophage-like cells (Mph-C2) had high outgoing interaction strength, indicating they could be major communicators in the system (Figure 6D). Under BP-PEG treatment, although CD8⁺ T cells and Mph-C1 still displayed high incoming interaction strength, Mph-C2 and neutrophils became the cells with major outgoing interaction strength (Figure 6D), indicating they emerged as significant communicators in the system, especially macrophages.

Given that macrophages have high phagocytic function, we propose that when BP-PEG is injected into mice, it is first engulfed by macrophages, which increases the inflammatory response in these cells. This response leads to the production of various cytokines that promote neutrophil infiltration and reshape the antitumor environment. To test this hypothesis, we detected the distribution of BP-PEG-FITC in tumors using flow cytometry (Supplementary Figure S6B). The results showed that macrophages indeed contained the highest level of BP-PEG-FITC among myeloid cells (Figure 6E), which is consistent with their primary role in phagocytosis. Altogether, we identified macrophages as likely initiators of interactions among immune cells in the tumor microenvironment (TME), based on the high predicted outgoing interaction strength under BP-PEG treatment.

We then analyzed functional changes in immune cells using pathway enrichment analysis. In macrophages, genes involved in neutrophil migration were upregulated under BP-PEG treatment, along with genes related to the regulation of neutrophil degranulation, innate immune response, and positive regulation of the inflammatory response (Figure 6F). These results demonstrate that BP-PEG uptake by macrophages indeed promotes inflammatory signaling pathways, which in turn promote neutrophil migration and function. In bulk tissue RNA sequencing, we found that cytokines related to neutrophils-such as IL1A, CXCL1, CXCL2, and CSF3-were upregulated in BP-PEG-treated tumors. According to scRNA-seq data, the inflammatory related pathway was also upregulated, and CXCL2 was also found to be upregulated in mixed CD45 positive cells and was predominantly expressed by macrophages and neutrophils, indicating that CXCL2 may contribute to neutrophil infiltration under BP-PEG treatment (Supplementary Figure S6C). Additionally, using ELISA to detect CXCL2 expression in THP-1 cells, we confirmed that CXCL2 is produced by macrophages and upregulated by BP-PEG (Supplementary Figure S6D).

We next analyzed the CXCL-CCR signaling pathways between macrophages and neutrophils. The results showed that CXCL2-CXCR2, CCL9-CCR1, CCL6-CCR1, and CCL3-CCR1 signaling pathways were enhanced under BP-PEG treatment (Figure 6G). Notably, the communication probability within the CXCL2-CXCR2 signaling pathway was significantly upregulated, with CXCL2 expression elevated under BP-PEG treatment. To further investigate the role of this pathway, we analyzed the expression of CXCL2 receptors, CXCR1 and CXCR2, in the tumor microenvironment using scRNA-seq data. Our analysis revealed that CXCR2 was highly expressed, predominantly on neutrophils (Supplementary Figure S6E), suggesting that CXCL2 primarily exerts its function through interaction with CXCR2 on neutrophils. To further investigate whether the CXCL2-CXCR2 signaling pathway regulates the anti-tumor effects of BP-PEG, we treated mice with the CXCR2 inhibitor SB225002 (Figure 6H). The results showed that SB225002 alone had no impact on tumor growth; however, when mice were treated with both BP-PEG and SB225002, the anti-tumor effect of BP-PEG was almost completely abolished (Figure 6H). These findings demonstrate that the anti-tumor effects of BP-PEG are primarily dependent on the CXCL2-CXCR2 signaling pathway. Altogether, our data suggest that macrophages may promote neutrophil infiltration under BP-PEG treatment through the CXCL-CCR signaling pathway, with a particularly critical role for CXCL2-CXCR2.


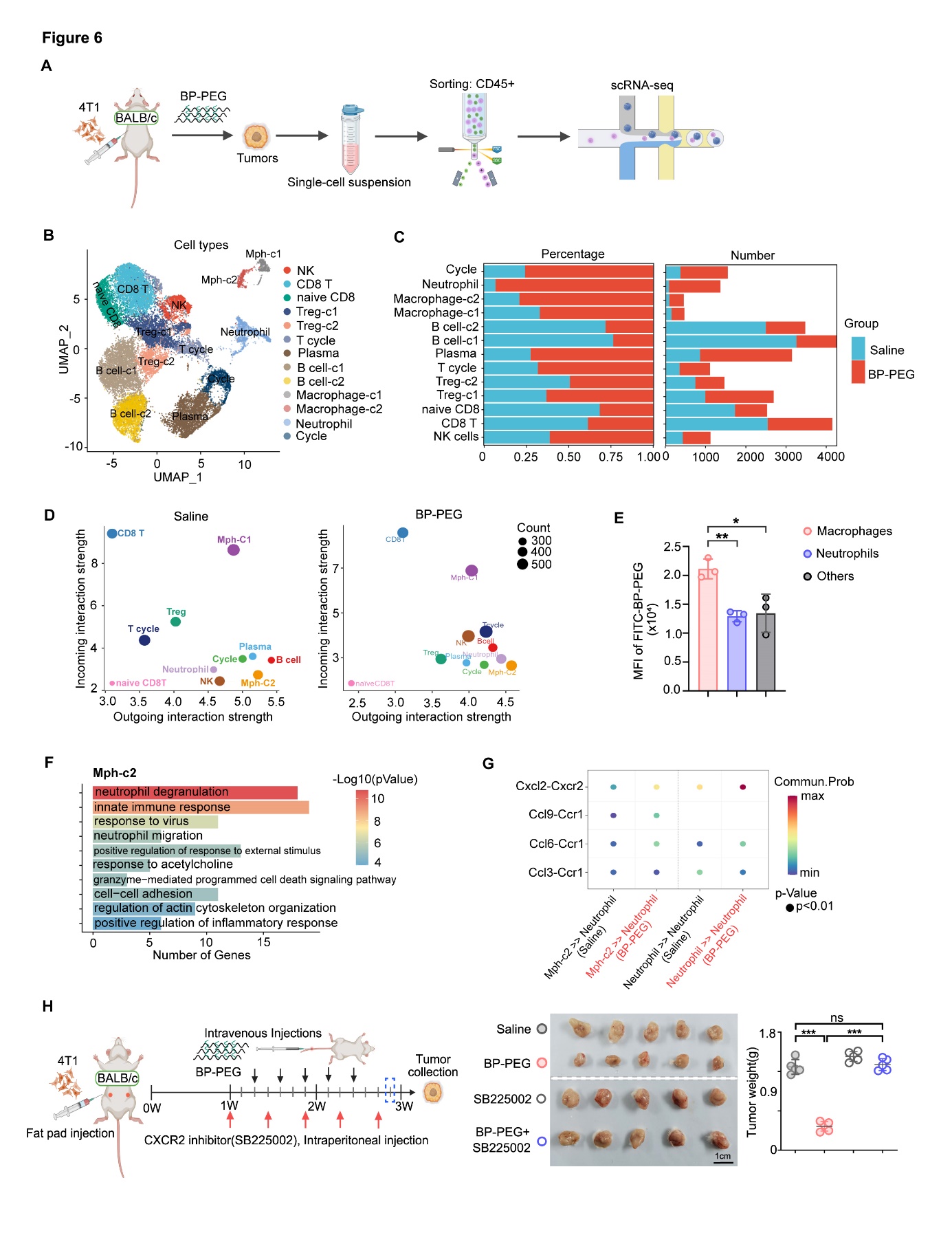


**Figure 6.** BP-PEG promotes neutrophil recruitment via macrophage-mediated inflammatory signaling. A) Workflow for single-cell RNA sequencing (scRNA-seq) of CD45⁺ immune cells isolated from tumors of BALB/c mice, comparing those treated with BP-PEG and saline. B) UMAP plots showing the clustering of immune cells based on treatment conditions. C) Bar graph quantifying the proportions of each immune cell type in tumors treated with saline versus BP-PEG. D) Analysis of cell-cell interactions, highlighting the strengths of incoming and outgoing interactions among immune cells under saline and BP-PEG treatments. E) Quantification of mean fluorescence intensity of BP-PEG-FITC in myeloid cells within tumor tissues, Data are presented as mean ± SD; *p < 0.05, **p < 0.01. F) Gene Ontology (GO) pathway enrichment analysis of macrophages, based on upregulated genes observed in BP-PEG-treated samples compared to saline-treated samples. G) Cell-cell communication networks through the CXCL-CCR signaling pathway between macrophages and neutrophils, using upregulated genes from BP-PEG treatment relative to saline treatment. H) A schematic diagram of the experimental design is provided. Saline, SB225002 (10 mg/kg), and BP-PEG (10 mg/kg) were administered to 4T1 tumor-bearing BALB/c mice in separate treatment groups (n=5 per group). Tumor images were captured, and tumor weights were analyzed at the end of the study. Data are presented as mean ± SD; p values were determined by two-tailed unpaired t test; ns, not significant; ***p < 0.001.

**2.7. BP-PEG Enhances Tumor-Killing Ability of Neutrophils and NK Cells**

We then sought to understand how neutrophils regulate anti-tumor activity. Therefore, we analyzed the differential number of interactions and interaction strengths between neutrophils and key anti-tumor immune cells, specifically CD8⁺ T cells and NK cells (Figure 7A). The data revealed that the number of interactions between neutrophils and NK cells was higher than that between neutrophils and CD8⁺ T cells or other cells (Figure 7A). Moreover, the interaction strength between neutrophils and NK cells was also notably stronger (Figure 7A). Although interactions between neutrophils and CD8⁺ T cells were also upregulated under BP-PEG treatment (Figure 7A), the potential ligand-receptor interactions between these cell types were fewer and weaker compared to those between neutrophils and NK cells (Supplementary Figure S7A). Immunofluorescence analysis further demonstrated close spatial localization of neutrophils and NK cells under BP-PEG treatment (Supplementary Figure S7B). These findings suggest that neutrophils primarily regulate the anti-tumor functions of NK cells.

We then analyzed functional changes in neutrophils and NK cells using pathway enrichment analysis. In neutrophils, pathway enrichment analysis revealed upregulation of pathways involved in the innate immune response, neutrophil degranulation, myeloid leukocyte migration, and oxidative stress and redox pathways under BP-PEG treatment (Figure 7B). These results indicate that neutrophils are involved in regulating cell migration-including their own-and that their anti-tumor function was enhanced under BP-PEG treatment, as evidenced by the upregulation of genes involved in neutrophil degranulation and oxidative stress and redox pathways. Additionally, recent studies [30] have identified three types of neutrophils in the tumor microenvironment—T1, T2, and T3—where T1 and T2 exert anti-tumor functions and T3 exhibits pro-tumor activity. To further explore this in our model, we analyzed the neutrophil subtypes and found that the gene signature profiles defining these neutrophil types were also expressed in the neutrophils observed in our study (Supplementary Figure S7C). Notably, T1 and T2 neutrophils were upregulated under BP-PEG treatment, while T3 neutrophils showed no significant change (Figure 7C, Supplementary Figure S7C). Altogether, these findings demonstrate that BP-PEG treatment can reprogram neutrophils to enhance their anti-tumor function.

Finally, we analyzed pathway enrichment in NK cells. Functions related to granzyme-mediated programmed cell death were enhanced under BP-PEG treatment, indicating that anti-tumor functions were indeed increased under BP-PEG treatment (Figure 7D). To confirm that BP-PEG could enhance the tumor-killing ability of neutrophils and NK cells, we isolated these cells from tumors treated with saline and BP-PEG using MACS and then co-cultured them with GFP-labeled 4T1 cells (Figure 7E). After 24 hours, we counted the number of GFP-positive cells under a microscope. The number of cells per field was analyzed, and the data showed that BP-PEG indeed enhanced the tumor-killing ability of neutrophils and NK cells (Figure 7F).

To further demonstrate that BP-PEG’s anti-tumor effects are dependent on NK cells in vivo, we performed an NK cell depletion assay (Supplementary Figure S7D, E). The results showed that depletion of NK cells partially reversed the anti-tumor effects of BP-PEG, as tumor weights increased in BP-PEG-treated mice lacking NK cells. However, the tumor weights in these mice remained lower than those in the saline-treated control group (Supplementary Figure S7D, E). Altogether, these findings demonstrate that BP-PEG enhances the tumor-killing abilities of NK cells and contributes to its anti-tumor efficacy.


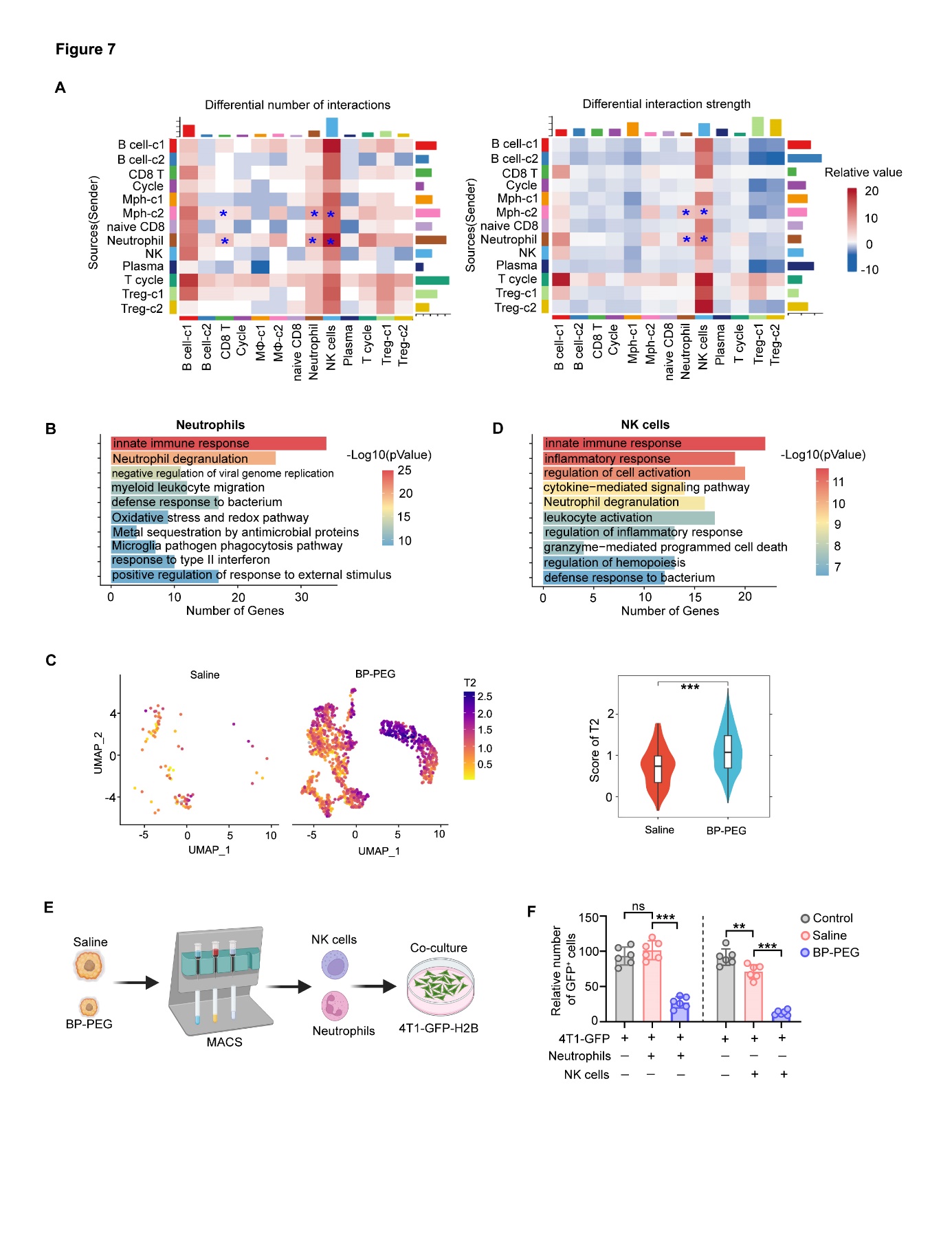


**Figure 7.** BP-PEG enhances the tumor-killing ability of neutrophils and NK Cells. A) Differential number of interactions or interaction strength, visualized as a heatmap, where red increased and blue represents decreased signaling in BP-PEG compared to saline treated tumors. The top-colored bar plot represents the sum of a column of values displayed (incoming signaling). The right- colored bar plot represents the sum of a row of values (outgoing signaling). B) Gene Ontology (GO) pathway enrichment analysis of neutrophils, based on upregulated genes observed in BP-PEG-treated samples compared to saline-treated samples. C) Analysis of the T2 gene signature expression score in neutrophils. D) Gene Ontology (GO) pathway enrichment analysis of NK cells, based on upregulated genes observed in BP-PEG-treated samples compared to saline-treated samples. E) Experimental setup for assessing the tumor-killing ability of neutrophils and NK cells isolated from tumors. Immune cells were co-cultured with GFP-labeled 4T1 cells. F) Quantification of GFP-positive tumor cells after co-culture. Data are presented as mean ± SD; p values were determined by two-tailed unpaired t test; ns, not significant; **p < 0.01, ***p < 0.001.


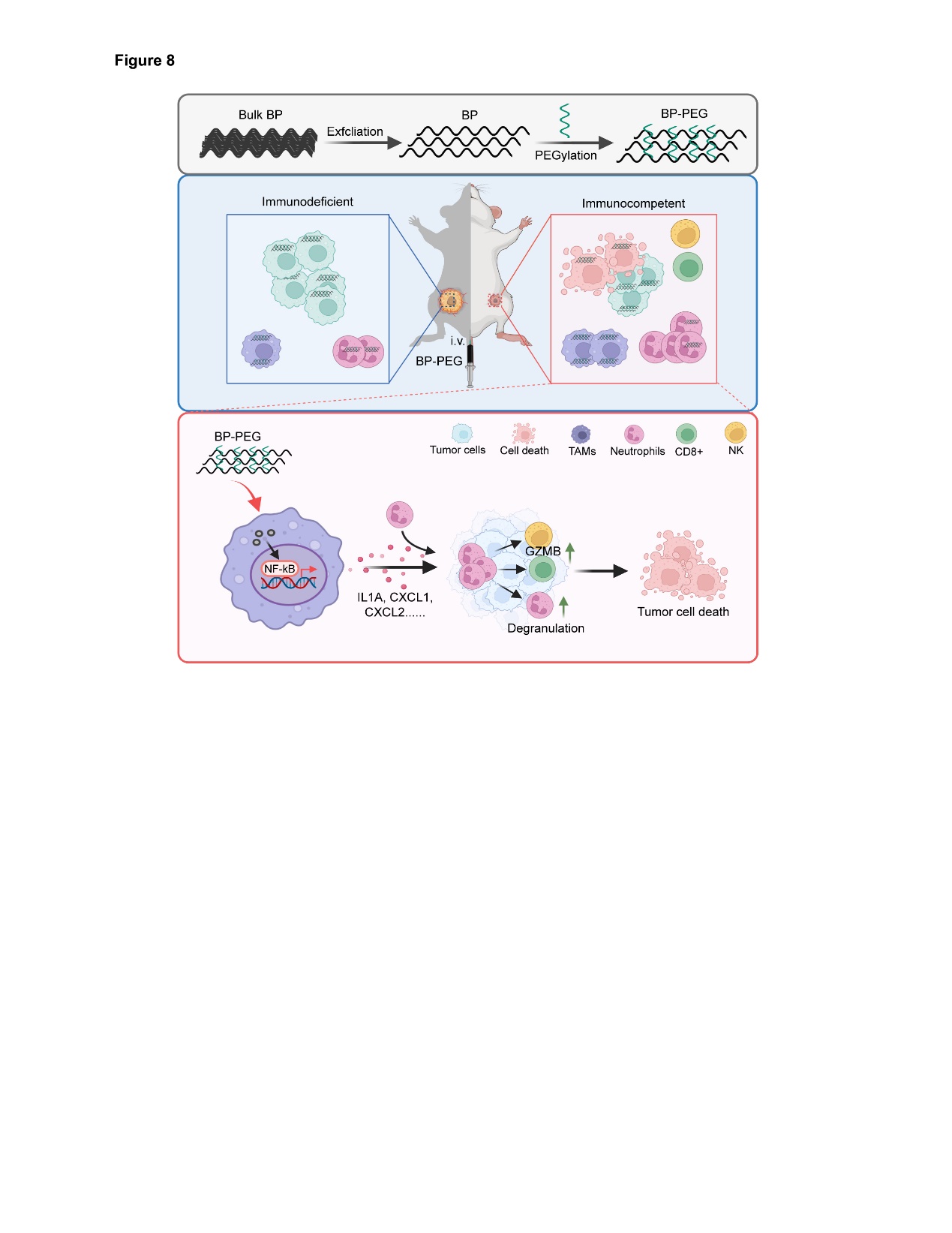


**Figure 8.** Proposed working model (Created in BioRender. Yu, F. (2024), Agreement number: *TE27PIVUES*).

**3. Discussion**

This study demonstrates that black phosphorus-polyethylene glycol (BP-PEG) nanosheets exert significant anti-tumor effects in breast cancer models by modulating the tumor immune microenvironment (TME), specifically by promoting neutrophil recruitment and activation. Unlike conventional chemotherapeutics that directly target tumor cells, BP-PEG enhances innate immune responses, leading to inhibition of tumor growth and suppression of metastasis. Importantly, BP-PEG does not directly induce cytotoxicity in tumor cells at physiologically relevant concentrations; instead, its anti-tumor efficacy is mediated through immune modulation. This is evidenced by the lack of therapeutic effect in immunodeficient NSG mice and the reversal of anti-tumor activity upon neutrophil depletion in immunocompetent mice, underscoring the pivotal role of neutrophils in BP-PEG-mediated tumor suppression. Transcriptomic analyses revealed upregulation of cytokines and chemokines such as IL-1α and CXCL2, which are crucial for neutrophil recruitment and activation. Furthermore, mass cytometry and single-cell RNA sequencing confirmed enhanced neutrophil infiltration and functional activation. Thus, BP-PEG reprograms the TME from immunosuppressive to immunostimulatory, promoting anti-tumor immunity. Overall, our study demonstrates that neutrophils exhibit remarkable plasticity and can acquire an anti-tumor phenotype in response to BP-PEG therapy.

Neutrophils have a dual function in tumor immunity that depends on both context and specific subsets. Historically, they have been considered pro-tumorigenic because they promote angiogenesis, facilitate metastasis, and suppress adaptive immunity[31]. Specifically, type 2 tumor-associated neutrophils have been linked to oncogenesis, tumor progression, metastasis, and the suppression of adaptive immune responses[23]. An increased neutrophil-to-lymphocyte ratio is viewed as a strong predictor of poor prognosis in various cancer types and is associated with reduced effectiveness of immunotherapies[32].

However, neutrophils are a heterogeneous population, and under certain conditions, they can exert anti-tumor effects[33, 34]. Under specific conditions of acute inflammation or in response to targeted therapeutic interventions, neutrophils can adopt anti-tumorigenic phenotypes. For instance, they can enhance antigen presentation and co-stimulation of T cells, thereby initiating adaptive immune responses[35]. Studies have demonstrated that alleviating hypoxia can increase neutrophil infiltration into tumors, leading to tumor cell killing through mechanisms involving NADPH oxidase, reactive oxygen species (ROS), and matrix metalloproteinase-9 (MMP-9)[30]. And neutrophil elastase, which induces apoptosis in tumor cells by cleaving the death domain of CD95[36]. Therefore, the role of neutrophils in cancer is complex and highly dependent on the context.

Our study demonstrated that BP-PEG nanosheets appear to polarize neutrophils toward an anti-tumor phenotype, characterized by increased degranulation, production of ROS, and expression of cytotoxic mediators. Upon UMAP and clustering of scRNA-seq data of tumors from treated mice, we found the neutrophils showed increased in the innate immune response, neutrophil degranulation, myeloid leukocyte migration, and oxidative stress and redox pathways, suggesting that highly differentiated neutrophils adopt an anti-tumor phenotype. And the interaction between macrophages and neutrophils is critical for the immunomodulatory effects of BP-PEG (Fig. 8). Uptake of BP-PEG by macrophages activates inflammatory signaling pathways, leading to the secretion of chemokines that recruit neutrophils to the tumor site (Fig. 8). This creates a positive feedback loop that amplifies the anti-tumor immune response. Enhanced interactions among macrophages, neutrophils, natural killer (NK) cells, and CD8⁺ T cells emphasize the importance of immune cell crosstalk in BP-PEG-mediated tumor suppression (Fig. 8).

Much of our current understanding of neutrophils' impact on tumor growth and therapy response derives from animal studies where neutrophils are broadly depleted during treatment[37]. However, given the emerging evidence of diverse neutrophil subsets with varying functions within tumors, indiscriminate depletion strategies may eliminate both pro-tumorigenic and anti-tumorigenic neutrophil populations[25]. From a therapeutic standpoint, reprogramming tumor-associated neutrophils toward an anti-tumor phenotype may be more effective for tumor control than complete neutrophil depletion[25]. Therefore, gaining a deeper understanding of how BP-PEG influences the acquisition of distinct neutrophil states would allow for selective manipulation of neutrophil subpopulations. This, in turn, would enable us to develop a more nuanced understanding of the role of neutrophils in immunotherapy.

The immunomodulatory properties of BP-PEG nanosheets offer potential for integration into existing cancer immunotherapies. For example, combining BP-PEG with immune checkpoint inhibitors may potentiate T-cell-mediated anti-tumor responses by alleviating immunosuppression within the TME[38, 39]. Furthermore, BP-PEG’s ability to harness innate immune responses, particularly through neutrophil-mediated mechanisms, offers a complementary strategy to current therapies. This approach may address limitations of conventional chemotherapies, which often exacerbate neutrophil infiltration and contribute to chemoresistance [28]. To facilitate clinical translation, comprehensive investigations into the long-term biodistribution, biodegradation, and clearance of BP-PEG are essential to establish its safety profile. Additionally, elucidating the molecular mechanisms underlying its immune modulation will be crucial for therapeutic optimization [40]. Additionally, exploring the effects of BP-PEG on other immune cell subsets, such as regulatory T cells and myeloid-derived suppressor cells, could provide deeper insights into its immunomodulatory capacity[40]. Future research should evaluate BP-PEG in other tumor models and metastatic contexts will help validate its broad applicability. This research enhances our understanding of BP's interactions with the immune system and highlights BP-PEG's potential as a novel immunotherapeutic agent.

Additionally, BP has emerged as promising nanomaterials for enhancing wound healing processes due to their unique structural, chemical, and functional properties [41-43] The two-dimensional (2D) structure of BP-PEG offers a high surface area-to-volume ratio, facilitating extensive interactions with biological molecules and cells at the wound site[44]. This high surface area enhances the adsorption of proteins, cytokines, and growth factors essential for wound healing[41, 44]. Additionally, BP-PEG’s ability to generate reactive oxygen species (ROS) provides antimicrobial protection while promoting angiogenesis and tissue regeneration, thereby accelerating the wound healing process[41, 45]. Our study on BP’s modulation of neutrophils and macrophages provides key insights into its potential for enhancing immune responses and promoting tissue regeneration. We believe these findings could be leveraged for a range of biomedical applications, especially for treating infected wounds, where BP’s dual actions can significantly improve healing outcomes

There are still some limitations of our studies, Firstly, the concentration range of BP-PEG used in vivo requires further detailed investigation. It is crucial to identify an optimal concentration window where BP-PEG can effectively elicit an anti-tumor immune response without causing toxicity to normal tissues. Determining this balance will help ensure the therapeutic efficacy of BP-PEG while minimizing potential adverse effects on healthy cells. Secondly, the precise mechanisms by which BP-PEG is internalized by macrophages and neutrophils, induces the expression of inflammatory factors within the tumor microenvironment, and reprograms neutrophils toward anti-tumor functions need to be elucidated. Specifically, understanding how BP-PEG influences neutrophil reprogramming will provide deeper insights into its immunomodulatory effects and enhance the development of targeted cancer therapies. Addressing these limitations in future studies will strengthen the understanding and application of BP-PEG in cancer immunotherapy.

**4. Conclusion**

In conclusion, BP-PEG nanosheets effectively modulate the tumor immune microenvironment to inhibit breast cancer growth and metastasis by promoting neutrophil-dependent anti-tumor immunity. These findings advance our understanding of BP-PEG's mechanisms of action and highlight the potential of nanomaterial-based strategies in reprogramming tumor-associated neutrophils toward an anti-tumor phenotype to favor anti-tumor responses.

**5. Experimental Section**

*Synthesis of BP-PEG:* BP crystals were exfoliated in N-methyl-2-pyrrolidone (NMP) through ultrasonic treatment for 6 hours. Following this, 1 mg of BPNS precipitate and 10 mg of PEG-NH2 were dispersed in deionized water. The resulting mixture underwent sonication for 15 minutes and was then stirred for 4 hours. To remove any unreacted PEG-NH2, the solution was centrifuged at 12,000 rpm for 10 minutes. The obtained BP-PEG dispersion was subsequently washed with deionized water to eliminate residual PEG and solvents, and it was stored in the dark at 4°C. The characteristics of the BP-PEG nanosheets were then analyzed using several techniques, including Transmission Electron Microscopy (TEM), Zeta Potential Measurement, Energy-Dispersive X-ray Spectroscopy (EDS), Fourier-Transform Infrared Spectroscopy (FTIR), X-ray Diffraction (XRD), Raman Spectroscopy, and UV-Visible Spectroscopy.

*Cell line and cell culture:* 4T1 cells were cultured in RPMI 1640 medium (Gibco) supplemented with 10% fetal bovine serum (FBS) and 1% penicillin–streptomycin. And MDA-MB-231 cells were maintained in DMEM (Gibco) with the same supplements of 10% FBS and 1% penicillin–streptomycin. All cell lines were incubated in a humidified environment at 37 °C with 5% CO₂.

*Mouse models:* Six- to eight-week-old female BALB/c and NSG mice were purchased from Shanghai SLAC Laboratory Animal and maintained under specific pathogen-free conditions with ad libitum access to food and water. All animal experiments were conducted in accordance with the experimental animal guidelines of the University of Science and Technology of China (USTCACUC27120124035). For the orthotopic transplantation experiment, 5 × 10^5^ 4T1-luciferase cells were resuspended in 15μL PBS and mixed with an equal volume (15μL) of Matrigel (Corning, 354234). The mixture was then injected into the fourth and ninth mammary glands of the mice. When the tumors reached a volume of 100-150 mm³, mice were randomly divided into two or four groups. BP-PEG (10 mg/kg) was administered intravenously every other day. Anti-Ly6G antibody (Bio X Cell, # BE0075-1) was administered intraperitoneally at 12.5μg in 100μL PBS per mouse daily, starting three days post-tumor inoculation. Ultra-LEAF™ Purified anti-Asialo-GM1 Antibody (Biolegend, #146002) was administered intraperitoneally at 25μL per mouse every three days. SB225002 (MedChemExpress, #HY-16711) (10 mg/kg) was administered intraperitoneally every 3 days. Tumor growth was monitored using calipers every three days, and tumor volume was calculated using the formula: 0.5 × length × width × width.

FVB-MMTV-PyMT(no.T004993) transgenic mice were purchased from GemPharmatech. When the tumor volume reached 100-150 mm³, mice were randomly assigned to two groups and received either BP-PEG (10 mg/kg) or vehicle via tail vein injection. Tumor growth was monitored as described above. All mice were euthanized using CO₂ inhalation.

For in vivo imaging, mice were intravenously injected with fluorescein isothiocyanate (FITC)-labeled BP-PEG at a dose of 10 mg/kg 24 hours before sacrifice. Organs were collected, and imaging was performed using an Animal Imaging System (IVIS Spectrum).

*Bulk RNA-seq:* Fresh mammary tumors were collected two days after treatment with BP-PEG or vehicle. RNA was extracted from the tumors using TRIzol Reagent (Invitrogen, Carlsbad, CA, USA) following the manufacturer's protocol. Library preparation was conducted using the VAHTS Universal V6 RNA-seq Library Prep Kit for Illumina®, according to Illumina's standard instructions. An Agilent 4200 Bioanalyzer was employed to assess the concentration and size distribution of the cDNA library before sequencing on an Illumina NovaSeq 6000. High-throughput sequencing was performed according to the manufacturer's instructions (Illumina). Raw reads were filtered using seqtk before being mapped to the genome with HISAT2 (version 2.0.4). Gene fragments were counted using StringTie (version 1.3.3b), followed by TMM (trimmed mean of M-values) normalization. Differential expression analysis was conducted using the edgeR package. Significantly differentially expressed genes (DEGs) were defined as those with a false discovery rate (FDR) q-value less than 0.05 and a fold-change greater than 2. KEGG (Kyoto Encyclopedia of Genes and Genomes) pathway enrichment analysis was performed using the appropriate R package.

*Quantitative Reverse Transcription PCR (qRT-PCR):* qRT-PCR was performed as previously described. Briefly, total RNA was extracted from cells using Trizol Reagent (Invitrogen, Carlsbad, CA, USA) according to the manufacturer's protocol. The RNA concentration was determined using a NanoDrop spectrophotometer (Life Technologies, Carlsbad, CA, USA). cDNA was synthesized from 1 µg of RNA using Oligo(dT) primers and Superscript III reverse transcriptase, following the manufacturer’s protocol (Vazyme, China). Quantitative PCR was carried out using SYBR® Premix (Vazyme, China). Data were collected using a PIKOREAL 96 real-time PCR system (Thermo Scientific, MA, USA). All reactions were performed in triplicate. The primers used in this study are listed in the key resources table (Table S1). Primers were designed using PrimerBank or OriGene (OriGene Technologies, Inc.) and verified for specificity using the NCBI Primer-BLAST tool. The primers were synthesized by SANGON (Shanghai, China).

*Flow cytometry analysis (FACS):*

Tumors were collected immediately after euthanizing the mice. Tumor tissues were dissociated into single-cell suspensions using collagenase treatment. The resulting single-cell suspension was incubated with Red Blood Cell Lysis Buffer (Beyotime Biotechnology) for 10-15 minutes at 4°C to lyse red blood cells. Cells were then washed, counted using an automated cell counter (Countstar), and 1 × 10⁶ cells were blocked with FcR Blocking Reagent (BioLegend) or mouse serum. The cells were subsequently stained for cell surface markers using antibodies purchased from BioLegend. Flow cytometry analysis was performed using a CytoFLEX flow cytometer (BD Biosciences), and the data were analyzed with CytExpert software (version 2.4.0.28) and FlowJo software (Tree Star, Inc., Ashland, OR).

*Mass Cytometry (CyTOF):* Tumor tissues harvested from the mice were processed into single-cell suspensions using the same methods as those used for flow cytometry. A total of 40 metal-conjugated antibodies were pre-prepared using the Maxpar Antibody Labeling Kit, following the manufacturer’s instructions (Supplementary Table S1). The qualified cell samples were then incubated with these antibodies for surface marker staining. After washing, the cells were analyzed using the Helios3 CyTOF system (Fluidigm, USA) for signaling detection. Data acquisition and analysis were performed by PLTTech Inc. (Hangzhou, China). Briefly, after normalization with the EQ Four Element Calibration Beads, CyTOF data were analyzed on the Cytobank platform (<https://www.cytobank.org/>). Nonlinear t-distributed stochastic neighbor embedding (t-SNE) dimensionality reduction and unsupervised PhenoGraph clustering were conducted using the cytofkit package in R to identify distinct immune cell populations.

*Single-Cell RNA Sequencing:* Tumor tissues harvested from mice were processed into single-cell suspensions using the same methods employed for flow cytometry. The single-cell suspensions were stained with a fluorochrome-labeled CD45 antibody for sorting and labeled with DAPI to identify live cells. CD45⁺ cells were isolated using a Beckman cell sorter.

Cell suspensions were prepared using the Shbio® Tissue Dissociation Kit (21517-10) for tissue samples. The prepared cell suspensions, barcoded gel beads, and oil were loaded into separate wells of the Chromium Chip K. Using the 10x Genomics Chromium system, Gel Beads-in-Emulsion (GEMs) were generated. The GEMs were then transferred to a PCR instrument for reverse transcription. The barcoded gel beads contained 30-nucleotide oligo-dT reverse transcription primers, enabling poly-A RNA from the cells to be reverse-transcribed into first-strand cDNA tagged with barcodes and Unique Molecular Identifiers (UMIs). Following reverse transcription, magnetic beads were used to purify the cDNA. The purified cDNA was then amplified by PCR. The concentration of cDNA was measured using a Qubit fluorometer, and fragment size was assessed using an Agilent 2100 Bioanalyzer.

After cDNA amplification, the products were enzymatically fragmented and size-selected using magnetic beads to obtain optimal fragment sizes. End repair, A-tailing, and adapter ligation were performed to introduce the Read 2 sequencing primer. PCR was then used to construct cDNA libraries containing P5 and P7 adapters. The libraries were purified using magnetic beads. Library concentration was measured using a Qubit fluorometer, and fragment size was assessed using an Agilent 4200 Bioanalyzer. Cluster generation and hybridization of the first read sequencing primer were performed according to the Illumina User Guide. The flow cell containing the clusters was loaded onto the sequencing machine. Paired-end sequencing was performed, controlled by the data collection software provided by Illumina, with real-time data analysis during the sequencing process.

*Single-Cell RNA Sequencing data analyze:* Data Processing and Initial Analysis: Raw single-cell RNA sequencing (scRNA-seq) data were processed using CellRanger software (10X Genomics)[46] to perform alignment, gene quantification, and cell identification, converting FASTQ files into cell expression matrices. Based on the cell types and quantification results identified by CellRanger, the Seurat package[47] was used for secondary filtering and analysis of the data, including subcluster analysis, Gene Ontology (GO) and Kyoto Encyclopedia of Genes and Genomes (KEGG) enrichment analysis, and cell-cell communication analysis.

Quality Control and Filtering: For each sample, we applied a tailored scRNA-seq data analysis pipeline to cluster detected cells and identify cell types, enabling comparisons of cell composition and abundance across different samples. Data preprocessing and normalization can affect subsequent cell classification results; therefore, different datasets require specific filtering methods to avoid inaccuracies due to excessive abnormal cells or genes. Considering batch differences caused by experimental or sequencing factors (such as sequencing depth, UMI counts, gene expression levels, mitochondrial, and ribosomal content), applying the same filtering thresholds across all samples is unreasonable. We statistically analyzed the distributions of UMI counts, gene counts, mitochondrial gene percentages, and ribosomal gene percentages for each sample to calculate appropriate filtering thresholds. Cells with high mitochondrial gene content indicate potential cell death; thus, we set an upper mitochondrial gene percentage threshold[48]. Cells with erythrocyte gene percentages exceeding 1% were filtered out. If the mitochondrial gene percentage filtering threshold for an individual sample exceeded 25%, we re-filtered using a threshold of 25%. As scRNA-seq typically encapsulates cells into droplets, with each droplet ideally containing a single cell associated with a barcode, increasing cell loading can lead to a higher probability of droplets containing two or more cells (doublets or multiplets). These doublets share the same barcode and are treated as single cells, potentially introducing erroneous information into subsequent analyses. Quality control based on total reads per cell, genes per cell, and mitochondrial gene percentage can filter out most doublets; however, some may remain. Therefore, we used the Scrublet algorithm[49] to further filter doublets.

Doublet Detection Using Scrublet: The Scrublet algorithm simulates doublets by randomly pairing barcodes and adding them to the original expression matrix. All cells (including simulated doublets) are then clustered, and each cell is assigned a doublet score based on clustering results. The doublet score is proportional to the number of associated simulated doublets; higher scores indicate a higher likelihood of being a true doublet[49].

Cell Cycle Analysis: Cell cycle scores were calculated for each cell based on the expression of cell cycle genes[50], determining the cell cycle stage of each cell. Principal component analysis (PCA) was used to visualize and evaluate cell cycle effects. If the effect was significant, linear regression was employed to remove cell cycle effects. In this experiment, cell cycle effects were minimal and no adjustment was made.

Highly Variable Gene Identification and Clustering: We identified the top 2,000 variable genes for each sample based on the mean expression and dispersion (variance/mean) for subsequent integration analysis [51]. Specifically, genes were grouped into 20 bins based on average expression. The normalized dispersion for each gene was calculated as the absolute difference between the variance and the median variance of genes within the same bin. Integrated data were subjected to PCA for dimensionality reduction. The Louvain algorithm, also known as graph-based clustering, was applied to the normalized data for clustering analysis. This algorithm is widely used in scRNA-seq data analysis published in high-impact journals and is effective in identifying hierarchical community structures by optimizing the modularity of the community network.

Dimensionality Reduction and Visualization: Uniform Manifold Approximation and Projection (UMAP), a manifold learning technique for dimensionality reduction, was used for data visualization. UMAP offers visualization quality comparable to t-SNE, retains more global structure, and has excellent runtime performance, making it suitable as a general-purpose dimensionality reduction technique for machine learning.

Differential Expression and Functional Enrichment Analysis: Clustering analysis divided the cells into distinct clusters. Based on sample grouping information, we performed inter-group differential analysis for each cluster to identify differentially expressed genes (DEGs). DEGs were screened based on fold change (FC > 1.5 or FC < 0.66) and p-value < 0.05. GO and KEGG enrichment analyses were conducted on the DEGs to identify significantly enriched functional pathways, aiming to explore functional heterogeneity among sample groups.

Marker Gene Identification and Cluster Characterization: We analyzed marker genes for all clusters using the Wilcoxon algorithm, employing a group one vs. rest approach to score marker genes. Genes that were specifically highly expressed (logFC > 0.25) in each cluster and expressed in at least 20% of cells were selected as significant marker genes. While marker genes represent the characteristic genes expressed by each cluster, individual genes may not fully reflect the cluster's properties. Therefore, we performed GO and KEGG enrichment analyses on the marker genes of each cluster to further infer the cellular functional characteristics through the pathways and GO terms associated with the marker genes.

Cell Type Annotation: Cell types were annotated using SingleR[52] and marker genes. SingleR selects RNA-seq data as a reference, identifies genes with high variability between different cell types in the reference database, and calculates the correlation between the predicted cells and the reference database. By iteratively removing the cell type with the lowest correlation, SingleR ultimately annotates the predicted cell types.

Additionally, based on literature, we collected marker gene lists related to all expected cell types. SCINA (Semi-supervised Category Identification and Assignment)[53] assigned cell type labels to individual cells based on the expression levels of marker genes. SCINA is an automated algorithm for cell type detection and assignment in scRNA-seq data, capable of assigning cell identities to all cells based on prior knowledge of features (marker genes) highly (or lowly) expressed in each cell group.

Based on the marker genes provided in SCINA and the semi-supervised annotation results, various visualization methods (heatmaps, bubble plots, UMAP/t-SNE plots, violin plots) were used to display the expression characteristics of marker genes in various cell groups.

Cell-Cell Communication Analysis: Cell communication occurs throughout organisms, forming complex regulatory networks. Signal interactions mainly include adjacency-type and diffusion-type transmission methods. Adjacency-type transmission includes nerve signal transmission and antigen presentation, characterized by signal transmission between two closely associated cells, such as direct binding of membrane-bound ligand-receptor pairs. Diffusion-type transmission primarily involves secreted proteins diffusing through the extracellular matrix or body fluids to other cells, with endocrine, paracrine, and autocrine being typical examples.

We used the CellChat[54] analysis method to infer, visualize, and analyze cell-cell communication networks. CellChat models communication probabilities and identifies important communication networks by subdividing detected ligand-receptor pairs into signaling pathways. CellChat constructed a cell communication reference database-CellChatDB-by selecting 2,021 validated cell communication relationships (applicable to humans and mice), considering multi-subunit receptors and other important signaling auxiliary factors, including soluble agonists, antagonists, co-stimulatory, and co-inhibitory membrane-bound receptors.

*In Vitro Cytotoxicity Assay:* Approximately 3,000 cells per well were seeded in 96-well plates 24 hours before the addition of BP-PEG. The cells were then treated with varying concentrations of BP-PEG (0–50 µg/mL) for 24–72 hours. Cell viability was assessed using the Cell Counting Kit-8 (CCK-8) assay, following the manufacturer’s instructions. All samples were analyzed in triplicate, and data are presented as the mean ± standard deviation from 2–3 independent experiments.

*Co-Culture Assay:* Peripheral blood samples were collected from breast cancer patients prior to surgery, in accordance with the experimental guidelines of the University of Science and Technology of China (2022KY256). To establish an in vitro co-culture system, PBMCs were isolated from the blood samples using Ficoll-Paque density gradient centrifugation. The isolated PBMCs were then co-cultured with MDA-MB-231 cells at a 10:1 ratio for 48 hours, with or without BP-PEG. Viable MDA-MB-231 cells were captured and counted before processing for crystal violet staining. The PBMC-containing suspension was also sent for flow cytometry analysis.

For the co-culture of neutrophils with 4T1 cells, single cells from tumors were isolated 48 hours after BP-PEG treatment, as described for flow cytometry. Neutrophils were isolated from the resulting cell suspension using magnetic selection with anti-Ly-6G microbeads (Miltenyi Biotec). Isolated neutrophils were co-cultured with 4T1-H2B-GFP tumor cells at a 40:1 neutrophil-to-cancer cell ratio. GFP+ tumor cells were quantified 24 hours later by microscopy.

*Histology and Immunohistochemistry (IHC):* Tissue specimens from mice were fixed in 10% buffered formalin for 24 hours and then stored in 70% ethanol until paraffin embedding. Sections of 5 µm were cut and stained with hematoxylin and eosin (H&E) or used for immunohistochemical analysis. Immunohistochemistry was performed on formalin-fixed, paraffin-embedded tumor tissue sections using the biotin-avidin method, as previously described. Sections were stained with antibodies against Ly6G (neutrophil marker) and granzyme B (GZMB). The immunoreactivity was developed using DAB, followed by counterstaining with hematoxylin. Images were captured using the 3DHISTECH Pannoramic MiDi slide scanner equipped with a 20× objective lens.

*Enzyme-linked immunosorbent assay (ELISA):*Cells were treated according to their respective groups. Subsequently, the culture medium from each group was collected. After centrifugation, the supernatant was analyzed for CXCL2 concentrations using an ELISA assay (Lianke Biotechnology, Hangzhou, China). Following the addition of the stop solution, absorbance was measured at 450 nm and 630 nm using a microplate reader.

*Drawing Schematic diagram:* All Schematic diagrams in this work were the Created in BioRender. Yu, F. (2024), Agreement number: *KP27PKUH9C*). Including images in Figure 1D, 1F, S1H, 2A, 2F, 3A, 3E, 5A, 6A, 6H, 7E , S7D and Figure 8.

*Statistical Analysis:* The quantification of data analyses was mainly performed with GraphPad Prism statistical software, except bulk RNAseq and scRNAseq. The detail of analyze processing of bulk RNAseq and scRNAseq were describe in related sections. For vivo assay, at least 5 mice were used for each group, and at least three samples or independent experiments were performed for other experiments. P value was determined by unpaired t-tests. A value of p < 0.05 was considered statistically significant.

**Supporting Information**

Supporting Information is available on the Wiley Online Library or can be obtained from the corresponding authors upon request.

**Conflict of Interest**

The authors declare that they have no conflicts of interest.

**Author Contributions**

J.W. conducted the experiments and supported data acquisition. W.Q.Y. performed the analysis of all sequencing data. Y.X.S., H.J.Z., and B.Y.Z. prepared and characterized the various forms of Black Phosphorus used in this study. H.S. assisted to data analysis and interpretation. X.P. assisted to ELISA assay. Z.Y.Y., M.X.P., and Y.H. supervised manuscript revisions. F.Z.Y. conceptualized the project, conducted experiments, analyzed and interpreted the data, and authored the manuscript. Z.Y.Y., F.Z.Y. provided project oversight and supervised manuscript revisions. All authors reviewed and approved the final version of the manuscript for submission.

**Data Availability Statement**

The data supporting the findings of this study are available from the main corresponding author Fazhi Yu upon reasonable request.

Acknowledgements

This work is supported by: The Strategic Priority Research Program of the Chinese Academy of Sciences, Grant XDB0940101, National Science Foundation of China 32370776,92357301, 32170736, National Key R&D Program of China 2022YFA1303100, Noncommunicable Chronic Diseases-National Science and Technology Major Project 2023ZD0507500, Research Funds of Center for Advanced Interdisciplinary Science and Biomedicine of IHM of USTC QYPY20220017. And Fundamental Research Funds for the Central Universities YD9110002042. Anhui Province in 2022 Innovation and Entrepreneurship Support Plan Project (2022LCX031)

**References**

[1] K. Hu, L. Xie, Y. Zhang, M. Hanyu, Z. Yang, K. Nagatsu, H. Suzuki, J. Ouyang, X. Ji, J. Wei, H. Xu, O.C. Farokhzad, S.H. Liang, L. Wang, W. Tao, M.R. Zhang, Marriage of black phosphorus and Cu(2+) as effective photothermal agents for PET-guided combination cancer therapy, Nat Commun, 11 (2020) 2778.

[2] H. Wang, X. Yang, W. Shao, S. Chen, J. Xie, X. Zhang, J. Wang, Y. Xie, Ultrathin Black Phosphorus Nanosheets for Efficient Singlet Oxygen Generation, J Am Chem Soc, 137 (2015) 11376-11382.

[3] F. Qi, P. Ji, Z. Chen, L. Wang, H. Yao, M. Huo, J. Shi, Photosynthetic Cyanobacteria-Hybridized Black Phosphorus Nanosheets for Enhanced Tumor Photodynamic Therapy, Small, 17 (2021) e2102113.

[4] L. Cheng, X. Wang, F. Gong, T. Liu, Z. Liu, 2D Nanomaterials for Cancer Theranostic Applications, Adv Mater, 32 (2020) e1902333.

[5] M. Buscema, D.J. Groenendijk, G.A. Steele, H.S. van der Zant, A. Castellanos-Gomez, Photovoltaic effect in few-layer black phosphorus PN junctions defined by local electrostatic gating, Nat Commun, 5 (2014) 4651.

[6] F. Xia, H. Wang, Y. Jia, Rediscovering black phosphorus as an anisotropic layered material for optoelectronics and electronics, Nat Commun, 5 (2014) 4458.

[7] S. Kim, G. Myeong, W. Shin, H. Lim, B. Kim, T. Jin, S. Chang, K. Watanabe, T. Taniguchi, S. Cho, Thickness-controlled black phosphorus tunnel field-effect transistor for low-power switches, Nat Nanotechnol, 15 (2020) 203-206.

[8] R. Gui, H. Jin, Z. Wang, J. Li, Black phosphorus quantum dots: synthesis, properties, functionalized modification and applications, Chem Soc Rev, 47 (2018) 6795-6823.

[9] W. Chen, J. Ouyang, H. Liu, M. Chen, K. Zeng, J. Sheng, Z. Liu, Y. Han, L. Wang, J. Li, L. Deng, Y.N. Liu, S. Guo, Black Phosphorus Nanosheet-Based Drug Delivery System for Synergistic Photodynamic/Photothermal/Chemotherapy of Cancer, Adv Mater, 29 (2017).

[10] L. Qin, S. Jiang, H. He, G. Ling, P. Zhang, Functional black phosphorus nanosheets for cancer therapy, J Control Release, 318 (2020) 50-66.

[11] K.E. de Visser, J.A. Joyce, The evolving tumor microenvironment: From cancer initiation to metastatic outgrowth, Cancer Cell, 41 (2023) 374-403.

[12] C. Swanton, E. Bernard, C. Abbosh, F. Andre, J. Auwerx, A. Balmain, D. Bar-Sagi, R. Bernards, S. Bullman, J. DeGregori, C. Elliott, A. Erez, G. Evan, M.A. Febbraio, A. Hidalgo, M. Jamal-Hanjani, J.A. Joyce, M. Kaiser, K. Lamia, J.W. Locasale, S. Loi, I. Malanchi, M. Merad, K. Musgrave, K.J. Patel, S. Quezada, J.A. Wargo, A. Weeraratna, E. White, F. Winkler, J.N. Wood, K.H. Vousden, D. Hanahan, Embracing cancer complexity: Hallmarks of systemic disease, Cell, 187 (2024) 1589-1616.

[13] I. Mellman, D.S. Chen, T. Powles, S.J. Turley, The cancer-immunity cycle: Indication, genotype, and immunotype, Immunity, 56 (2023) 2188-2205.

[14] S.I. Grivennikov, F.R. Greten, M. Karin, Immunity, inflammation, and cancer, Cell, 140 (2010) 883-899.

[15] D. Bruni, H.K. Angell, J. Galon, The immune contexture and Immunoscore in cancer prognosis and therapeutic efficacy, Nat Rev Cancer, 20 (2020) 662-680.

[16] D.S. Chen, I. Mellman, Elements of cancer immunity and the cancer-immune set point, Nature, 541 (2017) 321-330.

[17] D.J. Irvine, E.L. Dane, Enhancing cancer immunotherapy with nanomedicine, Nat Rev Immunol, 20 (2020) 321-334.

[18] R. Kuai, L.J. Ochyl, K.S. Bahjat, A. Schwendeman, J.J. Moon, Designer vaccine nanodiscs for personalized cancer immunotherapy, Nat Mater, 16 (2017) 489-496.

[19] M. Wu, X. Niu, R. Zhang, Z. Ping Xu, Two-dimensional nanomaterials for tumor microenvironment modulation and anticancer therapy, Adv Drug Deliv Rev, 187 (2022) 114360.

[20] L. Zhang, C. Zhu, J. Zhao, L. Scimeca, M. Dong, R. Liu, Y. Jia, Z.P. Xu, Recent Advances in Nanomodulators for Augmenting Cancer Immunotherapy in Cold Tumors: Insights from Drug Delivery to Drug‐Free Strategies, Advanced Functional Materials, 34 (2024).

[21] Z. Li, Y. Hu, Q. Fu, Y. Liu, J. Wang, J. Song, H. Yang, NIR/ROS‐Responsive Black Phosphorus QD Vesicles as Immunoadjuvant Carrier for Specific Cancer Photodynamic Immunotherapy, Advanced Functional Materials, 30 (2019).

[22] G.L. Burn, A. Foti, G. Marsman, D.F. Patel, A. Zychlinsky, The Neutrophil, Immunity, 54 (2021) 1377-1391.

[23] M.E. Shaul, Z.G. Fridlender, Tumour-associated neutrophils in patients with cancer, Nat Rev Clin Oncol, 16 (2019) 601-620.

[24] S. Jaillon, A. Ponzetta, D. Di Mitri, A. Santoni, R. Bonecchi, A. Mantovani, Neutrophil diversity and plasticity in tumour progression and therapy, Nat Rev Cancer, 20 (2020) 485-503.

[25] T. Nemeth, M. Sperandio, A. Mocsai, Neutrophils as emerging therapeutic targets, Nat Rev Drug Discov, 19 (2020) 253-275.

[26] X. Liang, X. Ye, C. Wang, C. Xing, Q. Miao, Z. Xie, X. Chen, X. Zhang, H. Zhang, L. Mei, Photothermal cancer immunotherapy by erythrocyte membrane-coated black phosphorus formulation, Journal of Controlled Release, 296 (2019) 150-161.

[27] Y. Zhang, C. Ma, J. Xie, H. Agren, H. Zhang, Black Phosphorus/Polymers: Status and Challenges, Adv Mater, 33 (2021) e2100113.

[28] A. Mousset, E. Lecorgne, I. Bourget, P. Lopez, K. Jenovai, J. Cherfils-Vicini, C. Dominici, G. Rios, C. Girard-Riboulleau, B. Liu, D.L. Spector, S. Ehmsen, S. Renault, C. Hego, F. Mechta-Grigoriou, F.C. Bidard, M.G. Terp, M. Egeblad, C. Gaggioli, J. Albrengues, Neutrophil extracellular traps formed during chemotherapy confer treatment resistance via TGF-beta activation, Cancer Cell, 41 (2023) 757-775 e710.

[29] Z. Granot, E. Henke, E.A. Comen, T.A. King, L. Norton, R. Benezra, Tumor entrained neutrophils inhibit seeding in the premetastatic lung, Cancer Cell, 20 (2011) 300-314.

[30] M.S.F. Ng, I. Kwok, L. Tan, C. Shi, D. Cerezo-Wallis, Y. Tan, K. Leong, G.F. Calvo, K. Yang, Y. Zhang, J. Jin, K.H. Liong, D. Wu, R. He, D. Liu, Y.C. Teh, C. Bleriot, N. Caronni, Z. Liu, K. Duan, V. Narang, I. Ballesteros, F. Moalli, M. Li, J. Chen, Y. Liu, L. Liu, J. Qi, Y. Liu, L. Jiang, B. Shen, H. Cheng, T. Cheng, V. Angeli, A. Sharma, Y.H. Loh, H.L. Tey, S.Z. Chong, M. Iannacone, R. Ostuni, A. Hidalgo, F. Ginhoux, L.G. Ng, Deterministic reprogramming of neutrophils within tumors, Science, 383 (2024) eadf6493.

[31] S.B. Coffelt, M.D. Wellenstein, K.E. de Visser, Neutrophils in cancer: neutral no more, Nat Rev Cancer, 16 (2016) 431-446.

[32] T. Barbui, A. Carobbio, A. Ghirardi, F. Fenili, M.C. Finazzi, M. Castelli, A.M. Vannucchi, P. Guglielmelli, A. Rambaldi, N. Gangat, A. Tefferi, Neutrophil-to-lymphocyte ratio as a prognostic indicator of mortality in Polycythemia Vera: insights from a prospective cohort analysis, Blood Cancer J, 14 (2024) 195.

[33] C. Silvestre-Roig, Z.G. Fridlender, M. Glogauer, P. Scapini, Neutrophil Diversity in Health and Disease, Trends Immunol, 40 (2019) 565-583.

[34] J. Gungabeesoon, N.A. Gort-Freitas, M. Kiss, E. Bolli, M. Messemaker, M. Siwicki, M. Hicham, R. Bill, P. Koch, C. Cianciaruso, F. Duval, C. Pfirschke, M. Mazzola, S. Peters, K. Homicsko, C. Garris, R. Weissleder, A.M. Klein, M.J. Pittet, A neutrophil response linked to tumor control in immunotherapy, Cell, 186 (2023) 1448-1464 e1420.

[35] S. Saha, S.K. Biswas, Tumor-Associated Neutrophils Show Phenotypic and Functional Divergence in Human Lung Cancer, Cancer Cell, 30 (2016) 11-13.

[36] C. Cui, K. Chakraborty, X.A. Tang, G. Zhou, K.Q. Schoenfelt, K.M. Becker, A. Hoffman, Y.F. Chang, A. Blank, C.A. Reardon, H.A. Kenny, T. Vaisar, E. Lengyel, G. Greene, L. Becker, Neutrophil elastase selectively kills cancer cells and attenuates tumorigenesis, Cell, 184 (2021) 3163-3177 e3121.

[37] R. Xue, Q. Zhang, Q. Cao, R. Kong, X. Xiang, H. Liu, M. Feng, F. Wang, J. Cheng, Z. Li, Q. Zhan, M. Deng, J. Zhu, Z. Zhang, N. Zhang, Liver tumour immune microenvironment subtypes and neutrophil heterogeneity, Nature, 612 (2022) 141-147.

[38] W. Tao, X. Zhu, X. Yu, X. Zeng, Q. Xiao, X. Zhang, X. Ji, X. Wang, J. Shi, H. Zhang, L. Mei, Black Phosphorus Nanosheets as a Robust Delivery Platform for Cancer Theranostics, Adv Mater, 29 (2017).

[39] Q. Chen, L. Xu, C. Liang, C. Wang, R. Peng, Z. Liu, Photothermal therapy with immune-adjuvant nanoparticles together with checkpoint blockade for effective cancer immunotherapy, Nat Commun, 7 (2016) 13193.

[40] G. Peng, B. Fadeel, Understanding the bidirectional interactions between two-dimensional materials, microorganisms, and the immune system, Adv Drug Deliv Rev, 188 (2022) 114422.

[41] C. Geng, S. He, S. Yu, H.M. Johnson, H. Shi, Y. Chen, Y.K. Chan, W. He, M. Qin, X. Li, Y. Deng, Achieving Clearance of Drug-Resistant Bacterial Infection and Rapid Cutaneous Wound Regeneration Using an ROS-Balancing-Engineered Heterojunction, Adv Mater, 36 (2024) e2310599.

[42] X. Bai, R. Wang, X. Hu, Q. Dai, J. Guo, T. Cao, W. Du, Y. Cheng, S. Xia, D. Wang, L. Yang, L. Teng, D. Chen, Y. Liu, Two-Dimensional Biodegradable Black Phosphorus Nanosheets Promote Large Full-Thickness Wound Healing through In Situ Regeneration Therapy, ACS Nano, 18 (2024) 3553-3574.

[43] L.S. Smith, H. Haidari, A. Amsalu, G.S. Howarth, S.J. Bryant, S. Walia, A. Elbourne, Z. Kopecki, Black Phosphorus Nanoflakes: An Emerging Nanomaterial for Clinical Wound Management and Biomedical Applications, Int J Mol Sci, 25 (2024).

[44] M. Cui, J. Zhang, P. Han, L. Shi, X. Li, Z. Zhang, H. Bao, Y. Ma, Z. Tao, X. Dong, L. Fu, Y. Wu, Two-dimensional nanomaterials: A multifunctional approach for robust for diabetic wound repair, Mater Today Bio, 28 (2024) 101186.

[45] W. Qi, R. Zhang, Z. Wang, H. Du, Y. Zhao, B. Shi, Y. Wang, X. Wang, P. Wang, Advances in the Application of Black Phosphorus-Based Composite Biomedical Materials in the Field of Tissue Engineering, Pharmaceuticals (Basel), 17 (2024).

[46] G.X.Y. Zheng, J.M. Terry, P. Belgrader, P. Ryvkin, Z.W. Bent, R. Wilson, S.B. Ziraldo, T.D. Wheeler, G.P. McDermott, J. Zhu, M.T. Gregory, J. Shuga, L. Montesclaros, J.G. Underwood, D.A. Masquelier, S.Y. Nishimura, M. Schnall-Levin, P.W. Wyatt, C.M. Hindson, R. Bharadwaj, A. Wong, K.D. Ness, L.W. Beppu, H.J. Deeg, C. McFarland, K.R. Loeb, W.J. Valente, N.G. Ericson, E.A. Stevens, J.P. Radich, T.S. Mikkelsen, B.J. Hindson, J.H. Bielas, Massively parallel digital transcriptional profiling of single cells, Nature Communications, 8 (2017) 14049.

[47] T. Stuart, A. Butler, P. Hoffman, C. Hafemeister, E. Papalexi, W.M. Mauck, Y. Hao, M. Stoeckius, P. Smibert, R. Satija, Comprehensive Integration of Single-Cell Data, Cell, 177 (2019).

[48] T. Ilicic, J.K. Kim, A.A. Kolodziejczyk, F.O. Bagger, D.J. McCarthy, J.C. Marioni, S.A. Teichmann, Classification of low quality cells from single-cell RNA-seq data, Genome Biol, 17 (2016) 29.

[49] S.L. Wolock, R. Lopez, A.M. Klein, Scrublet: Computational Identification of Cell Doublets in Single-Cell Transcriptomic Data, Cell Syst, 8 (2019).

[50] I. Tirosh, B. Izar, S.M. Prakadan, M.H. Wadsworth, D. Treacy, J.J. Trombetta, A. Rotem, C. Rodman, C. Lian, G. Murphy, M. Fallahi-Sichani, K. Dutton-Regester, J.-R. Lin, O. Cohen, P. Shah, D. Lu, A.S. Genshaft, T.K. Hughes, C.G.K. Ziegler, S.W. Kazer, A. Gaillard, K.E. Kolb, A.-C. Villani, C.M. Johannessen, A.Y. Andreev, E.M. Van Allen, M. Bertagnolli, P.K. Sorger, R.J. Sullivan, K.T. Flaherty, D.T. Frederick, J. Jané-Valbuena, C.H. Yoon, O. Rozenblatt-Rosen, A.K. Shalek, A. Regev, L.A. Garraway, Dissecting the multicellular ecosystem of metastatic melanoma by single-cell RNA-seq, Science (New York, N.Y.), 352 (2016) 189-196.

[51] P. Brennecke, S. Anders, J.K. Kim, A.A. Kołodziejczyk, X. Zhang, V. Proserpio, B. Baying, V. Benes, S.A. Teichmann, J.C. Marioni, M.G. Heisler, Accounting for technical noise in single-cell RNA-seq experiments, Nat Methods, 10 (2013) 1093-1095.

[52] D. Aran, A.P. Looney, L. Liu, E. Wu, V. Fong, A. Hsu, S. Chak, R.P. Naikawadi, P.J. Wolters, A.R. Abate, A.J. Butte, M. Bhattacharya, Reference-based analysis of lung single-cell sequencing reveals a transitional profibrotic macrophage, Nature Immunology, 20 (2019) 163-172.

[53] Z. Zhang, D. Luo, X. Zhong, J.H. Choi, Y. Ma, S. Wang, E. Mahrt, W. Guo, E.W. Stawiski, Z. Modrusan, S. Seshagiri, P. Kapur, G.C. Hon, J. Brugarolas, T. Wang, SCINA: A Semi-Supervised Subtyping Algorithm of Single Cells and Bulk Samples, Genes (Basel), 10 (2019).

[54] S. Jin, C.F. Guerrero-Juarez, L. Zhang, I. Chang, R. Ramos, C.-H. Kuan, P. Myung, M.V. Plikus, Q. Nie, Inference and analysis of cell-cell communication using CellChat, Nature Communications, 12 (2021) 1088.

This study explores black phosphorus (BP) nanosheets in breast cancer models, showing their ability to inhibit tumor growth. BP-PEG enhances immune responses by promoting neutrophil recruitment and reprogramming tumor-associated neutrophils to an anti-tumor phenotype, boosting both innate and adaptive anti-tumor immune activities. Combining BP therapy with immunotherapies or chemotherapies potentially counteract neutrophil-induced immunosuppression, restoring or enhancing their therapeutic efficacy.

*Jing Wang, Weiqiang Yu, Hui Shen, Yanxiang Sang, Hongjie Zhang, Benyan Zheng, Xue Peng, Yuan Hu*, Xiaopeng Ma*, Zhenye Yang*, Fazhi Yu**

**Therapeutic Black Phosphorus Nanosheets Elicit Neutrophil Response for Enhanced Tumor Suppression**


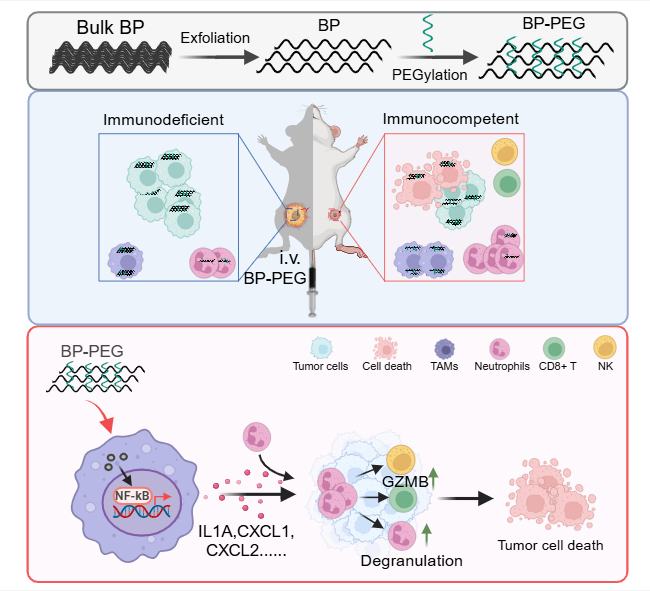


(Created in BioRender. Yu, F. (2024), Agreement number: *TE27PIVUES*).

Supporting Information

**Therapeutic Black Phosphorus Nanosheets Elicit Neutrophil Response for Enhanced Tumor Suppression**

*Jing Wang, Weiqiang Yu, Hui Shen, Yanxiang Sang, Hongjie Zhang, Benyan Zheng, Xue Peng, Yuan Hu*, Xiaopeng Ma*, Zhenye Yang*, Fazhi Yu**


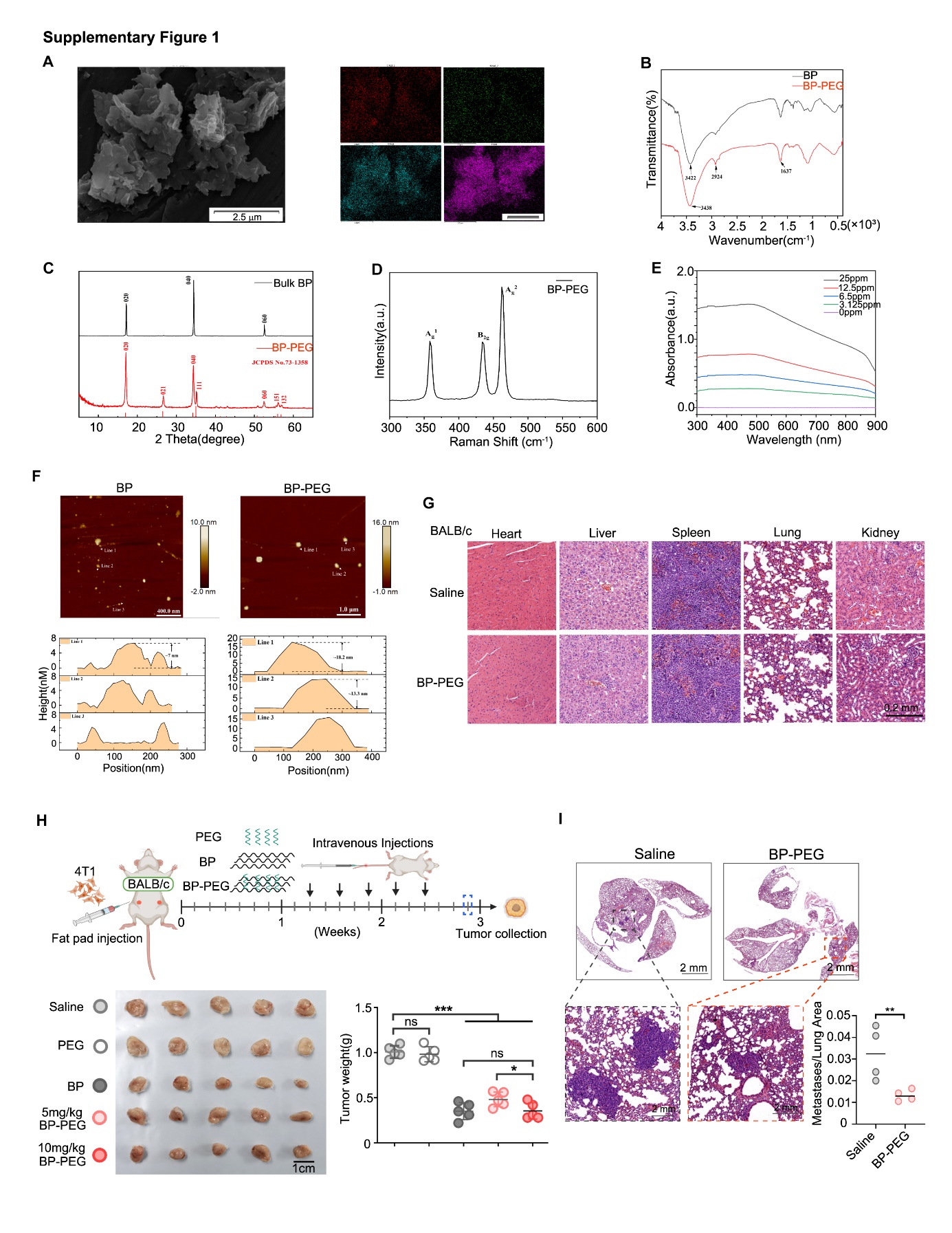


**Supplementary Figure S1.** Additional characterization of BP-PEG and assessment of toxicity and metastasis. A) SEM and elemental mapping of BP-PEG. Scale bar: 2.5μm. B) Fourier-transform infrared (FTIR) spectra of BP and BP-PEG. C) X-ray diffraction (XRD) patterns of bulk BP and BP indicating successful exfoliation into multilayer nanosheets. D) Raman spectra of BP-PEG. E) UV-visible absorption spectra of BP at different concentrations. F) Atomic force microscopy (AFM) was used to measure the thickness of BP. G) Histological analysis (H&E staining) of major organs (heart, liver, spleen, lung, and kidney) from mice treated with BP-PEG. H) A schematic diagram of the experimental design is provided. 4T1 tumor-bearing BALB/c mice were treated with 200 µL PEG, BP (10 mg/kg), or BP-PEG (10 mg/kg) (n=5 per group). Tumor images were captured, and tumor weights were measured and analyzed at the end of the study. I) Representative images of lung tissues stained with H&E. Data are presented as mean ± SD, p values were determined by two-tailed student’s t test; ns, not significant; *p < 0.05, ***p < 0.001.


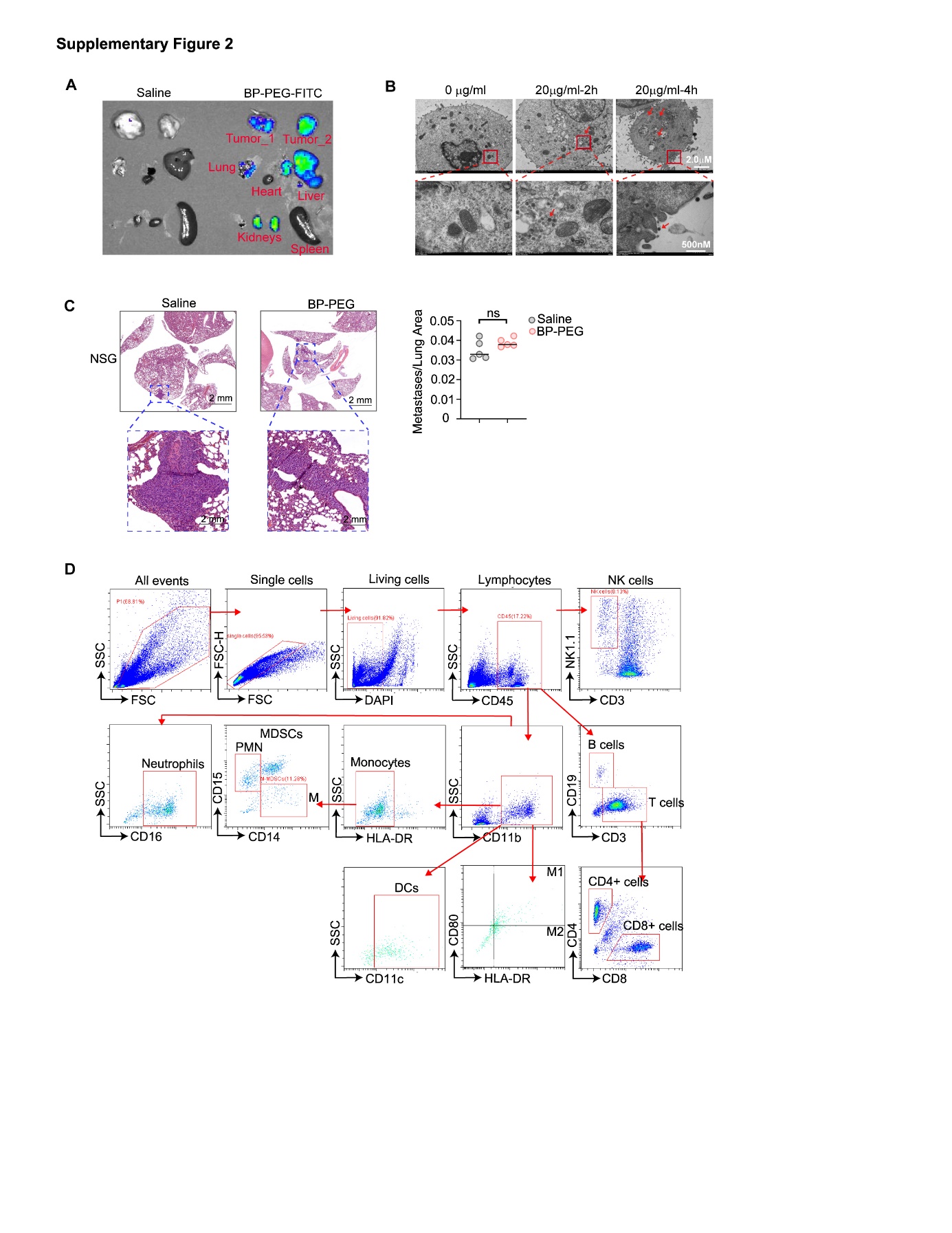


**Supplementary Figure S2.** BP-PEG accumulation and immune cell composition analysis. A) In vivo fluorescence imaging showing the accumulation of FITC-labeled BP-PEG in different organs and tumors of 4T1 tumor-bearing mice 24 hours post-injection. B) Transmission electron microscopy (TEM) images confirming the uptake of BP-PEG by 4T1 tumor cells in vitro. C) Representative H&E-stained lung sections from NSG mice bearing 4T1 tumors treated with BP-PEG or saline. D) Flow cytometry analysis of PBMCs isolated from breast cancer patients after co-culture with MDA-MB-231, showing the composition of immune cell subsets. Data are presented as mean ± SD, p values were determined by two-tailed student’s t test; ns, not significant.


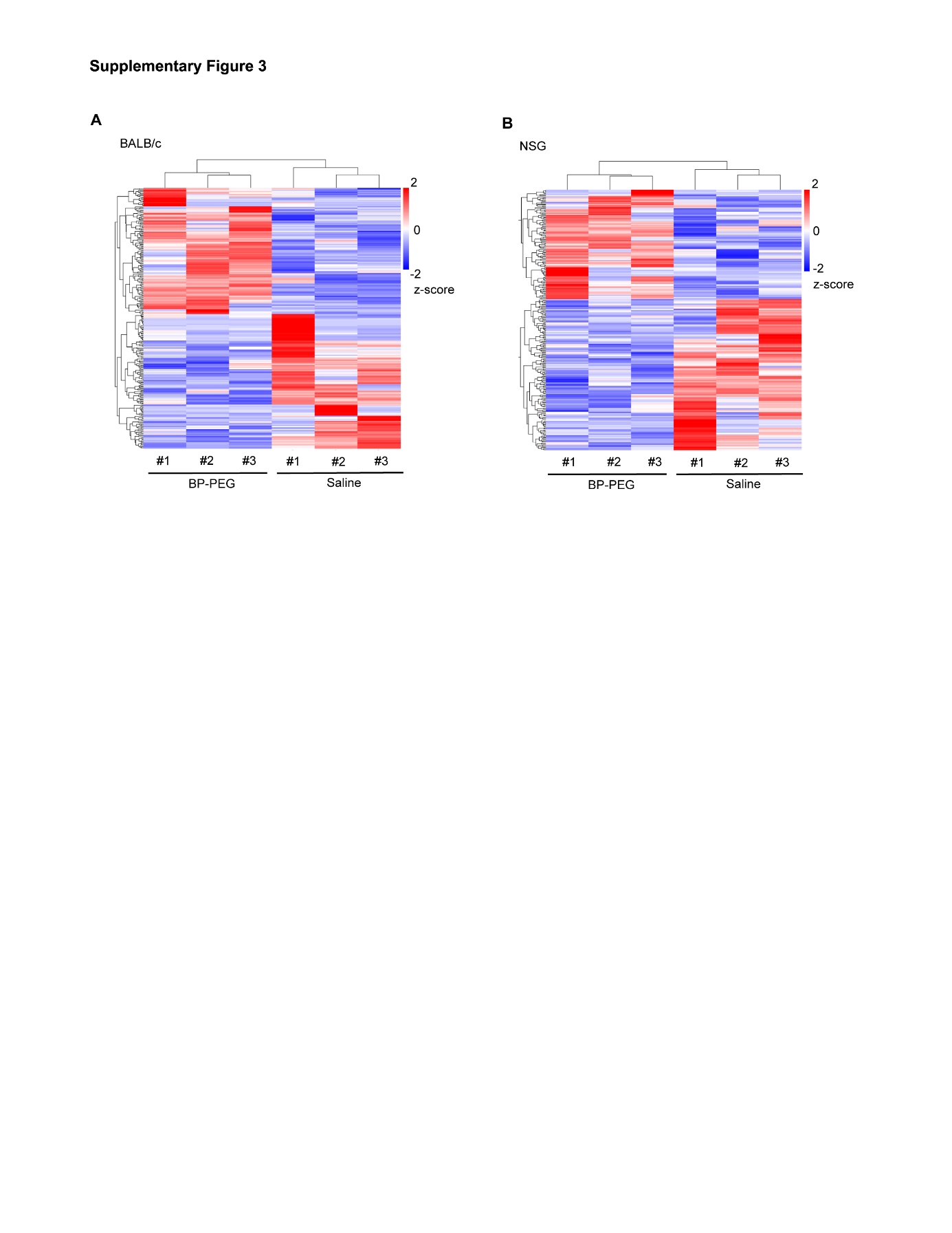


**Supplementary Figure S3.** Gene expression profiles altered by BP-PEG treatment. A) Heatmap showing the DEGs in tumors from BALB/c mice treated with BP-PEG compared to saline controls. B) Heatmap of DEGs in tumors from NSG mice treated with BP-PEG compared to saline controls.


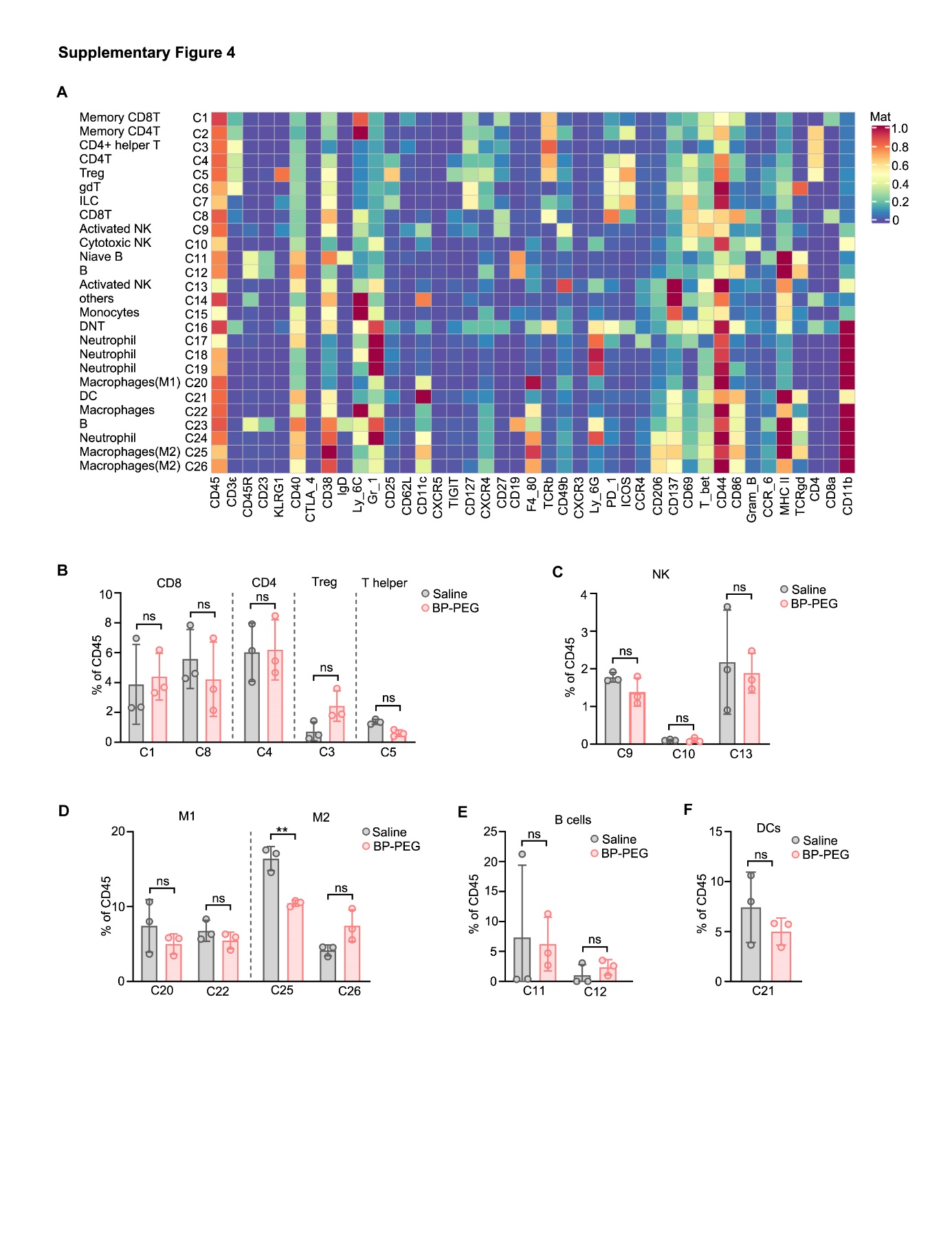


**Supplementary Figure S4.** Analysis of immune cell populations and function after BP-PEG treatment. A) Heatmap of markers expression used to identify immune cell clusters in CyTOF analysis. B–F) Proportions of other immune cell types, including T cells (B), NK cells (C), macrophages(D), B cells(E) and dendritic cells(F). Data are presented as mean ± SD, p values were determined by two-tailed student’s t test; ns, not significant; **p < 0.01.


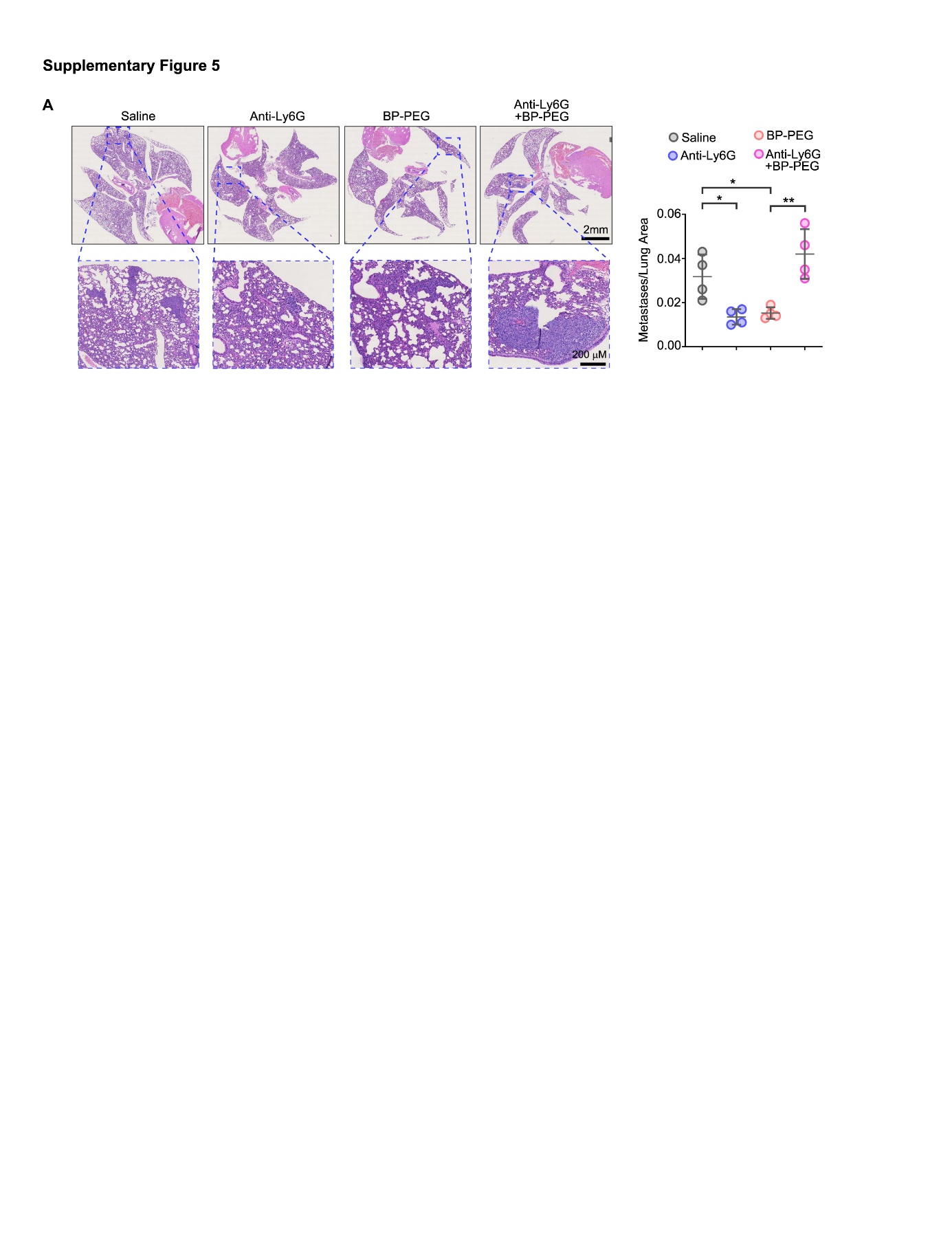


**Supplementary Figure S5.** Effect of neutrophil depletion on lung metastasis. A) Representative H&E-stained lung sections from each treatment group, showing that neutrophil depletion increases lung metastasis despite BP-PEG treatment. Data are presented as mean ± SD, p values were determined by two-tailed student’s t test; ns, not significant; *p < 0.05, **p < 0.01.


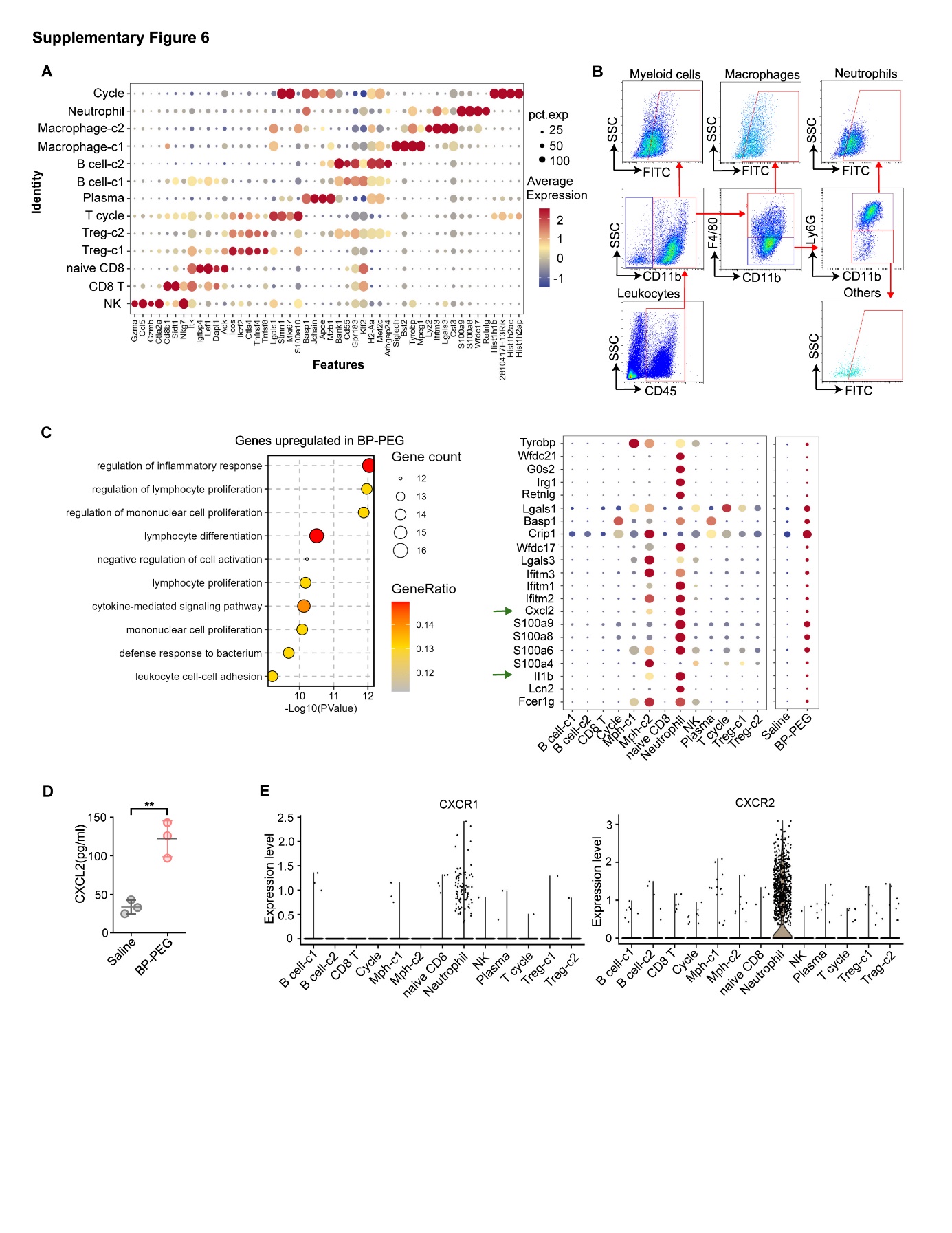


**Supplementary Figure S6.** Gene expression and cytokine analysis. A) Marker genes used to annotate cell types. Dot size represents % of cells of that cluster expressing the given gene, whereas color indicates the expression level of that cluster. B) Flow cytometry gating strategy for detecting BP-PEG-FITC uptake by various myeloid cells in tumor tissues. C) The pathway enrichment of DEGs between saline and BP-PEG treated CD45 cells isolated from the tumors, and dot plot to show the main DEGs. D) ELISA analysis of CXCL2 levels in the conditioned medium of THP-1 cells pretreated with or without 5 µg/mL BP-PEG for 12 hours. E) CXCR1 and CXCR2 expression in immune cells was analyzed using scRNA-seq data. Data are presented as mean ± SD, p values were determined by two-tailed student’s t test; ns, not significant; **p < 0.01.


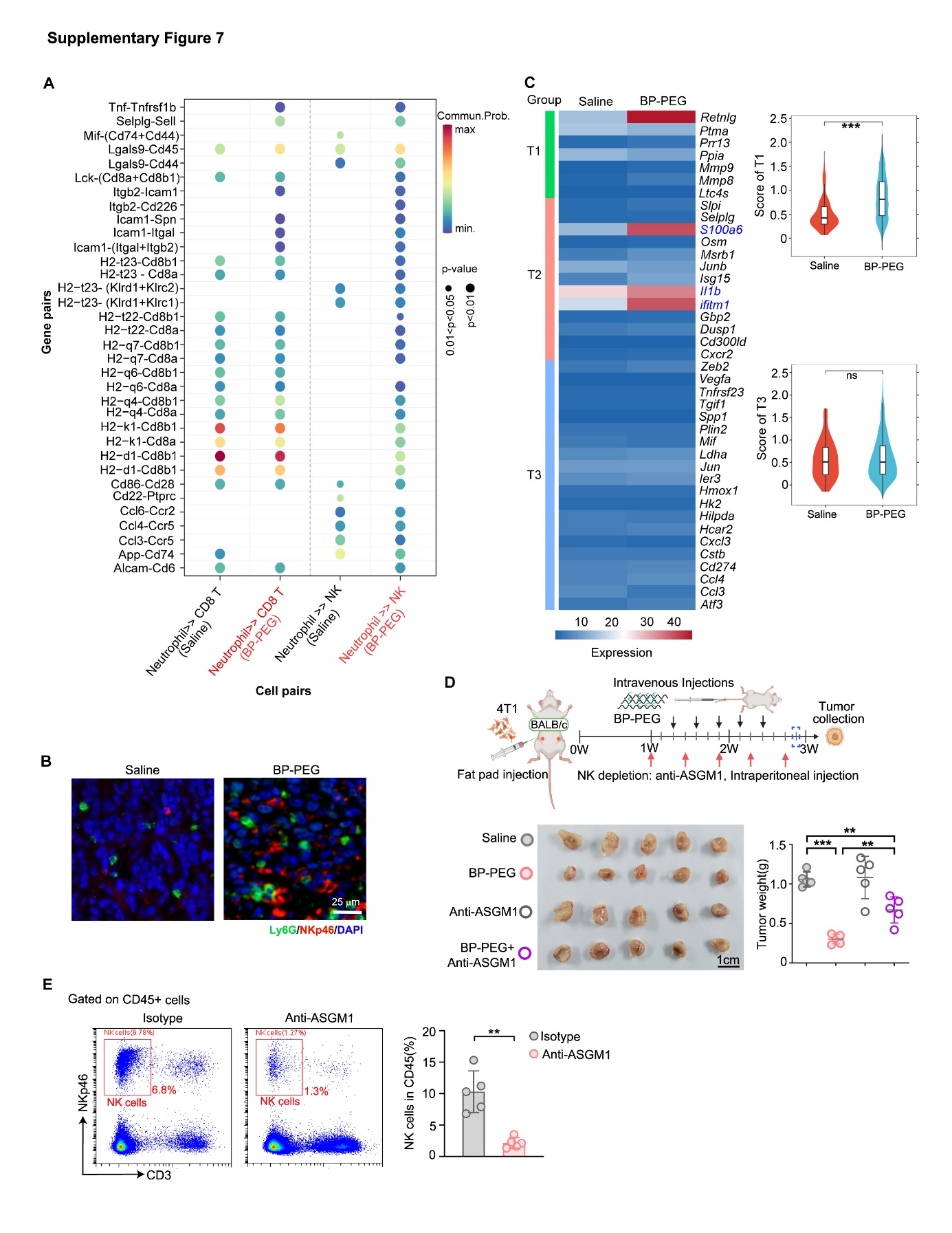


**Supplementary Figure S7.** Interaction analysis between neutrophils and T cells. A) Ligand-receptor interactions between neutrophils and CD8⁺ T cells using upregulated genes from BP-PEG treatment relative to saline treatment. B) Immunofluorescence staining of Ly6G, NKp46, and DAPI in tumor tissues derived from 4T1 cells. C) Gene signature expression analysis in neutrophils. D) A schematic diagram of the experimental design is provided. Saline, Anti-ASGM1 (25 µg), and BP-PEG (10 mg/kg) were administered to 4T1 tumor-bearing BALB/c mice in separate treatment groups (n=5 per group). Tumor images were captured, and final tumor weights were measured and analyzed at the end of the study. E) Flow cytometry was used to detect the percentage of NK cells within the CD45^+^ cell population isolated from 4T1-derived tumors. Data are presented as mean ± SD; p values were determined by two-tailed student’s t test; **p < 0.01, ***p < 0.001.
